# Process analysis of pluripotent stem cell differentiation to megakaryocytes to make platelets applying European GMP

**DOI:** 10.1038/s41536-021-00138-y

**Published:** 2021-05-26

**Authors:** Moyra Lawrence, Amanda Evans, Thomas Moreau, Marta Bagnati, Matthew Smart, Enas Hassan, Jahid Hasan, Monica Pianella, Julie Kerby, Cedric Ghevaert

**Affiliations:** 1 Cambridge Stem Cell Institute, Jeffrey Cheah Biomedical Centre, Cambridge Biomedical Campus, Puddicombe Way, Cambridge, UK; 2 Department of Haematology and NHS Blood and Transplant, University of Cambridge, Cambridge, UK; 3 Bit Bio, Discovery Drive, Cambridge Biomedical Campus, Cambridge, UK; 4 Cell and Gene Therapy Catapult, 12th Floor Tower Wing, Guy’s Hospital, Great Maze Pond, London, UK

**Keywords:** Haematopoietic stem cells, Stem-cell differentiation, Stem-cell therapies, Regenerative medicine

## Abstract

Quality, traceability and reproducibility are crucial factors in the reliable manufacture of cellular therapeutics, as part of the overall framework of Good Manufacturing Practice (GMP). As more and more cellular therapeutics progress towards the clinic and research protocols are adapted to comply with GMP standards, guidelines for safe and efficient adaptation have become increasingly relevant. In this paper, we describe the process analysis of megakaryocyte manufacture from induced pluripotent stem cells with a view to manufacturing in vitro platelets to European GMP for transfusion. This process analysis has allowed us an overview of the entire manufacturing process, enabling us to pinpoint the cause and severity of critical risks. Risk mitigations were then proposed for each risk, designed to be GMP compliant. These mitigations will be key in advancing this iPS-derived therapy towards the clinic and have broad applicability to other iPS-derived cellular therapeutics, many of which are currently advancing towards GMP-compliance. Taking these factors into account during protocol design could potentially save time and money, expediting the advent of safe, novel therapeutics from stem cells.

## Introduction

### Platelet transfusions and cross-matching

Platelets are 2–4 μm anucleate discoid cells in the bloodstream which are responsible for clotting, wound healing, inflammation at the site of injury and subsequent angiogenesis^1^. Thrombocytopenia is the lack of sufficient quantities of platelets and can result from trauma, surgery, cancer treatment, or acquired or inherited bone marrow failure^2–4^. Patients with thrombocytopenia are at risk of haemorrhage. To prevent this, they are transfused with donor platelets. Every year, 280,000 platelet units are transfused into patients in the UK. Patients who have been exposed to platelet alloantigens through transfusion or pregnancy can develop immunity to these antigens, predominantly HLA Class I, making matching platelet transfusions more challenging^5–7^. Furthermore, platelets have a shelf life of only 5–7 days, meaning that at times of restricted donor availability (holidays, natural disasters, pandemics), platelet shortages can occur^8^.

### Megakaryocytes: the mother cells for platelets

The cells which produce platelets are called megakaryocytes (MKs). MKs are large, polyploid, multinucleated cells which differentiate from haematopoietic stem cells in the bone marrow^9–11^. MKs make up 0.01–0.03% of nucleated bone marrow cells^12^; however, collectively they are estimated to produce 1–2 × 10^11^ platelets daily^13,14^ with each MK estimated to produce up to 4000 platelets^15^. Therefore, if we could generate platelet-producing MKs in vitro, we could rapidly generate platelet units for transfusion into patients.

MKs are large and not very proliferative so isolating them in meaningful numbers from the bone marrow is not technically feasible. CD34^+^ HSCs can be isolated from cord blood and initially these cells represented a promising source for MK differentiation^16–21^. However, cords are quite small and variable in CD34^+^ cell content. Adult peripheral blood generally provides even fewer HSCs than cord blood^21^ and whereas these numbers can be boosted by cytokine-mediated mobilisation of HSCs from the bone marrow, this puts the donor at increased risk of stem and progenitor exhaustion^22,23^. In addition, until recently, HSCs were challenging to expand in vitro, making it difficult to produce large numbers of HSCs as a starting material for MK differentiation^24–26^.

### Induced pluripotent stem cells as a starting material for cellular therapies

The advent of pluripotent stem cell culture provided an infinitely expandable cell source from which to differentiate MKs. Human embryonic stem cells (ESCs) can be efficiently and reproducibly differentiated into MKs in vitro^27–31^. However, ESCs can potentially pose significant ethical concerns in some parts of the world so when induced pluripotent stem cells (iPSCs) were first generated from human dermal fibroblasts, these became the starting material of choice for many cell differentiation protocols^32,33^. ESC differentiation protocols applied to iPSCs generated MKs very successfully. The insight into the requirement for MYC during MK progenitor generation^34^ initiated the differentiation of MKs from iPSCs using viral transgenes^35,36^, a process called forward programming. In parallel, work continued on cytokine-mediated directed differentiation of MKs^27,37^ and as a result, many protocols emerged which could generate MKs from iPSCs in vitro^38,39^.

iPSCs also represent a unique opportunity to generate platelets with added benefits. iPSCs can be easily gene edited, allowing the deletion of key immune signalling molecules or the addition of desired molecules, for example angiogenic compounds or clotting agents, to platelet granules. β2 microglobulin is crucial for the cell-surface expression of MHC Class I. Depleting or deleting β2 microglobulin in iPSCs generates HLA Class I null platelets which could be transfused into any recipient, including those with antibodies against HLA Class I alloantigens. β2 microglobulin had previously been depleted using short hairpin RNA in CD34^+^ cord blood derived HSCs^40^. The HSCs were then differentiated into MKs, producing platelets^40^ which could evade immune destruction^41^. This knockdown technology was applied to iPSCs to generate HLA Class I reduced platelets^42^. Clustered regularly interspaced short palindromic repeat-associated protein 9 (Cas9) was used to delete β2 microglobulin in iPSCs, producing HLA Class I knockout platelets^39^, which could evade recognition by human anti-HLA antibodies in a mouse model^43^. Additionally, the absence of HLA Class I was thought to pose a risk for platelet rejection due to Natural Killer Cell activation. The authors infuse the mouse model with human NKs and show no effect on HLA Class I knockout platelet viability, demonstrating HLA Class I knockout platelets as a viable clinical alternative for alloimmunised patient transfusion^43^.

### Platelet production from MKs using bioreactors

To generate platelet units for transfusion, efficient methods are required for platelet release from MKs generated in vitro. Many systems have already been generated, including silk scaffolds which mimic the bone marrow^44,45^, large paddles which generate the turbulence necessary to induce platelet formation^46^, flow chambers which bud platelets through a fenestrated membrane into a second chamber^47^ and collagen sponges which trap MKs and allow nascent platelets to flow through^48^. In vitro platelet production remains less efficient than in vivo; however, these systems can produce large numbers of mature, functional platelets and as systems are refined, efficiencies are increasing.

### The requirement for manufacturing standards

A patient’s quality of life and future health conditions could depend on the therapeutics they receive today, thus there is an enormous responsibility on the clinical therapeutic manufacturer to ensure stability, safety, batch-to-batch consistency and reproducibility. In order to do this, Good Manufacturing Practice (GMP) principles were introduced and applied to the manufacturing process for clinical products. To meet GMP standards, products must be of a consistently high quality, be appropriate for their intended use and meet the requirements of the marketing authorisation (MA) or product specification^49^. In the UK, the Medicines and Healthcare Products Regulatory Agency (MHRA) carries out inspections to ensure manufacturing sites comply with GMP requirements, as set out by the European Commission^49,50^. Manufacturers which pass the inspection receive a GMP certificate^49^.

Platelets may be classified an Advanced Therapy Medicinal Product (ATMP), as they are a “medicine for human use based on genes, tissues or cells”^51,52^. In Europe, the iPSCs used as starting material must be manufactured in compliance with the principles of EU GMP and donation, procurement and testing must comply with the EU Tissues and Cells Directive^53,54^ or the EU blood directive^55^, as applicable. GMP principles should apply after the donation of the cells, right through the manufacturing process^56^. Viral and transmissible spongiform encephalopathy safety requirements apply during starting material qualification and early production processes including reprogramming^57,58^. Freedom to Operate is also a crucial element to consider when selecting iPSCs; their origin, reprogramming method and the technologies used in their modification and differentiation must all be taken into account to inform the choice of line^59^. Clinical grade iPSC lines need to demonstrate certain physical, chemical, and biological properties that ensure quality and safety of the final product. There is little agreement as to the specific assays used to measure these critical quality attributes; however, generally properties for consideration include identity, potency, viability, genetic fidelity and stability, and microbiological sterility. The Global Alliance for iPSC Therapies (GAiT) has published initial recommendations on Critical Quality Attributes (CQAs) for clinical-grade iPSC lines with discussion around relevant assays for elucidating each CQA^60,61^. iPSC lines manufactured to GMP are in short supply, due to the costs involved in their production^62^, further complicating the patent landscape.

GMP applies to the entire process of manufacturing, with the level of GMP requirements increasing from early to later steps in product manufacture. There are no legal requirements for raw materials to be manufactured to GMP. Although manufacturing under a quality management system (QMS) can provide assurance of the material quality, the therapy developer is ultimately responsible for assessing the suitability of the chosen raw materials for the intended use and the adequate level of QMS. It is recommended the raw materials used are of pharmaceutical or pharmacopeial grade. Where this is not possible, risk assessments and the implementation of additional routine testing may be required^58,63^. For biologically-derived raw materials this could entail having a reliable test for biological activity. The therapy developer must also ensure full traceability of the raw and starting materials used for production. Traceability data must be retained for a minimum of 30 years after the expiry date of the product or for a longer period if provided in the marketing authorisation^58^. Every step from material sourcing and qualification, through product manufacture and storage to transport must be documented^50,58^. Documentation must include specifications, manufacturing formulae, processing and packaging instructions, procedures, protocols, records, batch records, technical agreements and certificates of analysis^58,64^. Product specification file and batch documentation must be retained for one year after batch expiry or 5 years after QP certification. For investigational medicinal products, documentation must be kept for 5 years after completion or formal discontinuation of the last clinical trial^50,58^. A quality assurance system tracks manufacturing materials, ensuring correct storage and adherence to protocols^50,58^. Qualified personnel are responsible for the implementation of this system on each manufacturing site and must have access to quality control laboratories for analysis. Manufacturing must be performed in an identical fashion for each batch and any variations to the protocol need to be re-validated^50^. Any deviation from standard practice during batch production must be approved by a qualified person^58^. GMP compliant premises must separate sterile from non-sterile workflow and clean areas must be compliant with ISO 14644-1, have controlled and tested air quality and be frequently monitored^58^. Systems used for manufacturing should be closed, if possible, to minimise contamination risk, and all final products must be sterility tested^58^. The final product must have full traceability and be labelled accordingly^50^ and is held until the release specification has been met^58^. Such release criteria begin with sterility and testing to ensure patient safety, and progress to include testing other quality attributes, such as potency. The batch is also checked to make sure the raw materials and manufacturing procedures correspond to the protocols. Batch release is carried out by the qualified person and the details registered. In general, three consecutive batches must be manufactured to validate the process^58^.

### Results of process adaptation

**Fig. 1:**
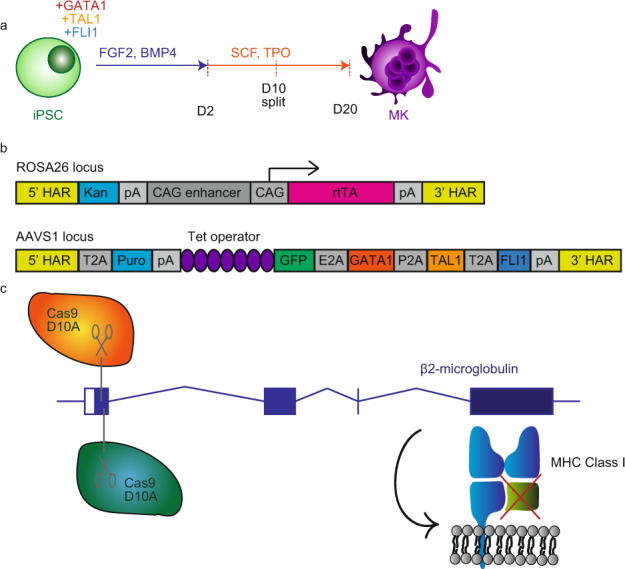
Generating megakaryocytes from iPSCs. **a** Overview of the Megakaryocyte Forward Programming protocol. iPSCs overexpressing GATA1, TAL1 and FLI1 are differentiated in FGF2 and BMP4 for 3 days and then in SCF and TPO for 17 days to generate mature CD41^+^CD42^+^ megakaryocytes (MKs). **b** Schematic of the inducible cassette for GATA1, TAL1 and FLI1 expression in iPSCs and their patterning into MKs. One cassette, containing rTTA, is inserted in the ROSA26 locus while a second cassette containing the patterning transcription factors is integrated into the AAVS1 locus. **c** Schematic of HLA Class I knockout strategy, using paired Cas9 D10A nickases to create a frameshift mutation in β2 microglobulin.

In order to produce platelets from iPSCs for transfusion, we first need to adapt the protocol to meet GMP requirements. This paper focuses on the first part of the process; the differentiation of iPSCs to mature MKs. In the laboratory, we have designed a reproducible and efficient method for producing mature MKs from iPSCs, using three patterning transcription factors and only two cytokine combinations (Fig. 1a)^36^. Within 20 days, the culture becomes a pure culture of mature MKs, capable of producing functional platelets which can contribute to thrombus formation^36^. We have repurposed a previously described integrated transgene approach^65^ where the 3 patterning transcription factors are inserted into the AAVS1 safe harbour locus and the reverse tetracycline transactivator (rtTA) is stably inserted into the ROSA26 locus (Fig. 1b). Both these safe harbour loci have been used extensively in mouse and human cells and are consistently and robustly expressed without compromising survival^66^. Dual antibiotic selection enables the quick and easy selection of dually targeted clones^65^. Upon the addition of doxycycline, the transcription factors are expressed and begin patterning the iPSCs to MKs^67,68^. Working with iPSCs has also allowed us to take advantage of the Cas9 system to knock out β2 microglobulin using two guide RNAs and Cas9 nickase D10A protein (Fig. 1c)^69^.

**Fig. 2:**
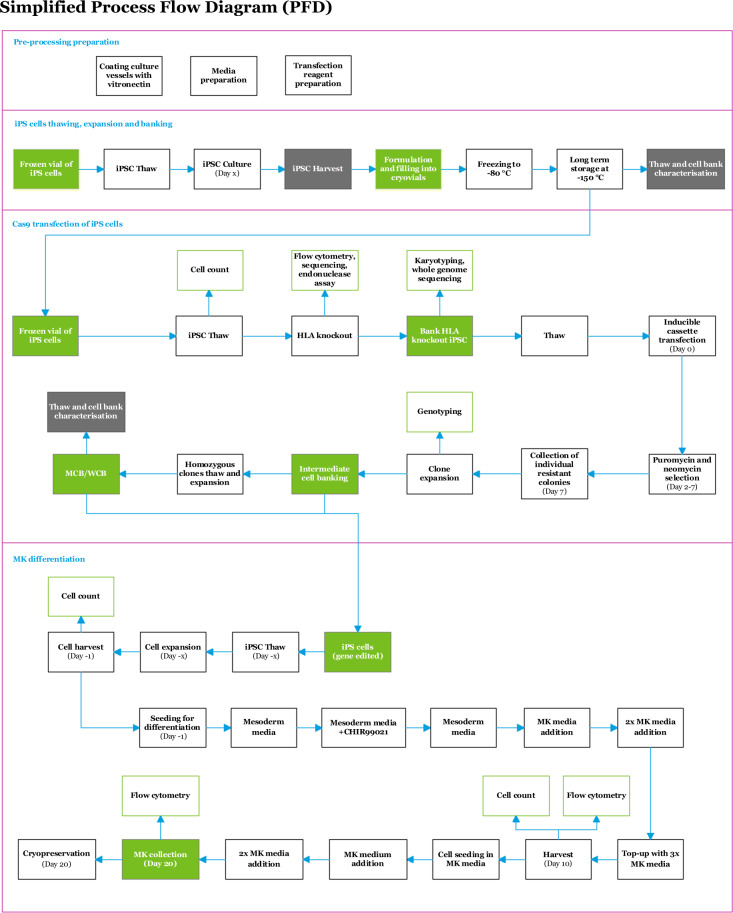
Process flow diagram of the megakaryocyte forward programming protocol. The days of the protocol on which each step takes place are indicated.

We had previously carried out several years’ work transferring the protocol from reagents intended for research use only to reagents manufactured under GMP or a suitable QMS, achieving similar MK production efficiencies by calculated substitution. We then employed Cell and Gene Therapy Catapult to carry out process mapping, working from detailed protocols for each stage of the process. The resulting process diagram (Fig. 2) shows the high level overview of the process and is divided into 4 sections:

1. Pre-processing preparation
2. iPSC thawing, expansion and banking
3. Cas9 transfection of iPSCs (HLA knockout and inducible cassette transfection)
4. MK differentiation

The last three sections are connected by key cell banking steps: first the banking of unedited iPSCs and then the generation of master and working cell banks of HLA Class I knockout, inducible iPSCs. Batch sequence diagrams were then generated of the entire process (Supplementary Fig. 1), involving:

1. HLA knockout
2. Inducible cassette transfection
3. MK differentiation

**Fig. 3:**
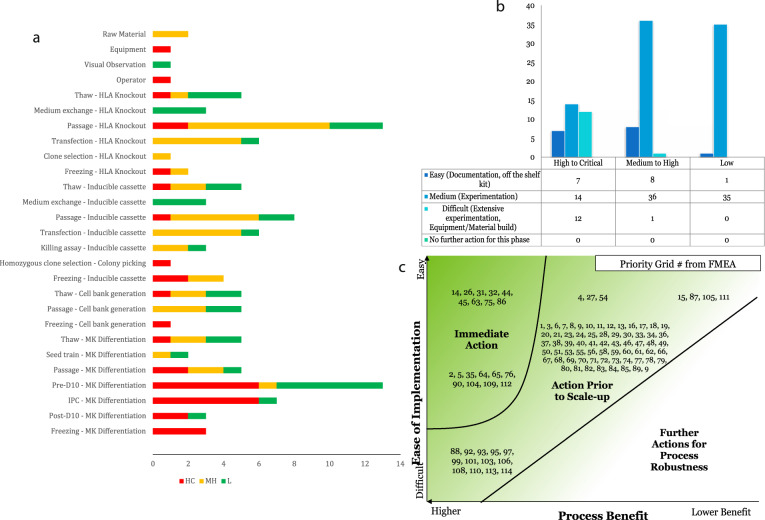
Failure mode and effect analysis. **a** Segregation of identified risks by processing step and Risk Priority Code (RPC). Risks were scored as (L) Low risk, RPN < 40 and Severity < 3, (MH) Medium to High risk, Severity ≥3 or RPN ≥ 40 and ≤75 or (HC) High to Critical risk, RPN ≥ 75. **b** Segregation of identified risks by Risk Priority Code (RPC) score and ease of implementation for proposed mitigation strategies. Risks were scored as in A. Ease of implementation was scored as follows: (1) Documentation/Training/Just do it/Purchase kit. Does not change RPC and/or does not improve, (3) Experimentation/Vendor contact/Technical Agreement/Changes to BMR. Reduces RPC from MH to L and/or results in some improvement in manufacturability, (5) Long-term development/Extensive experimentation/Machining/User-defined kit/Changes to regulatory submissions. Reduces RPC from HC to MH or L and/or results in significant improvement in manufacturability. **c** FMEA Priority Number (PN) Grid Map. This chart plots the priority number associated with each identified risk in relation to the ease of implementation and process benefit of its associated mitigation strategy. In turn this provides an initial insight into those activities that should be given high, medium or low priority with respect to developing the prioritisation plan.

The first analysis we can perform using this information is Failure Mode and Effect Analysis (FMEA). FMEA pinpointed 114 specific risks across the entire process and these risks are shown in Fig. 3a, coloured by risk prioritisation. We then analysed how difficult these risks were to mitigate: 14% could be addressed through straightforward activities (updating documentation, purchasing off-the-shelf alternative products, the development and implementation of training packages). 75% could be mitigated with a medium level of effort (experimental activities to test the impact of process changes or building knowledge around the design space of specific unit operations). The remaining 11% of risk mitigation strategies were considered challenging to implement and mostly concerned differentiation process scaling.

In order to establish a prioritisation plan, the ease of mitigation was then compared to the benefit of mitigation to the process (Fig. 3b). Risks were then plotted on a priority grid of process benefit versus ease of implementation (Fig. 3c, Supplementary Fig. 2). For each number in the priority grid, the process step and potential failure mode were detailed. Those labelled “Immediate Action” should be the first to be addressed. Ishikawa diagrams enabled the identification of the cause of each risk (Supplementary Fig. 3). A detailed plan was then devised for each risk (Supplementary Fig. 2) and taking into account the current controls in place for each risk, further controls were designed and their ease of implementation and process benefit were scored. This analysis will be essential in risk prioritisation, allowing us to mitigate the most critical risks with the largest impact on the process first.

**Fig. 4:**
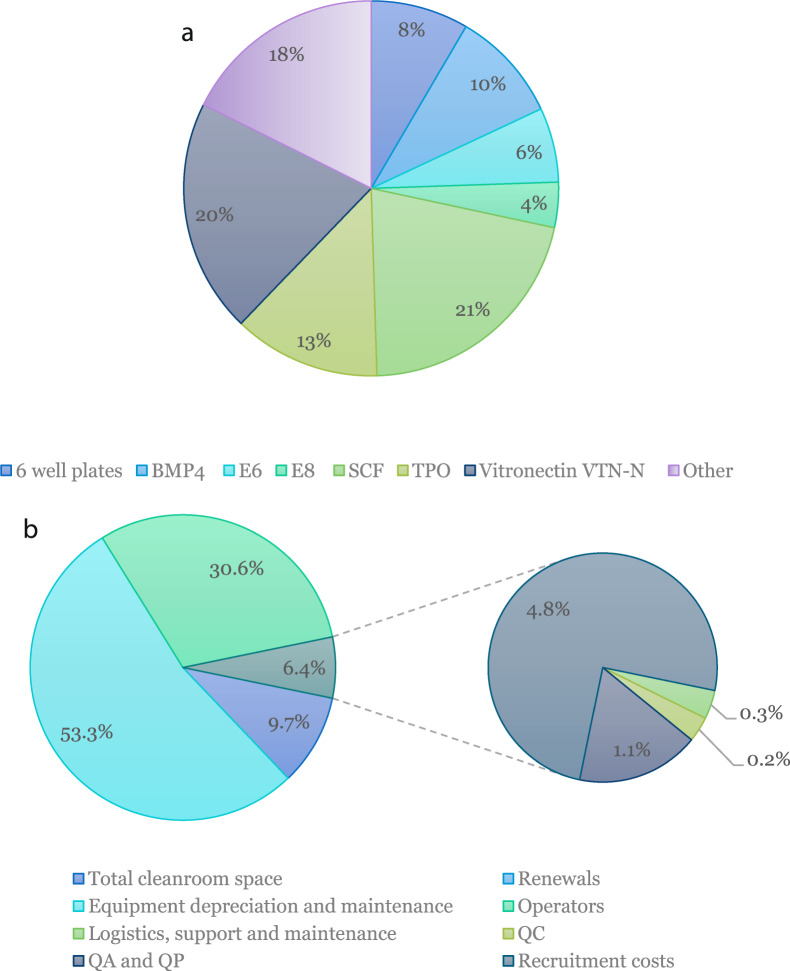
Cost of materials and facility costs of manufacture. **a** Breakdown of the reagents and consumables having the most significant contribution to the cost of reagents (CoRs). BMP4 Bone Morphogenic Protein 4, E6 and E8: iPSC media, SCF Stem Cell Factor, TPO Thrombopoietin. **b** Fixed cost break down after year 5 after production ramps up to meet consistent yearly dose requirement.

As well as its quality and efficacy, the cost of a product is crucial in determining its success. To analyse this, a costing model was created using the assumptions detailed in Tables 1 and 2 and built to include use of a GMP facility, staff costs and variable costs. First of all, reagent costs were plotted (Fig. 4a). Most costs come from cytokines manufactured to GMP. Work is currently taking place to optimise media exchange so as to promote maximal differentiation with minimal input cytokines, so this work may help reduce cost. Following this, facility costs were calculated per batch (consisting of a single platelet unit). This totalled more than £39,000, split by percentage in Fig. 4b. In order to decrease these costs, throughput could be increased, making it tempting to automate at least some of the process to save hands-on time for GMP operators and tissue culture hood occupancy, which are key limiting factors for production. Assuming currently modelled manual production can be increased to 500 doses in year one, the cost of producing a single platelet unit totals £149,571. The production costs of in vitro platelets would of course preclude adoption by any healthcare system. However, driving down costs by process optimisation will inevitably make production more economically viable. In addition, these platelet units hold promise as targeted therapeutic delivery vehicles when derived from gene edited iPSCs. If they could deliver costly therapies in a targeted way, abrogating the need for systemic delivery, they could be a commercially competitive alternative to current therapies.

**Table 1:** Process assumptions.

Process assumptions	Annotations & calculations	Value
Target doses per year	Dose/yr	5000
Platelets per patient	Dose	2.4 × 10^11^
Process efficiency	*η*	50%
Doses per batch	D/B	1
Platelets per batch	P/B	4.8 × 10^11^
Platelets per Megakaryocyte (MK)	P/MK	100
MK per batch	MK/D = (P/B)/(P/MK)	4.8 × 10^9^
IPSC seeding density/cm^2^	SD_iPSC_	1.2 × 10^4^
Fold expansion (MK per iPSC)	FE	75
IPSC required per batch for differentiation	iPSC_t0_ = (MK/D)/FE	6.4 × 10^7^
Surface area required for differentiation (cm^2^)	SA_diff_ = iPSC_t0_/SD_iPSC_	5.33 × 10^3^
Vessel format		6-well plate
Vessel surface area (cm^2^)	SA_v_	57
Number of vessels start of MK diff	N_vt0_ ≈ SA_diff_/SA_v_	94
Operators at start	O_t0_ ≈ N_vt0_/36	3
Number of isolators at start	ISO_t0_ ≈ O_t0_/2	2
Number of vessels at day 12 of MK diff	N_vt12_ = 2N_vt0_	188
Operators at day 12 of MK diff	O_t0_ ≈ N_vt12_/36	6
Number of isolators at day 12 of MK diff	ISO_t12_ ≈ O_t12_/2	3
Number of vessels at day 16 of MK diff	N_vt16_ = 2N_vt12_	376
Operators start at day 16 of MK diff	O_t16_ ≈ N_vt12_/36	11
Number of isolators at day 16 of MK diff	ISO_t16_ ≈ O_t16_/2	6

**Table 2:** Model assumptions.

Model assumptions	Assumption
Hours of operation per day	16 h (2 × 8-hour shifts)
Manufacturing days per year	320
Batch success rate	100%
Ownership strategy	Rental
Air grade	B

### Process diagnostics recommendations: prioritisation plan

All the information, when combined, then allowed the synthesis of a plan in order of priority. Three areas were highlighted, in decreasing order of importance. All of these are widely applicable to the manufacturing of other cell therapies from iPSCs and other stem cells, thus we believe them to be of critical relevance to others continuing process transfer to GMP.

## Definition of the cell banks

The Master Cell Bank (MCB) is the starting cell bank, and the Working Cell Bank (WCB) is made by thawing a subset of the MCB for further expansion or processing. Both MCB and WCB must be fully qualified. The MCB could consist of either HLA knockout, inducible iPSCs or mature MKs. If the iPSCs are defined as the MCB, the entire manufacturing process from iPSC to platelet needs to be validated and the MKs characterised as a process intermediate. Conversely, if the MKs are defined as the MCB, formal validation of the upstream process may not be required once it is demonstrated to be fit for purpose. Each frozen aliquot of MKs, however, should suffice to produce at least a whole platelet unit, and given the current optimised freezing density, the volumes needed would be untenable for most freezing systems. In addition, the MCB would need to be frequently regenerated, posing manufacturing constraints for cell bank re-qualification and comparability.

## Process automation

Currently, the process is open, from which 65% of the process risks stem. Medium exchange, cell passaging and counting are done manually, posing risks for variability and subsequent batch failure. To address this, automated systems should be implemented. 78% of high risks can be eliminated by implementing automated liquid handling or bioreactor technology and a further 14% by automated cell counting. Bioreactor technology will also be key to reducing culture volume and reliance on 6 well plates. One Quantum^®^ hollow fibre system, for example, could produce an entire platelet unit at current cell densities. Furthermore, optimising both platelet production from MKs and MK production from iPSCs holds enormous potential for increasing output with the same inputs and this work is already underway in the lab^48,68^.

## Quality control of process intermediates

Our current quality control (QC) measures are based on best research practice and entail:

- Cell counting using a haemocytometer and flow cytometer
- Visual observations of the cultures to assess sterility, confluency and cell differentiation
- Flow cytometry for CD235a, CD42a and CD41a markers
- Targeted sequencing, T7 nuclease assay and flow cytometry for HLA ABC to verify the efficiency of HLA knockout and select homozygous clones
- 7-step PCR approach to verify inducible cassette integration
- Whole genome sequencing and karyotyping of the final iPSC bank.

These QC checks conform to research community standards for gene editing and iPSC and MK culture; however, manufacturing processes demand more stringent QC. As a result of process and FMEA analyses, the following additions were designed:

1) QC control points should be introduced early in the MK differentiation process in order to identify failing cultures before Day 10.

2) Sterility and mycoplasma checks should be performed regularly on batches.

3) MKs should be regularly tested for metabolic or cell surface markers which correlate to platelet production efficiency, allowing batch failure prediction before MKs are added to the bioreactor for platelet release.

4) Residual undifferentiated iPSCs also pose a risk to the final product. Given the length of the process, it is unlikely that iPSCs remain undifferentiated throughout; however, tumorigenicity assays and culture under conditions promoting pluripotency should be performed on the final product to exclude the possibility.

5) Equipment use needs to be completely reconsidered when transiting to GMP. Wet ice poses a transfer and contamination risk and likewise, aspirators are incompatible with the controlled airflow required in GMP facilities. These process steps should thus be replaced with GMP-compliant equivalents. Time to thaw or use reagents is again rarely considered in the research lab, but its specification could be crucial to batch success in a GMP process. Similarly, the definition of room temperature and how long various reagents take to equilibrate to room temperature must be noted and incorporated into protocols.

All of these considerations apply broadly to many other iPSC-derived cellular therapeutics and scientists designing other GMP protocols could benefit hugely from factoring them early into process designs.

## Conclusions

Process mapping and analysis of the entire differentiation protocol from iPSC to MK has provided us with an insight into how MK differentiation should be optimised for manufacturing, identified the riskiest steps for batch failure and enabled risk prioritisation. This analysis has also provided us with an estimation of the cost of each manufacturing batch, as well as important considerations for scaling up and automation. As with many research processes which are adapted to meet GMP requirements, there are several over-arching themes which this analysis highlights.

First of all, the identification of reagents manufactured under a suitable QMS should be one of the first steps taken in the transit towards the clinic. “GMP equivalents” are difficult to find for many reagents intended for research use only and time spent in process optimisation at this stage saves costly process revalidation once the process has been transferred to a GMP facility. Second, scale and cost must be considered carefully when embarking on production of a product for the clinic. Production cost, demand for the product and the cost of current alternatives need to be factored into any analysis to provide a product with the best chance of commercial success. Third, operator scheduling plays a huge role in the cost and scale of any manufacturing process. Both operator time and equipment time make manual operations costly and risky. Processes should be transited to closed processes and automation should be considered as an avenue to reducing staffing costs, minimising equipment use and maximising the parallel production of batches. Fourth, it is crucial to seek regulatory advice early, to avoid optimising processes which will not pass basic regulatory controls. Researchers also benefit from seeking expert advice on process definition and the risks involved with even well-defined laboratory processes. “GMP equivalents” do not exist for all equipment designed for research use only and many protocols involve steps which are too poorly defined for GMP manufacturing. Thus, expert advice is warranted to create streamlined and safe manufacturing protocols which comply with GMP standards.

Thus, in conclusion, this work has enabled us to gain an overview of the entire manufacturing process from iPSC to MK, the risks involved at each stage and actions which should be taken to mitigate these risks, reduce costs and increase production efficiency. This analysis is widely applicable to cell therapy manufacture from iPSCs and many of the process alterations detailed in this work should be applied as other processes move towards the clinic. Good Manufacturing Practice allows us to generate safe products at scale, minimising risk to the patient and allowing clinical trials of new treatments and prophylactics.

## Supplementary information

Supplementary Information

## References

[1] Menter, D. G. et al. Platelet “first responders” in wound response, cancer, and metastasis. *Cancer Metastasis Rev.* **36**, 199–213 (2017). 10.1007/s10555-017-9682-0 PMC5709140 28730545

[2] Harker LA, Finch CA (1969). Thrombokinetics in man. J. Clin. Invest..

[3] Slichter SJ (1980). Controversies in platelet transfusion therapy. Annu. Rev. Med..

[4] Estcourt LJ (2017). Guidelines for the use of platelet transfusions. Br. J. Haematol..

[5] Stanworth SJ, Navarrete C, Estcourt L, Marsh J (2015). Platelet refractoriness–practical approaches and ongoing dilemmas in patient management. Br. J. Haematol..

[6] Kickler T, Kennedy SD, Braine HG (1990). Alloimmunization to platelet-specific antigens on glycoproteins IIb-IIIa and Ib/IX in multiply transfused thrombocytopenic patients. Transfusion.

[7] Kiefel V, König C, Kroll H, Santoso S (2001). Platelet alloantibodies in transfused patients. Transfusion.

[8] Hegde S, Akbar H, Zheng Y, Cancelas JA (2018). Towards increasing shelf life and haemostatic potency of stored platelet concentrates. Curr. Opin. Hematol..

[9] Yamada E (1957). The fine structure of the megakaryocyte in the mouse spleen. Acta Anat.

[10] Sola-Visner MC, Christensen RD, Hutson AD, Rimsza LM (2007). Megakaryocyte size and concentration in the bone marrow of thrombocytopenic and nonthrombocytopenic neonates. Pediatr. Res..

[11] Machlus KR, Italiano JE (2013). The incredible journey: from megakaryocyte development to platelet formation. J. Cell Biol..

[12] Nakeff A, Maat B (1974). Separation of megakaryocytes from mouse bone marrow by velocity sedimentation. Blood.

[13] Bluteau D (2009). Regulation of megakaryocyte maturation and platelet formation. J. Thromb. Haemost..

[14] Harker LA (1977). The kinetics of platelet production and destruction in man. Clin. Haematol..

[15] Kaufman RM, Airo R, Pollack S, Crosby WH (1965). Circulating megakaryocytes and platelet release in the lung. Blood.

[16] Pineault N, Robert A, Cortin V, Boyer L (2013). Ex vivo differentiation of cord blood stem cells into megakaryocytes and platelets. Methods Mol. Biol..

[17] Xi J (2013). Infusion of megakaryocytic progenitor products generated from cord blood hematopoietic stem/progenitor cells: results of the phase 1 study. PLoS One.

[18] Cortin V (2005). Efficient in vitro megakaryocyte maturation using cytokine cocktails optimized by statistical experimental design. Exp. Hematol..

[19] Shaw PH, Gilligan D, Wang XM, Thall PF, Corey SJ (2003). Ex vivo expansion of megakaryocyte precursors from umbilical cord blood CD34 cells in a closed liquid culture system. Biol. Blood Marrow Transpl..

[20] Mattia G (2002). Different ploidy levels of megakaryocytes generated from peripheral or cord Blood CD34+ cells are correlated with different levels of platelet release. Blood.

[21] van den Oudenrijn S, von dem Borne AE, de Haas M (2000). Differences in megakaryocyte expansion potential between CD34(+) stem cells derived from cord blood, peripheral blood, and bone marrow from adults and children. Exp. Hematol..

[22] Schmitz N (1996). Randomised trial of filgrastim-mobilised peripheral blood progenitor cell transplantation versus autologous bone-marrow transplantation in lymphoma patients. Lancet.

[23] Kumar PS, Chandrasekhar C, Srikanth L, Sarma PVGK (2018). In vitro large scale production of megakaryocytes to functional platelets from human hematopoietic stem cells. Biochem. Biophys. Res. Commun..

[24] Derakhshani M (2019). Strategies for elevating hematopoietic stem cells expansion and engraftment capacity. Life Sci..

[25] Tung SS, Parmar S, Robinson SN, De Lima M, Shpall EJ (2010). Ex vivo expansion of umbilical cord blood for transplantation. Best. Pract. Res. Clin. Haematol..

[26] Delaney C (2010). Notch-mediated expansion of human cord blood progenitor cells capable of rapid myeloid reconstitution. Nat. Med..

[27] Eto K (2002). Megakaryocytes derived from embryonic stem cells implicate CalDAG-GEFI in integrin signaling. Proc. Natl Acad. Sci. USA.

[28] Gaur M (2006). Megakaryocytes derived from human embryonic stem cells: a genetically tractable system to study megakaryocytopoiesis and integrin function. J. Thromb. Haemost..

[29] Takayama N (2008). Generation of functional platelets from human embryonic stem cells in vitro via ES-sacs, VEGF-promoted structures that concentrate hematopoietic progenitors. Blood.

[30] Lu SJ (2011). Platelets generated from human embryonic stem cells are functional in vitro and in the microcirculation of living mice. Cell Res..

[31] Pick M, Azzola L, Osborne E, Stanley EG, Elefanty AG (2013). Generation of megakaryocytic progenitors from human embryonic stem cells in a feeder- and serum-free medium. PLoS One.

[32] Takahashi K, Okita K, Nakagawa M, Yamanaka S (2007). Induction of pluripotent stem cells from fibroblast cultures. Nat. Protoc..

[33] Takahashi K (2007). Induction of pluripotent stem cells from adult human fibroblasts by defined factors. Cell.

[34] Takayama N (2010). Transient activation of c-MYC expression is critical for efficient platelet generation from human induced pluripotent stem cells. J. Exp. Med..

[35] Nakamura S (2014). Expandable megakaryocyte cell lines enable clinically applicable generation of platelets from human induced pluripotent stem cells. Cell Stem Cell.

[36] Moreau T (2016). Large-scale production of megakaryocytes from human pluripotent stem cells by chemically defined forward programming. Nat. Commun..

[37] Fujimoto TT, Kohata S, Suzuki H, Miyazaki H, Fujimura K (2003). Production of functional platelets by differentiated embryonic stem (ES) cells in vitro. Blood.

[38] Mills JA, Paluru P, Weiss MJ, Gadue P, French DL (2014). Hematopoietic differentiation of pluripotent stem cells in culture. Methods Mol. Biol..

[39] Feng Q (2014). Scalable generation of universal platelets from human induced pluripotent stem cells. Stem Cell Rep..

[40] Figueiredo C (2010). Generation of HLA-deficient platelets from hematopoietic progenitor cells. Transfusion.

[41] Gras C (2013). HLA-universal platelet transfusions prevent platelet refractoriness in a mouse model. Hum. Gene Ther..

[42] Börger, A.-K. et al. Generation of HLA-universal iPSCs-derived megakaryocytes and platelets for survival under refractoriness conditions. *Mol. Med.* **22**, 274–285 (2016). 10.2119/molmed.2015.00235 PMC5023513 27262025

[43] Suzuki D (2020). iPSC-derived platelets depleted of HLA Class I are inert to anti-HLA Class I and natural killer cell immunity. Stem Cell Rep..

[44] Tozzi L (2018). Multi-channel silk sponge mimicking bone marrow vascular niche for platelet production. Biomaterials.

[45] Di Buduo CA (2017). Modular flow chamber for engineering bone marrow architecture and function. Biomaterials.

[46] Ito Y (2018). Turbulence activates platelet biogenesis to enable clinical scale ex vivo production. Cell.

[47] Thon JN (2014). Platelet bioreactor-on-a-chip. Blood.

[48] Shepherd JH (2018). Structurally graduated collagen scaffolds applied to the ex vivo generation of platelets from human pluripotent stem cell-derived megakaryocytes: enhancing production and purity. Biomaterials.

[49] *Good manufacturing practice and good distribution practice*, <https://www.gov.uk/guidance/good-manufacturing-practice-and-good-distribution-practice> (2020).

[50] Commission Directive 2003/94/EC of 8 October 2003 laying down the principles and guidelines of good manufacturing practice in respect of medicinal products for human use and investigational medicinal products for human use. *Off. J. Eur. Union*., 262/222–262/226 (2003).

[51] *Advanced therapy medicinal products: regulation and licensing*, <https://www.gov.uk/guidance/advanced-therapy-medicinal-products-regulation-and-licensing> (2015).

[52] *Advanced therapy medicinal products: overview*, <https://www.ema.europa.eu/en/human-regulatory/overview/advanced-therapy-medicinal-products-overview> (2020).

[53] DIRECTIVE 2004/23/EC OF THE EUROPEAN PARLIAMENT AND OF THE COUNCIL of 31 March 2004 on setting standards of quality and safety for the donation, procurement, testing, processing, preservation, storage and distribution of human tissues and cells. *Official Journal of the European Union*, 102/148–102/158 (2004).

[54] COMMISSION DIRECTIVE 2006/17/EC of 8 February 2006 implementing Directive 2004/23/EC of the European Parliament and of the Council as regards certain technical requirements for the donation, procurement and testing of human tissues and cells. *Official Journal of the European Union*, 38/40 - 38/52 (2006).

[55] DIRECTIVE 2002/98/EC OF THE EUROPEAN PARLIAMENT AND OF THE COUNCIL of 27 January 2003 setting standards of quality and safety for the collection, testing, processing, storage and distribution of human blood and blood components and amending Directive 2001/83/EC. *Official Journal of the European Union*, 33/30–33/40 (2002).

[56] Guideline on quality, non-clinical and clinical requirements for investigational advanced therapy medicinal products in clinical trials. *Committee for Advanced Therapies (CAT)* (2019).

[57] Note for guidance on minimising the risk of transmitting animal spongiform encephalopathy agents via human and veterinary medicinal products (EMA/410/01 rev.3). *Official Journal of the European Union*, 73/71–73/18 (2011).

[58] Guidelines on Good Manufacturing Practice specific to Advanced Therapy Medicinal Products. *EudraLex* **Volume 4** (2017).

[59] Morita Y, Okura H, Matsuyama A (2019). Patent application trends of induced pluripotent stem cell technologies in the United States, Japanese, and European Applications. Biores. Open. Access.

[60] Sullivan S (2018). Quality control guidelines for clinical-grade human induced pluripotent stem cell lines. Regen. Med..

[61] Sullivan S (2020). The Global Alliance for iPSC Therapies (GAiT). Stem Cell Res..

[62] Thon JN, Medvetz DA, Karlsson SM, Italiano JE (2015). Road blocks in making platelets for transfusion. J. Thromb. Haemost..

[63] DIRECTIVE 2001/83/EC OF THE EUROPEAN PARLIAMENT AND OF THE COUNCIL of 6 November 2001 on the Community code relating to medicinal products for human use. *Official Journal of the European Communities*, 311/367–311/121 (2001).

[64] COMMISSION DELEGATED REGULATION (EU) 2017/1569 of 23 May 2017 supplementing Regulation (EU) No 536/2014 of the European Parliament and of the Council by specifying principles of and guidelines for good manufacturing practice for investigational medicinal products for human use and arrangements for inspections. *Official Journal of the European Union*, 238/212 (2017).

[65] Pawlowski M (2017). Inducible and deterministic forward programming of human pluripotent stem cells into neurons, skeletal myocytes, and oligodendrocytes. Stem Cell Rep..

[66] Pellenz S (2019). New human chromosomal sites with “Safe Harbor” potential for targeted transgene insertion. Hum. Gene Ther..

[67] Dalby A (2018). Transcription factor levels after forward programming of human pluripotent stem cells with GATA1, FLI1, and TAL1 determine megakaryocyte versus erythroid cell fate decision. Stem Cell Rep..

[68] Evans, A. et al. Transfer to the clinic: refining forward programming of hPSCs to megakaryocytes for platelet production in bioreactors. *Blood Adv.**(**in press**)* (2021). 10.1182/bloodadvances.2020003236 PMC8045491 33843988

[69] Mali P (2013). CAS9 transcriptional activators for target specificity screening and paired nickases for cooperative genome engineering. Nat. Biotechnol..

