# Supplementary material for: Process analysis of pluripotent stem cell differentiation to megakaryocytes to make platelets applying European GMP
Source: NPJ Regen Med. 2021 May 26;6:27. doi: 10.1038/s41536-021-00138-y (PMC8155004; doi:10.1038/s41536-021-00138-y)
Supplement: Supplementary file 1 — Supplementary Information [file 41536_2021_138_MOESM1_ESM.pdf]

iPS cells thawing						
	Day and Duration	Materials in	Process Step	Materials Out	Location (Room and Grade)	Equipment
Step 1.1	Day -7<br>(15 minutes)	70% IPA	**Reagent spray**<br>Spray accurately all reagents before placing them in the BSC.		Tissue Culture Lab<br>(Grade B)	BSC
Step 1.2	Day -7<br>(5 minutes)	E8 basal media (1 x 500 mL)<br>E8 media supplement (10 mL)<br>Rock inhibitor 10 mM (1 x 591 µL vial)<br>Vitronectin (1 x 1mL vial)	**Reagent preparation**<br>Thaw E8 supplement overnight at 4°C. Take out media bottle from fridge and place at room temperature for 30 minutes. Add 10 mL supplement to new bottle of E8 basal media. Thaw one vial of Rock inhibitor at 4°C. Thaw one vial of vitronectin at room temperature.	E8 complete media (1 x 500 mL)<br>Rock inhibitor 10 mM (1 x 591 µL vial)<br>Vitronectin (1 x 1mL vial)	Tissue Culture Lab<br>(Grade B)	4-8˚C Fridge
Step 1.3	Day -7<br>(5 minutes)	Thawed vitronectin vial (1 x 1 mL)<br>PBS (1 x 500 mL)<br>10 mL stripette (x1)<br>P200 pipette tips (x1 box)<br>15 mL conical tube (x 1)	**Preparation of vitronectin-working solution**<br>Using a 10 mL stripette, transfer 6 mL PBS into a 15 mL conical tube. Using a p200 pipette, add 60 µL vitronectin into the PBS solution.	Vitronectin working solution (1 x 6 mL)<br>Waste	Tissue Culture Lab<br>(Grade B)	BSC<br>Pipette boy<br>P200 pipette
Step 1.4	Day -7<br>(60 min)	Vitronectin working solution (1 x 6 mL)<br>6-well plate (x1)<br>10 mL stripette (x1)	**Preparation of vitronectin-coated plates**<br>Using a 10 mL stripette, transfer 1 mL of vitronectin working solution into each well of a 6-well plate. Incubate at room temperature for 1 hour.	Vitronectin-coated 6-well plate (x1)<br>Waste	Tissue Culture Lab<br>(Grade B)	BSC<br>Pipette boy

iPS cells thawing

Step	Day and Duration	Materials in	Process Step	Materials Out	Location (Room and Grade)	Equipment
Step 1.5	Day -7 (10 minutes)	E8 Complete media (1 x 500 mL)<br>Rock inhibitor (1 x 591 µL)<br>50 mL stripette (x1)<br>P200 pipette tips (x 1 box)<br>50 mL conical tube	**Media preparation**<br>Using a 50 mL stripette, transfer 50 mL E8 Complete media to a 50 mL conical tube. Using a p200 pipette, add 50 µL Rock inhibitor.	E8 Complete media (450 mL)<br>Rock inhibitor (1 x 541 µL)<br>Supplemented E8 media (50 mL)<br>Waste	Tissue Culture Lab (Grade B)	BSC<br>Pipette boy<br>P200 pipette
Step 1.6	Day -7 (5 minutes)	iPS cells vial (x1)	**Cell retrieval**<br>Remove the vial from the liquid nitrogen and immediately place on dry ice.		Liquid Nitrogen storage room	Dry ice<br>Liquid Nitrogen vessel
Step 1.7	Day -7 (5 minutes)	iPS cells vial (x1)	**Cell thaw**<br>Partially submerge vial into a water bath at 37˚C. Inspect the vial till it is half or 2/3 thawed.	Thawed iPS cells vial (x1)	Tissue Culture Lab (Grade B)	Water bath
Step 1.8	Day -7 (5 minutes)	E8 Complete media 450 mL)<br>Thawed iPS cells vial (x1)<br>15 mL conical tube (x1)<br>P1000 pipette tops (1 box)<br>10 mL stripette (x1)	**Cell harvest**<br>Using a 10mL stripette, transfer 10mL E8 Complete media into a 15 mL conical tube. Using a p1000 pipette, transfer the thawed iPS cells (1 mL) to the 15 mL tube containing the media.	E8 Complete media (440 mL)<br>iPS cells in suspension (1 x 15 mL conical tube)<br>Waste	Tissue Culture Lab (Grade B)	BSC<br>Pipette boy<br>P1000 pipette

iPS cells thawing

Step	Day and Duration	Materials in	Process Step	Materials Out	Location (Room and Grade)	Equipment
Step 1.9	Day -7 (10 minutes)	iPS cells in suspension (1 x 15 mL conical tube)<br>Supplemented E8 media (50 mL)<br>Vitronectin-coated 6-well plate (x1)<br>P1000 pipette tips (x1 box)<br>Aspirator straw (x2)<br>5 mL stripette (x1)	**Cell seeding**<br>Centrifuge cells at 300g for 5 minutes. Using an aspirator, remove the media without touching the pellet. Using a p1000 pipette, resuspend cells into 1 mL of Supplemented E8 media with Rock inhibitor. Using an aspirator, remove the vitronectin from a vitronectin-coated 6-well plate. Using a 5 mL stripette, transfer 1 mL of supplemented E8 media to 2 wells of a vitronectin-coated 6-well plate. Using a p1000 pipette, transfer 500 µL of cell solution into each well.<br><br>Place the plate in the incubator set at 37˚C, 5% CO_2. Mix the culture by cross-shaped movements to distribute cells evenly.	iPS cells into plates (1 x 6-well plate)<br>Supplemented E8 media (45 mL)<br>Waste	Tissue Culture Lab (Grade B)	BSC<br>Aspirator<br>P1000 pipette<br>Incubator<br>Centrifuge<br>Pipet boy

Media exchange (D-6-D-2)

Step	Day and Duration	Materials in	Process Step	Materials Out	Location (Room and Grade)	Equipment
Step 1.10	Day -6/-2 (15 minutes)	70% IPA	**Reagent spray** Spray accurately all reagents before placing them in the BSC.		Tissue Culture Lab (Grade B)	BSC
Step 1.11	Day -6/-2 (10 minutes)	E8 basal media (5 x 500 mL)<br>E8 media supplement (5 x 10 mL)<br>10 mL stripette (x5)<br>50 mL stripette (x5)<br>50 mL conical tube (x5)	**Media preparation** Thaw E8 supplement overnight at 4°C. Add 10mL supplement to new bottle of E8 basal media. Using a 50 mL stripette, transfer 50 mL E8 Complete media to a 50 mL conical tube.	E8 Complete media (5 x 450 mL)<br>E8 Complete media aliquot (5 x 50 mL)<br>Waste	Tissue Culture Lab (Grade B)	4-8°C Fridge<br>BSC<br>Pipette boy
Step 1.12	Day -6/-2 (5 minutes)	iPS cells in a 6-well plate (x 2 wells)<br>E8 Complete media aliquot (50 mL)<br>Aspirator straw (x5)<br>5 mL stripette (x5)	**Media exchange** Using an aspirator, carefully remove the media by tilting the plate. Using a 5 mL stripette and tilting the plate, add 1.5 mL E8 complete media to the edge of each well.<br><br>Place the flask in the incubator set at 37˚C, 5% CO_2.	iPS cells in a 6-well plate (x2 wells)<br>Waste	Tissue Culture Lab (Grade B)	BSC<br>Incubator<br>Aspirator<br>Pipette boy

Pre-transfection seeding

Step	Day and Duration	Materials in	Process Step	Materials Out	Location (Room and Grade)	Equipment
Step 1.13	Day -1<br>(15 minutes)	70% IPA	**Reagent spray**<br>Spray accurately all reagents before placing them in the BSC.		Tissue Culture Lab (Grade B)	BSC
Step 1.14	Day -1<br>(5 minutes)	E8 basal media (1 x 500 mL)<br>E8 media supplement (10 mL)<br>Rock inhibitor 10 mM (1 x 591 µL vial)<br>Vitronectin (1 x 1mL vial)	**Reagent preparation**<br>Thaw E8 supplement overnight at 4°C. Take out media bottle from fridge and place at room temperature for 30 minutes. Add 10 mL supplement to new bottle of E8 basal media. Thaw one vial of Rock inhibitor at 4°C. Thaw one vial of vitronectin at room temperature.	E8 complete media (1 x 500 mL)<br>Rock inhibitor 10 mM (1 x 591 µL vial)<br>Vitronectin (1 x 1mL vial)	Tissue Culture Lab (Grade B)	4-8 °C Fridge
Step 1.15	Day -1<br>(5 minutes)	Thawed vitronectin vial (1 x 1 mL)<br>PBS (1 x 500 mL)<br>10 mL stripette (x1)<br>P200 pipette tips (x 1 box)<br>15 mL conical tube (x 1)	**Preparation of vitronectin-working solution**<br>Using a 10 mL stripette, transfer 6 mL PBS into a 15 mL conical tube. Using a p200 pipette, add 60 µL vitronectin into the PBS solution.	Vitronectin working solution (1 x 6 mL)<br>Waste	Tissue Culture Lab (Grade B)	BSC<br>Pipette boy<br>P200 pipette
Step 1.16	Day -1<br>(60 min)	Vitronectin working solution (1 x 6 mL)<br>12-well plate (x1)<br>P1000 pipette tips (x 1 box)	**Preparation of vitronectin-coated plates**<br>Using a p1000 pipette, transfer 500 µL of vitronectin working solution into each well of a 12-well plate. Incubate at room temperature for 1 hour.	Vitronectin-coated 12-well plate (x1)<br>Waste	Tissue Culture Lab (Grade B)	BSC<br>P1000 pipette

Pre-transfection seeding

Step	Day and Duration	Materials in	Process Step	Materials Out	Location (Room and Grade)	Equipment
Step 1.17	Day -1 (10 minutes)	E8 complete media (1 x 500 mL)<br>Rock inhibitor aliquot 10 mM (1 x 591 μL)<br>25 mL Stripette (x1)<br>P200 pipette tips (1 box)<br>50 mL conical tube (x1)	**Media preparation**<br>With a 25 mL stripette, transfer 20 mL E8 media into a 50 mL conical tube. With a P200 pipette, add 20 μL Rock inhibitor.	E8 complete media (480 mL)<br>Rock inhibitor aliquot 10 mM (1 x 571 μL)<br>Supplemented E8 media (20 mL)<br>Waste	Tissue Culture Lab (Grade B)	BSC<br>Pipette boy<br>P200 pipette
Step 1.18	Day -1 (5 minutes)	iPS cells in a 6-well plate (x 2 well)<br>PBS (500 mL)<br>Aspirator straw (x1)<br>P1000 pipette tips (1 box)	**Cell wash**<br>Using an aspirator, carefully remove the media by tilting the plate.<br>Using a p1000 pipette and tilting the plate, add 1 mL PBS.	PBS (498 mL)<br>Waste	Tissue Culture Lab (Grade B)	BSC<br>Aspirator<br>P1000 pipette
Step 1.19	Day -1 (10 minutes)	iPS cells in a 6-well plate (x 2 well)<br>TrypLE (1 x 100 mL)<br>Aspirator straw (x1)<br>P1000 pipette tips (1 box)	**Cell harvest**<br>Using an aspirator, carefully remove the PBS by tilting the plate.<br>Using a p1000 pipette, add 1 mL of TrypLE to each well.<br>Incubate for 3 minutes into the incubator.	TrypLE (98 mL)<br>iPS cells in a 6-well plate (x 2 well)<br>Waste	Tissue Culture Lab (Grade B)	BSC<br>Aspirator<br>P1000 pipette<br>Incubator
Step 1.20	Day -1 (5 minutes)		Visual inspection at the microscope<br>Are cells rounded and loosely attached to the plate?<br>NO: Incubate for 2 minutes in the incubator. (return to visual inspection)<br>YES: continue		Tissue Culture Lab (Grade B)	BSC<br>Microscope

Pre-transfection seeding

Step	Day and Duration	Materials in	Process Step	Materials Out	Location (Room and Grade)	Equipment
Step 1.21	Day -1 (10 minutes)	iPS cells in a 6-well plate (x 2 well)<br>E8 complete media (480 mL)<br>Supplemented E8 media (20 mL)<br>Trypan Blue (1 mL)<br>P10 pipette tips (x 1 box)<br>15 mL conical tube (x1)<br>5 mL stripette (x1)<br>1.5 mL Eppendorf tube (x1)<br>Aspirator straw (x1)<br>P1000 pipette tips (x 1 box)	**Cell harvest**<br>Carefully tilt the plate and remove the TrypLE by aspiration. Using a 5 mL stripette, transfer 5 mL E8 media into a 15 mL conical tube. Using a p1000 pipette, add 1 mL E8 media and pipette on the surface until cells dissociate from each well. Transfer cells into the 15 mL conical tube containing the media. Centrifuge 300g for 5 minutes and resuspend into 1 mL E8 media with Rock inhibitor. Using a p10 pipette, transfer a 10 µL cell aliquot for counting into a 1.5 mL Eppendorf tube.	iPS cells in suspension (1 mL)<br>iPS cells aliquot for counting (10 µL)<br>E8 complete media (473 mL)<br>Supplemented E8 media (19 mL)<br>Waste	Tissue Culture Lab (Grade B)	Aspirator<br>Pipette boy<br>P10 pipette<br>P1000 pipette<br>Centrifuge
Step 1.22	Day -1 (10 minutes)	iPS cells aliquot for counting (10 µL)<br>Trypan Blue (1 mL)<br>P10 pipette tips (x 1 box)	**Cell counting**<br>Combine the 10 µL cell aliquot with 10 µL Trypan Blue. Count cells using an haemocytometer	Total Cell Number N_{Cell}<br>Waste	Tissue Culture Lab (Grade B)	Haemacytometer<br>Microscope<br>P10 pipette

Pre-transfection seeding

Step	Day and Duration	Materials in	Process Step	Materials Out	Location (Room and Grade)	Equipment
Step 1.23	Day -1 (10 minutes)	Total Cell number N_{cell}	**Calculation of the required volume of cells to be seeded**<br>Based on the cell number, measured with the haemocytometer, calculate the volume of cells to be plated, in order to seed 1E05 cells into each well of a 12-well plate:<br><br>Vol_{cells} (µL)= (100,000 x 1000 / N_{cell})<br><br>Calculate the required amount of E8 media to add, to have a final concentration of 200,000 cells/mL and a final volume of 500 µL:<br><br>$Vol_{E8media}$ (µL)= (500-Vol_{cell})	Cell Volume to be seeded Vol_{cell}<br><br>E8 media Volume $Vol_{E8media}$		Calculator
Step 1.24	Day -1 (10 minutes)	iPS cells in suspension (1 mL)<br><br>Supplemented E8 media (19 mL)<br><br>Vitronectin-coated 12-well plate (x1)<br><br>P1000 pipette tips (1 box)<br><br>P200 pipette tips (1 box)<br><br>Aspirator straw (x1)<br><br>Cell Volume to be seeded Vol_{cell}<br><br>E8 media Volume $Vol_{E8media}$	**Cell seeding**<br>Using an aspirator, remove the vitronectin from each well of a vitronectin-coated 12-well plate. Using a p1000 pipette, add the required volume of E8 media ($Vol_{E8media}$) to each well. In this study 4 wells will be seeded (one control and 3 transfection replicates).<br>Using a p200 pipette, add the required volume of cells (Vol_{cells}) to each well containing the media.<br><br>Place the plate in the incubator set at 37˚C, 5% CO_2. Mix the culture by cross-shaped movements to distribute cells evenly.	Plated iPS cells in 12-well plate (x4 wells)<br><br>Waste	Tissue Culture Lab (Grade B)	BSC<br><br>Incubator<br><br>P1000 pipette<br><br>P200 pipette<br><br>Aspirator

Transfection						
	Day and Duration	Materials in	Process Step	Materials Out	Location (Room and Grade)	Equipment
Step 1.25	Day 0 (15 minutes)	70% IPA	**Reagent spray** Spray accurately all reagents before placing them in the BSC.		Tissue Culture Lab (Grade B)	BSC
Step 1.26	Day 0 (15 minutes)	E8 basal media (1 x 500 mL)<br>E8 media supplement (10 mL)<br>10 mL stripette (x5)<br>50 mL stripette (x5)<br>50 mL conical tube (x5)	**Media preparation** Thaw E8 supplement overnight at 4°C. Add 10mL supplement to new bottle of E8 basal media. Using a 50 mL stripette, transfer 50 mL E8 Complete media to a 50 mL conical tube.	E8 Complete media (450 mL)<br>E8 Complete media aliquot (50 mL)<br>Waste	Tissue Culture Lab (Grade B)	4-8°C Fridge<br>BSC<br>Pipette boy
Step 1.27	Day 0 (10 minutes)	sgRNA 100µM<br>Cas9 protein (61 µM)	**Reagent thaw** Take an aliquot of sgRNA, Cas9 protein and DNA plasmid and let them thaw in the fridge for 10 minutes.	Thawed sgRNA 100 µM<br>Thawed Cas9 protein (61 µM)	Tissue Culture Lab (Grade B)	BSC<br>4-8°C Fridge
Step 1.28	Day 0 (10 minutes)	Thawed sgRNA 100 µM<br>TE buffer (1 x 100 mL)<br>P200 pipette tips (1 box)<br>P10 pipette tips (1 box)<br>0.5 mL Eppendorf tube (x1)	**sgRNA preparation** Using a p200 pipette, transfer 32.3 µL TE Buffer to a 0.5 mL Eppendorf tube. Using a p10 pipette, add 1 µL sgRNA. Keep on ice.	sgRNA working stock (3 µM) (1 x 33.3 µL)<br>Waste	Tissue Culture Lab (Grade B)	BSC<br>Ice<br>P200 pipette<br>P10 pipette
Step 1.29	Day 0 (10 minutes)	E8 Complete media aliquot (50 mL)<br>Thawed Cas9 protein (61 µM)<br>P200 pipette tips (1 box)<br>P10 pipette tips (1 box)<br>0.5 mL Eppendorf tube (x1)	**Cas9 protein preparation** Using a p200 pipette, transfer 19.3 µL E8 complete media to a 0.5 mL Eppendorf tube. Using a p10 pipette, add 1 µL Cas9 protein. Keep on ice.	Cas9 working stock (3 µM) (1 x 20.3 µL)<br>Waste	Tissue Culture Lab (Grade B)	BSC<br>Ice<br>P200 pipette<br>P10 pipette

Transfection

Step	Day and Duration	Materials in	Process Step	Materials Out	Location (Room and Grade)	Equipment
Step 1.30	Day 0 (30 minutes)	sgRNA working stock (3 µM) (1 x 33.3 µL)<br>Cas9 working stock (3 µM) (1 x 20.3 µL)<br>E8 Complete media aliquot (50 mL)<br>Lipofectamine Stem cell (1 x 100 µL)<br>P200 pipette tips (1 box)<br>P10 pipette tips (1 box)<br>P20 pipette tips (1 box)<br>1.5 mL Eppendorf tube (x1)	**Transfection mix preparation**<br>Using a p200 pipette, transfer 150 µL E8 media to a 1.5 mL eppendorf tube at room temperature. In order, using a p10 pipette, add 7.8 µL sgRNA, 6.6 µL Cas9 protein. Using a p20 pipette add 15 µL Lipofectamine Stem Cell. Flick the tube and incubate at room temperature for 10-15 minutes.	Lipofectamine Stem cell (85 µL)<br>Transfection mix (180 µL)<br>Waste	Tissue Culture Lab (Grade B)	BSC<br>P200 pipette<br>P20 pipette<br>P10 pipette
Step 1.31	Day 0 (5 minutes)	Transfection mix (59.8 µL)<br>Plated iPS cells in 12-well plate (x1)<br>P200 pipette tips (1 box)	**Cell transfection**<br>Without pipetting, using a p200 pipette, add 60 µL of the solution dropwise to 3 wells of the 12-well plate containing the cells. Move the plate with cross-shape movements while adding the solution.<br><br>Place the plate in the incubator set at 37˚C, 5% CO_2.	Transfected iPS cells in 12-well plate (x1)<br>Waste	Tissue Culture Lab (Grade B)	BSC<br>P200 pipette<br>Incubator

Day 1-8: Media exchange

Step	Day and Duration	Materials in	Process Step	Materials Out	Location (Room and Grade)	Equipment
Step 1.32	Day 1-8 (15 minutes)	70% IPA	**Reagent spray** Spray accurately all reagents before placing them in the BSC.		Tissue Culture Lab (Grade B)	BSC
Step 1.33	Day 1-8 (10 minutes)	E8 basal media (8 x 500 mL)<br>E8 media supplement (8 x 10 mL)<br>10 mL stripette (x8)<br>50 mL stripette (x8)<br>50 mL conical tube (x8)	**Media preparation** Thaw E8 supplement overnight at 4°C. Add 10mL supplement to new bottle of E8 basal media. Using a 50 mL stripette, transfer 50 mL E8 Complete media to a 50 mL conical tube.	E8 Complete media (8 x 450 mL)<br>E8 Complete media aliquot (8 x 50 mL)<br>Waste	Tissue Culture Lab (Grade B)	4-8°C Fridge<br>BSC<br>Pipette boy
Step 1.34	Day 1-8 (5 minutes)	iPS cells in a 12-well plate (x 4 wells)<br>PBS (8 x 500 mL)<br>Aspirator straw (x8)<br>P1000 pipette tips (8 x 1 box)	**Cell wash** Using an aspirator, carefully remove the media by tilting the plate. Using a p1000 pipette and tilting the plate, add 1 mL PBS to each well.	iPS cells in a 12-well plate (x 4 wells)<br>PBS (8 x 496 mL)<br>Waste	Tissue Culture Lab (Grade B)	BSC<br>Aspirator<br>P1000 pipette
Step 1.35	Day 1-8 (5 minutes)	iPS cells in a 12-well plate (x 4 wells)<br>E8 Complete media aliquot (8 x 50 mL)<br>Aspirator straw (x8)<br>P1000 pipette (8 x 1 box)	**Media exchange** Using an aspirator, carefully remove PBS by tilting the plate. Using a p1000 pipette and tilting the plate, add 1 mL E8 complete media slowly to the edge of each well.<br><br>Place the flask in the incubator set at 37˚C, 5% CO2.	iPS cells in a 12-well plate (x 4 wells)<br>Waste	Tissue Culture Lab (Grade B)	BSC<br>Incubator<br>Aspirator<br>P1000 pipette

Day 9: Collection of individual colonies

Step	Day and Duration	Materials in	Process Step	Materials Out	Location (Room and Grade)	Equipment
Step 1.36	Day 9 (15 minutes)	70% IPA	**Reagent spray** Spray accurately all reagents before placing them in the BSC.		Tissue Culture Lab (Grade B)	BSC
Step 1.37	Day 9 (5 minutes)	E8 basal media (1 x 500 mL); E8 media supplement (10 mL); Rock inhibitor 10 mM (1 x 591 µL vial); Vitronectin (1 x 1mL vial)	**Reagent preparation** Thaw E8 supplement overnight at 4°C. Take out media bottle from fridge and place at room temperature for 30 minutes. Add 10 mL supplement to new bottle of E8 basal media. Thaw one vial of Rock inhibitor at 4°C. Thaw one vial of vitronectin at room temperature.	E8 complete media (1 x 500 mL); Rock inhibitor 10 mM (1 x 591 µL vial); Vitronectin (1 x 1mL vial)	Tissue Culture Lab (Grade B)	4-8˚C Fridge
Step 1.38	Day 9 (5 minutes)	Thawed vitronectin vial (1 x 1 mL); PBS (1 x 500 mL); 25 mL stripette (x1); P200 pipette tips (x 1 box); 50 mL conical tube (x 1)	**Preparation of vitronectin-working solution** Using a 25 mL stripette, transfer 20 mL PBS into a 50 mL conical tube. Using a p200 pipette, add 200 µL vitronectin into the PBS solution.	Vitronectin working solution (1 x 20 mL); Waste	Tissue Culture Lab (Grade B)	BSC; Pipette boy; P200 pipette
Step 1.39	Day 9 (10 minutes)	Vitronectin working solution (1 x 20 mL); 12-well plate (x3); P1000 pipette tips (x 1 box)	**Preparation of vitronectin-coated plates** Using a p1000 pipette, transfer 500 µL of vitronectin working solution into each well of 3 x 12-well plate. Incubate at room temperature for 1 hour.	Vitronectin-coated 12-well plate (x3); Waste	Tissue Culture Lab (Grade B)	BSC; P1000 pipette

Day 9: Collection of individual colonies

Step	Day and Duration	Materials in	Process Step	Materials Out	Location (Room and Grade)	Equipment
Step 1.40	Day 9 (10 minutes)	E8 complete media (1 x 500 mL)<br>Rock inhibitor 10 mM (1 x 591 µL)<br>50 mL Stripette (x1)<br>P200 pipette tips (1 box)<br>50 mL conical tube (x1)	**Media preparation**<br>With a 50 mL stripette, transfer 50 mL E8 complete media into a 50 mL conical tube. With a P200 pipette, add 50 µL Rock inhibitor.	E8 complete media (450 mL)<br>Rock inhibitor 10 mM (1 x 541 µL)<br>Supplemented E8 media (50 mL)<br>Waste	Tissue Culture Lab (Grade B)	BSC<br>P200 pipette<br>Pipette boy
Step 1.41	Day 9 (5 minutes)	iPS cells in a 12-well plate (x 4 wells)<br>PBS (500 mL)<br>Aspirator straw (x1)<br>P1000 pipette tips (1 box)	**Cell wash**<br>Using an aspirator, carefully remove the media by tilting the plate. Using a p1000 pipette and tilting the plate, add 1 mL PBS. Using an aspirator, remove the PBS.	PBS (496 mL)<br>Waste	Tissue Culture Lab (Grade B)	BSC<br>Aspirator<br>P1000 pipette
Step 1.42	Day 9 (30 minutes)	iPS cells in a 12-well plate (x1 well)<br>Vitronectin-coated 12-well plates (x3)<br>Supplemented E8 media (50 mL)<br>P200 pipette tips (1 box)<br>10 mL stripette	**Seeding of individual colonies**<br>Using an aspirator, remove the vitronectin from the vitronectin-coated 12-well plates. Using a 10 mL stripette, transfer 1 mL E8 media into each well. Using a p200 pipette and 100 µL media, pick each individual colony and transfer it to a single well of the 12 well plates containing the media. Pipette to break down the colonies into single cells. Repeat this passage for 10 individual colonies for each edited well (total 30 colonies).<br><br>Place the plate in the incubator set at 37˚C, 5% CO_2.	iPS cells in 12-well plates (x3 plates)<br>Waste	Tissue Culture Lab (Grade B)	BSC<br>Aspirator<br>P200 pipette<br>Microscope<br>Pipette boy

Day 10-15: Media exchange

Step	Day and Duration	Materials in	Process Step	Materials Out	Location (Room and Grade)	Equipment
Step 1.43	Day 10-15 (15 minutes)	70% IPA	**Reagent spray** Spray accurately all reagents before placing them in the BSC.		Tissue Culture Lab (Grade B)	BSC
Step 1.44	Day 10-15 (10 minutes)	E8 basal media (6 x 500 mL)<br>E8 media supplement (6 x 10 mL)<br>10 mL stripette (x6)<br>50 mL stripette (x6)<br>50 mL conical tube (x6)	**Media preparation** Thaw E8 supplement overnight at 4°C. Add 10mL supplement to new bottle of E8 basal media. Using a 50 mL stripette, transfer 50 mL E8 Complete media to a 50 mL conical tube.	E8 Complete media (6 x 450 mL)<br>E8 Complete media aliquot (6 x 50 mL)<br>Waste	Tissue Culture Lab (Grade B)	4-8°C Fridge<br>BSC<br>Pipette boy
Step 1.45	Day 10-15 (10 minutes)	iPS cells in a 12-well plate (x 3 plates)<br>PBS (6 x 500 mL)<br>Aspirator straw (x6)<br>10 mL stripette (x18)	**Cell wash** Using an aspirator, carefully remove the media by tilting the plate. Using a 10 mL stripette and tilting the plate, add 1 mL PBS to each well.	PBS (6 x 470 mL)<br>iPS cells in a 12-well plate (x 3 plates)<br>Waste	Tissue Culture Lab (Grade B)	BSC<br>Aspirator<br>Pipette boy
Step 1.46	Day 10-15 (5 minutes)	iPS cells in a 12-well plate (x 3 plates)<br>E8 Complete media aliquot (6 x 50 mL)<br>Aspirator straw (x6)<br>10 mL stripette (x18)	**Media exchange** Using an aspirator, carefully remove PBS by tilting the plate. Using a 10 mL stripette and tilting the plate, add 1 mL E8 complete media slowly to the edge of each well.<br><br>Place the flask in the incubator set at 37˚C, 5% CO_2.	iPS cells in a 12-well plate (x 3 plates)<br>Waste	Tissue Culture Lab (Grade B)	BSC<br>Incubator<br>Aspirator<br>P1000 pipette

Day 16: Homozygous clone selection

Step	Day and Duration	Materials in	Process Step	Materials Out	Location (Room and Grade)	Equipment
Step 1.47	Day 16 (15 minutes)	70% IPA	**Reagent spray** Spray accurately all reagents before placing them in the BSC.		Tissue Culture Lab (Grade B)	BSC
Step 1.48	Day 16 (5 minutes)	E8 basal media (1 x 500 mL); E8 media supplement (10 mL); 10 mL stripette (x1); 50 mL stripette (x1); 50 mL conical tube (x1)	**Media preparation** Thaw E8 supplement overnight at 4°C. Add 10mL supplement to new bottle of E8 basal media. Using a 50 mL stripette, transfer all 50 mL E8 Complete media to a 50 mL conical tube.	E8 Complete media (450 mL); E8 Complete media aliquot (50 mL); Waste	Tissue Culture Lab (Grade B)	4-8°C Fridge; BSC; Pipette boy
Step 1.49	Day 16 (5 minutes)	KOSR (1 x 100 mL); DMSO (1 x 5 mL); 25 mL stripettes (x1); 5 mL stripettes (x1); 50 mL conical tube (x1)	**Freezing media B preparation** Using a 25 mL stripette, transfer 16 mL KOSR into a 50 mL conical tube. Using a 5 mL stripette, add 4 mL DMSO.	KOSR (1 x 84 mL); Freezing media B (1 x 20 mL); Waste	Tissue Culture Lab (Grade B)	BSC; Pipette boy
Step 1.50	Day 16 (10 minutes)	Plated iPS cells into 12-well plates (x 3 plates); PBS (1 x 500 mL); Aspirator straw (x1); 10 mL stripette (x3)	**Cell wash** Using an aspirator, carefully remove the media by tilting the flask. Using a 10 mL stripette and tilting the flask, add 1 mL PBS to each well.	PBS (1 x 470 mL); Waste	Tissue Culture Lab (Grade B)	BSC; Aspirator; Pipette boy
Step 1.51	Day 16 (10 minutes)	TrypLE (100 mL); Aspirator straw (x1); 10 mL stripette (x3)	**Cell harvest** Using an aspirator, carefully remove the PBS by tilting the flask. Using a 10 mL stripette, add 1 mL of TrypLE to each well. Incubate for 3 minutes in the incubator.	TrypLE (1 x 70 mL); Waste	Tissue Culture Lab (Grade B)	BSC; Aspirator; Pipette boy; Incubator

Day 16: Homozygous clone selection

Step	Day and Duration	Materials in	Process Step	Materials Out	Location (Room and Grade)	Equipment
Step 1.52	Day 16 (5 minutes)		Visual inspection at the microscope → Are cells rounded and loosely attached to the plate? NO → Incubate for 2 minutes in the incubator (return to visual inspection); YES → next step		Tissue Culture Lab (Grade B)	Microscope<br>Incubator
Step 1.53	Day 16 (15 minutes)	E8 complete media aliquot (1 x 50 mL)<br>Aspirator straw (x1)<br>P1000 pipette tips (x 1 box)<br>1.5 mL Eppendorf tube (x33)<br>P10 pipette tips (x 1 box)	**Cell harvest** Carefully tilt the plate and remove TrypLE from the well by aspiration. Using a p1000 pipette, add 1 mL E8 media on each well and pipette on the surface until cells dissociate from the plate. Using a p1000 pipette transfer the cell solution from each well to a 1.5 mL Eppendorf tube. Using a p10 pipette, transfer 10 µL cell solution from 3 different wells to 1.5 mL Eppendorf tubes.	iPS cells in suspension (30 x 1 mL)<br>iPS cells aliquots for counting (3 x 10 µL)<br>E8 complete media (20 mL)<br>Waste	Tissue Culture Lab (Grade B)	BSC<br>P1000 pipette<br>P10 pipette<br>Aspirator
Step 1.54	Day 16 (15 minutes)	iPS cells aliquots for counting (3 x 10 µL)<br>Trypan Blue (1 mL)<br>P10 pipette tips (x1 box)	**Cell counting** Using a p10 pipette, combine each aliquot of 10 µL cells with 10 µL Trypan Blue. Count cells using an haemocytometer	Total cell number N_{cell}<br>Waste	Tissue Culture Lab (Grade B)	Haemacytometer<br>P10 pipette<br>Microscope

Day 16: Homozygous clone selection

	Day and Duration	Materials in	Process Step	Materials Out	Location (Room and Grade)	Equipment
Step 1.55	Day 16<br>(5 minutes)	Total cell number N_{cell}	**Calculation of cell volume for banking**<br>Based on the average cell number, measured with the haemocytometer, calculate the volume of cell solution containing 1E06 cells (V_{cell}).	Volume containing 1E06 cells V_{cell}	Tissue Culture Lab<br>(Grade B)	Calculator
Step 1.56	Day 16<br>(15 minutes)	iPS cells in suspension (30 x 1 mL)<br>Volume containing 1E06 cells (V_{cell})<br>P1000 pipette tips (x 1 box)<br>1.5 mL Eppendorf tube (x30)	**Cell aliquoting**<br>Using a p1000 pipette, transfer V_{cell} for each vial to new 1.5 mL Eppendorf tubes. Centrifuge all the samples at 300g for 5 minutes.	iPS cells pellet for banking (x30)<br>iPS cells pellet for genotyping (x30)<br>Waste	Tissue Culture Lab<br>(Grade B)	BSC<br>P1000 pipette<br>Centrifuge
Step1.57	Day 16<br>(15 minutes)	iPS cells pellet for banking (x30)<br>KOSR<br>(1 x 84 mL)<br>Freezing media B<br>(20 mL)<br>P1000 pipette tips (x1 box)<br>Cryovial (x30)<br>Coolcell	**Cell resuspension into freezing media**<br>Gently flick each tube to fully dislodge the pellet from the tube bottom. Using a p1000 pipette resuspend the cells into 500 µL of KOSR (Freezing media A). Following uniform suspension, using a p1000 pipette, add 500 µL of Freezing media B in a drop-wise manner. Using a p1000 pipette, transfer 1 mL cell solution to each cryovial.<br><br>Quickly place the vial into a Coolcell and transfer to -80˚C overnight. Transfer the cells to a liquid nitrogen tank for long-term storage.	Frozen iPS cells (x30 cryovials)<br>Waste<br>Coolcell	Tissue Culture Lab<br>(Grade B)<br>Liquid nitrogen storage	BSC<br>P1000 pipette<br>Coolcell<br>Liquid nitrogen tank

Day 16: Homozygous clone selection						
	Day and Duration	Materials in	Process Step	Materials Out	Location (Room and Grade)	Equipment
Step 1.58		iPS cells pellet for genotyping (x30)	**Genotyping** Perform DNA extraction and sequencing to genotype each individual clone.	Genotyping data	Analytical Lab	PCR machine
Step 1.59		Genotyping data	Is clone homozygous for the editing? NO → Rejected Product; YES → Go to Step 2.67.	Rejected Product		

iPS cells thawing

Step	Day and Duration	Materials in	Process Step	Materials Out	Location (Room and Grade)	Equipment
Step 2.1	Day -7 (15 minutes)	70% IPA	**Reagent spray** Spray accurately all reagents before placing them in the BSC.		Tissue Culture Lab (Grade B)	BSC
Step 2.2	Day -7 (5 minutes)	E8 basal media (1 x 500 mL)<br>E8 media supplement (10 mL)<br>Rock inhibitor 10 mM (1 x 591 µL vial)<br>Vitronectin (1 x 1mL vial)	**Reagent preparation** Thaw E8 supplement overnight at 4°C. Take out media bottle from fridge and place at room temperature for 30 minutes. Add 10 mL supplement to new bottle of E8 basal media. Thaw one vial of Rock inhibitor at 4°C. Thaw one vial of vitronectin at room temperature.	E8 complete media (1 x 500 mL)<br>Rock inhibitor 10 mM (1 x 591 µL vial)<br>Vitronectin (1 x 1mL vial)	Tissue Culture Lab (Grade B)	4-8˚C Fridge
Step 2.3	Day -7 (5 minutes)	Thawed vitronectin vial (1 x 1 mL)<br>PBS (1 x 500 mL)<br>10 mL stripette (x1)<br>P200 pipette tips (x1 box)<br>15 mL conical tube (x 1)	**Preparation of vitronectin-working solution** Using a 10 mL stripette, transfer 6 mL PBS into a 15 mL conical tube. Using a p200 pipette, add 60 µL vitronectin into the PBS solution.	Vitronectin working solution (1 x 6 mL)<br>Waste	Tissue Culture Lab (Grade B)	BSC<br>Pipette boy<br>P200 pipette
Step 2.4	Day -7 (60 min)	Vitronectin working solution (1 x 6 mL)<br>6-well plate (x1)<br>10 mL stripette (x1)	**Preparation of vitronectin-coated plates** Using a 10 mL stripette, transfer 1 mL of vitronectin working solution into each well of a 6-well plate. Incubate at room temperature for 1 hour.	Vitronectin-coated 6-well plate (x1)<br>Waste	Tissue Culture Lab (Grade B)	BSC<br>Pipette boy

iPS cells thawing

Step	Day and Duration	Materials in	Process Step	Materials Out	Location (Room and Grade)	Equipment
Step 2.5	Day -7 (10 minutes)	E8 complete media (1 x 500 mL)<br>Rock inhibitor (1 x 591 µL)<br>50 mL stripette (x1)<br>P200 pipette tips (x 1 box)<br>50 mL conical tube	**Media preparation** Using a 50 mL stripette, transfer 50 mL E8 media to a 50 mL conical tube. Using a p200 pipette, add 50 µL Rock inhibitor.	E8 complete media (450 mL)<br>Rock inhibitor (1 x 541 µL)<br>Supplemented E8 media (50 mL)<br>Waste	Tissue Culture Lab (Grade B)	BSC<br>Pipette boy<br>P200 pipette
Step 2.6	Day -7 (5 minutes)	iPS cells vial (x1)	**Cell retrieval** Remove the vial from the liquid nitrogen and immediately place on dry ice.		Liquid Nitrogen storage room	Dry ice<br>Liquid Nitrogen vessel
Step 2.7	Day -7 (5 minutes)	iPS cells vial (x1)	**Cell thaw** Partially submerge vial into a water bath at 37˚C. Inspect the vial till it is half or 2/3 thawed.	Thawed iPS cells vial (x1)	Tissue Culture Lab (Grade B)	Water bath
Step 2.8	Day -7 (5 minutes)	E8 complete media (450 mL)<br>Thawed iPS cells vial (x1)<br>15 mL conical tube (x1)<br>P1000 pipette tops (1 box)<br>10 mL stripette (x1)	**Cell harvest** Using a 10mL stripette, transfer 10mL E8 complete media into a 15 mL conical tube. Using a p1000 pipette, transfer the thawed iPS cells (1 mL) to the 15 mL tube containing the media.	E8 complete media (440 mL)<br>iPS cells in suspension (1 x 15 mL conical tube)<br>Waste	Tissue Culture Lab (Grade B)	BSC<br>Pipette boy<br>P1000 pipette

iPS cells thawing

	Day and Duration	Materials in	Process Step	Materials Out	Location (Room and Grade)	Equipment
Step 2.9	Day -7 (10 minutes)	iPS cells in suspension (1 x 15 mL conical tube)<br>Supplemented E8 media (50 mL)<br>Vitronectin-coated 6-well plate (x1)<br>P1000 pipette tips (x1 box)<br>Aspirator straw (x2)<br>5 mL stripette (x1)	**Cell seeding**<br>Centrifuge cells at 300g for 5 minutes. Using an aspirator, remove the media without touching the pellet. Using a p1000 pipette, resuspend cells into 1 mL of Supplemented E8 media with Rock inhibitor. Using an aspirator, remove the vitronectin from a vitronectin-coated 6-well plate. Using a 5 mL stripette, transfer 1 mL of supplemented E8 media to 2 wells of a vitronectin-coated 6-well plate. Using a p1000 pipette, transfer 500 µL of cell solution into each well.<br><br>Place the plate in the incubator set at 37˚C, 5% CO_2. Mix the culture by cross-shaped movements to distribute cells evenly.	iPS cells into plates (1 x 6-well plate)<br>Supplemented E8 media (45 mL)<br>Waste	Tissue Culture Lab (Grade B)	BSC<br>Aspirator<br>P1000 pipette<br>Incubator<br>Centrifuge<br>Pipet boy

Media exchange (D-6-D-2)

Step	Day and Duration	Materials in	Process Step	Materials Out	Location (Room and Grade)	Equipment
Step 2.10	Day -6/-2 (15 minutes)	70% IPA	**Reagent spray** Spray accurately all reagents before placing them in the BSC.		Tissue Culture Lab (Grade B)	BSC
Step 2.11	Day -6/-2 (10 minutes)	E8 basal media (5 x 500 mL)<br>E8 media supplement (5 x 10 mL)<br>10 mL stripette (x5)<br>50 mL stripette (x5)<br>50 mL conical tube (x5)	**Media preparation** Thaw E8 supplement overnight at 4°C. Add 10mL supplement to new bottle of E8 basal media. Using a 50 mL stripette, transfer 50 mL E8 Complete media to a 50 mL conical tube.	E8 Complete media (5 x 450 mL)<br>E8 Complete media aliquot (5 x 50 mL)<br>Waste	Tissue Culture Lab (Grade B)	4-8°C Fridge<br>BSC<br>Pipette boy
Step 2.12	Day -6/-2 (5 minutes)	iPS cells in a 6-well plate (x 2 wells)<br>E8 Complete media aliquot (5 x 50 mL)<br>Aspirator straw (x5)<br>5 mL stripette (x5)	**Media exchange** Using an aspirator, carefully remove the media by tilting the plate. Using a 5 mL stripette and tilting the plate, add 1.5 mL E8 complete media slowly to the edge of each well.<br><br>Place the flask in the incubator set at 37˚C, 5% CO2.	iPS cells in a 6-well plate (x2 wells)<br>Waste	Tissue Culture Lab (Grade B)	BSC<br>Incubator<br>Aspirator<br>Pipette boy

Pre-transfection seeding

Step	Day and Duration	Materials in	Process Step	Materials Out	Location (Room and Grade)	Equipment
Step 2.13	Day -1 (15 minutes)	70% IPA	**Reagent spray** Spray accurately all reagents before placing them in the BSC.		Tissue Culture Lab (Grade B)	BSC
Step 2.14	Day -1 (5 minutes)	E8 basal media (1 x 500 mL)<br>E8 media supplement (10 mL)<br>Rock inhibitor 10 mM (1 x 591 µL vial)<br>Vitronectin (1 x 1mL vial)	**Reagent preparation** Thaw E8 supplement overnight at 4°C. Take out media bottle from fridge and place at room temperature for 30 minutes. Add 10 mL supplement to new bottle of E8 basal media. Thaw one vial of Rock inhibitor at 4°C. Thaw one vial of vitronectin at room temperature.	E8 complete media (1 x 500 mL)<br>Rock inhibitor 10 mM (1 x 591 µL vial)<br>Vitronectin (1 x 1mL vial)	Tissue Culture Lab (Grade B)	4-8˚C Fridge
Step 2.15	Day -1 (5 minutes)	Thawed vitronectin vial (1 x 1 mL)<br>PBS (1 x 500 mL)<br>10 mL stripette (x1)<br>P200 pipette tips (x 1 box)<br>15 mL conical tube (x 1)	**Preparation of vitronectin-working solution** Using a 10 mL stripette, transfer 6 mL PBS into a 15 mL conical tube. Using a p200 pipette, add 60 µL vitronectin into the PBS solution.	Vitronectin working solution (1 x 6 mL)<br>Waste	Tissue Culture Lab (Grade B)	BSC<br>Pipette boy<br>P200 pipette
Step 2.16	Day -1 (60 min)	Vitronectin working solution (1 x 6 mL)<br>12-well plate (x1)<br>P1000 pipette tips (x 1 box)	**Preparation of vitronectin-coated plates** Using a p1000 pipette, transfer 500 µL of vitronectin working solution into each well of a 12-well plate. Incubate at room temperature for 1 hour.	Vitronectin-coated 12-well plate (x1)<br>Waste	Tissue Culture Lab (Grade B)	BSC<br>P1000 pipette

Pre-transfection seeding

Step	Day and Duration	Materials in	Process Step	Materials Out	Location (Room and Grade)	Equipment
Step 2.17	Day -1 (10 minutes)	E8 complete media (1 x 500 mL)<br>Rock inhibitor aliquot 10 mM (1 x 591 µL)<br>25 mL Stripette (x1)<br>P200 pipette tips (1 box)<br>50 mL conical tube (x1)	**Media preparation**<br>With a 25 mL stripette, transfer 20 mL E8 media into a 50 mL conical tube. With a P200 pipette, add 20 µL Rock inhibitor.	E8 complete media (480 mL)<br>Rock inhibitor aliquot 10 mM (1 x 571 µL)<br>Supplemented E8 media (20 mL)<br>Waste	Tissue Culture Lab (Grade B)	BSC<br>Pipette boy<br>P200 pipette
Step 2.18	Day -1 (5 minutes)	iPS cells in a 6-well plate (x 2 well)<br>PBS (500 mL)<br>Aspirator straw (x1)<br>P1000 pipette tips (1 box)	**Cell wash**<br>Using an aspirator, carefully remove the media by tilting the plate.<br>Using a p1000 pipette and tilting the plate, add 1 mL PBS.	PBS (498 mL)<br>Waste	Tissue Culture Lab (Grade B)	BSC<br>Aspirator<br>P1000 pipette
Step 2.19	Day -1 (10 minutes)	iPS cells in a 6-well plate (x 2 well)<br>TrypLE (1 x 100 mL)<br>Aspirator straw (x1)<br>P1000 pipette tips (1 box)	**Cell harvest**<br>Using an aspirator, carefully remove the PBS by tilting the plate.<br>Using a p1000 pipette, add 1 mL of TrypLE to each well.<br>Incubate for 3 minutes into the incubator.	TrypLE (98 mL)<br>iPS cells in a 6-well plate (x 2 well)<br>Waste	Tissue Culture Lab (Grade B)	BSC<br>Aspirator<br>P1000 pipette<br>Incubator
Step 2.20	Day -1 (5 minutes)		Visual inspection at the microscope<br>Are cells rounded and loosely attached to the plate?<br>NO → Incubate for 2 minutes in the incubator. (return to visual inspection)<br>YES → next step		Tissue Culture Lab (Grade B)	BSC<br>Microscope

Pre-transfection seeding

Step	Day and Duration	Materials in	Process Step	Materials Out	Location (Room and Grade)	Equipment
Step 2.21	Day -1 (10 minutes)	iPS cells in a 6-well plate (x 2 well)<br>E8 complete media (480 mL)<br>Supplemented E8 media (20 mL)<br>Trypan Blue (1 mL)<br>P10 pipette tips (x 1 box)<br>15 mL conical tube (x1)<br>5 mL stripette (x1)<br>1.5 mL Eppendorf tube (x1)<br>Aspirator straw (x1)<br>P1000 pipette tips (x 1 box)	**Cell harvest**<br>Carefully tilt the plate and remove the TrypLE by aspiration. Using a 5 mL stripette, transfer 5 mL E8 complete media into a 15 mL conical tube. Using a p1000 pipette, add 1 mL E8 complete media and pipette on the surface until cells dissociate from each well. Transfer cells into the 15 mL conical tube containing the media. Centrifuge 300g for 5 minutes and resuspend into 1 mL supplemented E8 media with Rock inhibitor. Using a p10 pipette, transfer a 10 µL cell aliquot for counting into a 1.5 mL Eppendorf tube.	iPS cells in suspension (1 mL)<br>iPS cells aliquot for counting (10 µL)<br>E8 complete media (473 mL)<br>Supplemented E8 media (19 mL)<br>Waste	Tissue Culture Lab (Grade B)	Aspirator<br>Pipette boy<br>P10 pipette<br>P1000 pipette<br>Centrifuge
Step 2.22	Day -1 (10 minutes)	iPS cells aliquot for counting (10 µL)<br>Trypan Blue (1 mL)<br>P10 pipette tips (x 1 box)	**Cell counting**<br>Combine the 10 µL cell aliquot with 10 µL Trypan Blue. Count cells using an haemocytometer	Total Cell Number N_{Cell}<br>Waste	Tissue Culture Lab (Grade B)	Haemacytometer<br>Microscope<br>P10 pipette

Pre-transfection seeding

	Day and Duration	Materials in	Process Step	Materials Out	Location (Room and Grade)	Equipment
Step 2.23	Day -1 (10 minutes)	Total Cell number N_{cell}	**Calculation of the required volume of cells to be seeded**<br>Based on the cell number, measured with the haemocytometer, calculate the volume of cells to be plated, in order to seed 1E05 cells into each well of a 12-well plate:<br><br>Vol_{cells} (µL)= (100,000 x 1000 / N_{cell})<br><br>Calculate the required amount of E8 media to add, to have a final concentration of 200,000 cells/mL and a final volume of 500 µL:<br><br>$Vol_{E8media}$ (µL)= (500-Vol_{cell})	Cell Volume to be seeded Vol_{cell}<br><br>E8 media Volume $Vol_{E8media}$		Calculator
Step 2.24	Day -1 (10 minutes)	iPS cells in suspension (1 mL)<br><br>Supplemented E8 media (19 mL)<br><br>Vitronectin-coated 12-well plate (x1)<br><br>P1000 pipette tips (1 box)<br><br>P200 pipette tips (1 box)<br><br>Aspirator straw (x1)<br><br>Cell Volume to be seeded Vol_{cell}<br><br>E8 media Volume $Vol_{E8media}$	**Cell seeding**<br>Using an aspirator, remove the vitronectin from each well of a vitronectin-coated 12-well plate. Using a p1000 pipette, add the required volume of E8 media ($Vol_{E8media}$) to each well. In this study 4 wells will be seeded (one control and 3 transfection replicates).<br>Using a p200 pipette, add the required volume of cells (Vol_{cells}) to each well containing the media.<br><br>Place the plate in the incubator set at 37˚C, 5% CO2. Mix the culture by cross-shaped movements to distribute cells evenly.	Plated iPS cells in 12-well plate (x4 wells)<br><br>Waste	Tissue Culture Lab (Grade B)	BSC<br><br>Incubator<br><br>P1000 pipette<br><br>P200 pipette<br><br>Aspirator

Transfection						
	Day and Duration	Materials in	Process Step	Materials Out	Location (Room and Grade)	Equipment
Step 2.25	Day 0 (15 minutes)	70% IPA	**Reagent spray** Spray accurately all reagents before placing them in the BSC.		Tissue Culture Lab (Grade B)	BSC
Step 2.26	Day 0 (15 minutes)	E8 basal media (1 x 500 mL); E8 media supplement (10 mL); 10 mL stripette (x5); 50 mL stripette (x5); 50 mL conical tube (x5)	**Media preparation** Thaw E8 supplement overnight at 4°C. Add 10mL supplement to new bottle of E8 basal media. Using a 50 mL stripette, transfer 50 mL E8 Complete media to a 50 mL conical tube.	E8 Complete media (450 mL); E8 Complete media aliquot (50 mL); Waste	Tissue Culture Lab (Grade B)	4-8°C Fridge; BSC; Pipette boy
Step 2.27	Day 0 (10 minutes)	sgRNA 100µM; Cas9 protein (61 µM); DNA plasmid	**Reagent thaw** Take a vial of sgRNA, Cas9 protein and DNA plasmid and let them thaw in the fridge for 10 minutes.	Thawed sgRNA 100 µM; Thawed Cas9 protein (61 µM); Thawed DNA plasmid	Tissue Culture Lab (Grade B)	BSC; 4-8°C Fridge
Step 2.28	Day 0 (10 minutes)	Thawed sgRNA 100 µM; TE buffer (1 x 100 mL); P200 pipette tips (1 box); P10 pipette tips (1 box); 0.5 mL Eppendorf tube (x1)	**sgRNA preparation** Using a p200 pipette, transfer 32.3 µL TE Buffer to a 0.5 mL Eppendorf tube. Using a p10 pipette, add 1 µL sgRNA. Keep on ice.	sgRNA working stock (3 µM) (1 x 33.3 µL); Waste	Tissue Culture Lab (Grade B)	BSC; Ice; P200 pipette; P10 pipette
Step 2.29	Day 0 (10 minutes)	E8 Complete media aliquot (50 mL); Thawed Cas9 protein (61 µM); P200 pipette tips (1 box); P10 pipette tips (1 box); 0.5 mL Eppendorf tube (x1)	**Cas9 protein preparation** Using a p200 pipette, transfer 19.3 µL E8 complete media to a 0.5 mL Eppendorf tube. Using a p10 pipette, add 1 µL Cas9 protein. Keep on ice.	Cas9 working stock (3 µM) (1 x 20.3 µL); Waste	Tissue Culture Lab (Grade B)	BSC; Ice; P200 pipette; P10 pipette

Transfection

	Day and Duration	Materials in	Process Step	Materials Out	Location (Room and Grade)	Equipment
Step 2.30	Day0 (10 minutes)	Thawed DNA aliquot (1 x 2 µL)<br>TE buffer (1 x 100 mL)<br>P200 pipette tips (1 box)<br>P10 pipette tips (1 box)<br>0.5 mL Eppendorf tube (x1)	**DNA preparation**	DNA working stock (1 x 33.3 µL)<br>Waste	Tissue Culture Lab (Grade B)	BSC<br>Ice<br>P200 pipette<br>P10 pipette
Step 2.31	Day 0 (30 minutes)	sgRNA working stock (3 µM) (1 x 33.3 µL)<br>Cas9 working stock (3 µM) (1 x 20.3 µL)<br>DNA working stock (1 x 33.3 µL)<br>E8 Complete media aliquot (50 mL)<br>Lipofectamine Stem cell (1 x 100 µL)<br>P200 pipette tips (1 box)<br>P10 pipette tips (1 box)<br>P20 pipette tips (1 box)<br>1.5 mL Eppendorf tube (x1)	**Transfection mix preparation**<br>Using a p200 pipette, transfer 150 µL E8 complete media to a 1.5 mL eppendorf tube at room temperature. In order, using a p10 pipette, add 7.8 µL sgRNA, 6.6 µL Cas9 protein (and DNA?). Using a p20 pipette add 15 µL Lipofectamine Stem Cell. Flick the tube and incubate at room temperature for 10-15 minutes.	Lipofectamine Stem cell (85 µL)<br>Transfection mix (180 µL)<br>Waste	Tissue Culture Lab (Grade B)	BSC<br>P200 pipette<br>P20 pipette<br>P10 pipette
Step 2.32	Day 0 (5 minutes)	Transfection mix (59.8 µL)<br>Plated iPS cells in 12-well plate (x1)<br>P200 pipette tips (1 box)	**Cell transfection**<br>Without pipetting, using a p200 pipette, add 60 µL of the solution dropwise to 3 wells of the 12-well plate containing the cells. Move the plate with cross-shape movements while adding the solution.<br><br>Place the plate in the incubator set at 37˚C, 5% CO_2.	Transfected iPS cells in 12-well plate (x1)<br>Waste	Tissue Culture Lab (Grade B)	BSC<br>P200 pipette<br>Incubator

Day 1: Media exchange

Step	Day and Duration	Materials in	Process Step	Materials Out	Location (Room and Grade)	Equipment
Step 2.33	Day 1 (15 minutes)	70% IPA	**Reagent spray** Spray accurately all reagents before placing them in the BSC.		Tissue Culture Lab (Grade B)	BSC
Step 2.34	Day 1 (10 minutes)	E8 basal media (1 x 500 mL)<br>E8 media supplement (10 mL)<br>10 mL stripette (x1)<br>50 mL stripette (x1)<br>50 mL conical tube (x1)	**Media preparation** Thaw E8 supplement overnight at 4°C. Add 10mL supplement to new bottle of E8 basal media. Using a 50 mL stripette, transfer 50 mL E8 Complete media to a 50 mL conical tube.	E8 Complete media (450 mL)<br>E8 Complete media aliquot (50 mL)<br>Waste	Tissue Culture Lab (Grade B)	4-8°C Fridge<br>BSC<br>Pipette boy
Step 2.35	Day 1 (5 minutes)	iPS cells in a 12-well plate (x 4 wells)<br>PBS (500 mL)<br>Aspirator straw (x1)<br>P1000 pipette tips (x 1 box)	**Cell wash** Using an aspirator, carefully remove the media by tilting the plate. Using a p1000 pipette and tilting the plate, add 1 mL PBS to each well.	iPS cells in a 12-well plate (x 4 wells)<br>PBS (496 mL)<br>Waste	Tissue Culture Lab (Grade B)	BSC<br>Aspirator<br>P1000 pipette
Step 2.36	Day 1 (5 minutes)	iPS cells in a 12-well plate (x 4 wells)<br>E8 Complete media aliquot (50 mL)<br>Aspirator straw (x1)<br>P1000 pipette (x 1 box)	**Media exchange** Using an aspirator, carefully remove PBS by tilting the plate. Using a p1000 pipette and tilting the plate, add 1 mL E8 complete media slowly to the edge of each well.<br><br>Place the flask in the incubator set at 37˚C, 5% CO_2.	iPS cells in a 12-well plate (x 4 wells)<br>Waste	Tissue Culture Lab (Grade B)	BSC<br>Incubator<br>Aspirator<br>P1000 pipette

Day 2-8: Resistant colonies selection

Step	Day and Duration	Materials in	Process Step	Materials Out	Location (Room and Grade)	Equipment
Step 2.37	Day -x		**Killing assay evaluation** A killing assay is initially performed to identify the concentrations of Puromycin and Geneticin to be used during the selection stage. A dilution curve is tested and the lowest concentration at which the cells die will be selected. For Puromycin, a range of concentrations between 0 µg/mL and 0.25 µg/mL will be tested; for Geneticin, a range of concentrations between 0 µg/mL and 500 µg/mL will be tested.	Puromycin concentration<br>Geneticin concentration	Tissue Culture Lab (Grade B)	
Step 2.38	Day 2-8 (15 minutes)	70% IPA	**Reagent spray** Spray accurately all reagents before placing them in the BSC.		Tissue Culture Lab (Grade B)	BSC
Step 2.39	Day 2-8 (5 minutes)	E8 basal media (7 x 500 mL)<br>E8 media supplement (7 x 10 mL)<br>10 mL stripette (x7)<br>50 mL stripette (x7)<br>50 mL conical tube (x7)	**Media preparation** Thaw E8 supplement overnight at 4°C. Add 10mL supplement to new bottle of E8 basal media. Using a 50 mL stripette, transfer 50 mL E8 Complete media to a 50 mL conical tube.	E8 Complete media (7 x 450 mL)<br>E8 Complete media aliquot (7 x 50 mL)<br>Waste	Tissue Culture Lab (Grade B)	4-8°C Fridge<br>BSC<br>Pipette boy

Day 2-8: Resistant colonies selection

Step	Day and Duration	Materials in	Process Step	Materials Out	Location (Room and Grade)	Equipment
Step 2.40	Day 2-8 (10 minutes)	E8 complete media aliquot (7 x 50 mL)<br>Rock inhibitor aliquot 10mM (7 x 591 µL)<br>Puromycin<br>Geneticin<br>25 mL Stripette (x7)<br>P200 pipette tips (7 x 1 box)<br>50 mL conical tube (x7)<br>Puromycin concentration<br>Geneticin concentration	**Selection media preparation**<br>With a 25 mL stripette, transfer 20 mL E8 media into a 50 mL conical tube. With a P200 pipette, add the required amount of puromycin and geneticin.	Rock inhibitor aliquot 10mM (7 x 571 µL)<br>Selection media (7 x 20 mL)<br>Waste	Tissue Culture Lab (Grade B)	BSC<br>P200 pipette<br>Pipette boy
Step 2.41	Day 2-8 (5 minutes)	iPS cells in a 12-well plate (x 4 wells)<br>PBS (7 x 500 mL)<br>Aspirator straw (x7)<br>P1000 pipette tips (7 x 1 box)	**Cell wash**<br>Using an aspirator, carefully remove the media by tilting the plate.<br>Using a p1000 pipette and tilting the plate, add 1 mL PBS.	PBS (7 x 496 mL)<br>Waste	Tissue Culture Lab (Grade B)	BSC<br>Aspirator<br>P1000 pipette
Step 2.42	Day 2-8 (5 minutes)	iPSC cells in a 12-well plate (x 4 wells)<br>Selection media (7 x 20 mL)<br>Aspirator straw (x7)<br>P1000 pipette (7 x 1box)	**Media exchange**<br>Using an aspirator, carefully remove PBS by tilting the plate. Using a p1000 pipette and tilting the plate, add 1 mL E8 media with Rock inhibitor slowly to the edge.<br><br>Place the plate in the incubator set at 37˚C, 5% CO_2.	iPSC cells in a 12-well plate (x 4 wells)<br>Waste	Tissue Culture Lab (Grade B)	BSC<br>Incubator<br>Aspirator<br>P1000 pipette

Day 9: Collection of individual resistant colonies

Step	Day and Duration	Materials in	Process Step	Materials Out	Location (Room and Grade)	Equipment
Step 2.43	Day 9 (15 minutes)	70% IPA	**Reagent spray** Spray accurately all reagents before placing them in the BSC.		Tissue Culture Lab (Grade B)	BSC
Step 2.44	Day 9 (5 minutes)	E8 basal media (1 x 500 mL); E8 media supplement (10 mL); Rock inhibitor 10 mM (1 x 591 µL vial); Vitronectin (1 x 1mL vial)	**Reagent preparation** Thaw E8 supplement overnight at 4°C. Take out media bottle from fridge and place at room temperature for 30 minutes. Add 10 mL supplement to new bottle of E8 basal media. Thaw one vial of Rock inhibitor at 4°C. Thaw one vial of vitronectin at room temperature.	E8 complete media (1 x 500 mL); Rock inhibitor 10 mM (1 x 591 µL vial); Vitronectin (1 x 1mL vial)	Tissue Culture Lab (Grade B)	4-8˚C Fridge
Step 2.45	Day 9 (5 minutes)	Thawed vitronectin vial (1 x 1 mL); PBS (1 x 500 mL); 25 mL stripette (x1); P200 pipette tips (x 1 box); 50 mL conical tube (x 1)	**Preparation of vitronectin-working solution** Using a 25 mL stripette, transfer 20 mL PBS into a 50 mL conical tube. Using a p200 pipette, add 200 µL vitronectin into the PBS solution.	Vitronectin working solution (1 x 20 mL); Waste	Tissue Culture Lab (Grade B)	BSC; Pipette boy; P200 pipette
Step 2.46	Day 9 (10 minutes)	Vitronectin working solution (1 x 20 mL); 12-well plate (x3); P1000 pipette tips (x 1 box)	**Preparation of vitronectin-coated plates** Using a p1000 pipette, transfer 500 µL of vitronectin working solution into each well of 3 x 12-well plate. Incubate at room temperature for 1 hour.	Vitronectin-coated 12-well plate (x3); Waste	Tissue Culture Lab (Grade B)	BSC; P1000 pipette

Day 9: Collection of individual resistant colonies

Step	Day and Duration	Materials in	Process Step	Materials Out	Location (Room and Grade)	Equipment
Step 2.47	Day 9 (10 minutes)	E8 complete media (1 x 500 mL)<br>Rock inhibitor 10 mM (1 x 591 µL)<br>50 mL Stripette (x1)<br>P200 pipette tips (1 box)<br>50 mL conical tube (x1)	**Media preparation**<br>With a 50 mL stripette, transfer 50 mL E8 complete media into a 50 mL conical tube. With a P200 pipette, add 50 µL Rock inhibitor.	E8 complete media (450 mL)<br>Rock inhibitor 10 mM (1 x 541 µL)<br>Supplemented E8 media (50 mL)<br>Waste	Tissue Culture Lab (Grade B)	BSC<br>P200 pipette<br>Pipette boy
Step 2.48	Day 9 (5 minutes)	iPS cells in a 12-well plate (x 4 wells)<br>PBS (500 mL)<br>Aspirator straw (x1)<br>P1000 pipette tips (1 box)	**Cell wash**<br>Using an aspirator, carefully remove the media by tilting the plate.<br>Using a p1000 pipette and tilting the plate, add 1 mL PBS. Using an aspirator, remove the PBS.	PBS (496 mL)<br>Waste	Tissue Culture Lab (Grade B)	BSC<br>Aspirator<br>P1000 pipette
Step 2.49	Day 9 (30 minutes)	iPS cells in a 12-well plate (x1 well)<br>Vitronectin-coated 12-well plates (x3)<br>Supplemented E8 media (50 mL)<br>P200 pipette tips (1 box)<br>10 mL stripette	**Seeding of individual colonies**<br>Using an aspirator, remove the vitronectin from the vitronectin-coated 12-well plates. Using a 10 mL stripette, transfer 1 mL E8 media into each well. Using a p200 pipette and 100 µL media, pick each individual colony and transfer it to a single well of the 12 well plates containing the media. Pipette to break down the colonies into single cells. Repeat this passage for 10 individual colonies for each edited well (total 30 colonies).<br><br>Place the plate in the incubator set at 37˚C, 5% CO_2.	iPS cells in 12-well plates (x3 plates)<br>Waste	Tissue Culture Lab (Grade B)	BSC<br>Aspirator<br>P200 pipette<br>Microscope<br>Pipette boy

Day 10-15: Media exchange

Step	Day and Duration	Materials in	Process Step	Materials Out	Location (Room and Grade)	Equipment
Step 2.50	Day 10-15<br>(15 minutes)	70% IPA	**Reagent spray**<br>Spray accurately all reagents before placing them in the BSC.		Tissue Culture Lab<br>(Grade B)	BSC
Step 2.51	Day 10-15<br>(10 minutes)	E8 basal media (6 x 500 mL)<br>E8 media supplement (6 x 10 mL)<br>10 mL stripette (x6)<br>50 mL stripette (x6)<br>50 mL conical tube (x6)	**Media preparation**<br>Thaw E8 supplement overnight at 4°C. Add 10mL supplement to new bottle of E8 basal media.<br>Using a 50 mL stripette, transfer 50 mL E8 Complete media to a 50 mL conical tube.	E8 Complete media (6 x 450 mL)<br>E8 Complete media aliquot (6 x 50 mL)<br>Waste	Tissue Culture Lab<br>(Grade B)	4-8°C Fridge<br>BSC<br>Pipette boy
Step 2.52	Day 10-15<br>(10 minutes)	iPS cells in a 12-well plate (x 3 plates)<br>PBS (6 x 500 mL)<br>Aspirator straw (x6)<br>10 mL stripette (x18)	**Cell wash**<br>Using an aspirator, carefully remove the media by tilting the plate.<br>Using a 10 mL stripette and tilting the plate, add 1 mL PBS to each well.	PBS (6 x 470 mL)<br>iPS cells in a 12-well plate (x 3 plates)<br>Waste	Tissue Culture Lab<br>(Grade B)	BSC<br>Aspirator<br>Pipette boy
Step 2.53	Day 10-15<br>(5 minutes)	iPS cells in a 12-well plate (x 3 plates)<br>E8 Complete media aliquot (6 x 50 mL)<br>Aspirator straw (x6)<br>10 mL stripette (x18)	**Media exchange**<br>Using an aspirator, carefully remove PBS by tilting the plate. Using a 10 mL stripette and tilting the plate, add 1 mL E8 complete media slowly to the edge of each well.<br><br>Place the flask in the incubator set at 37˚C, 5% CO_2.	iPS cells in a 12-well plate (x 3 plates)<br>Waste	Tissue Culture Lab<br>(Grade B)	BSC<br>Incubator<br>Aspirator<br>P1000 pipette

Day 16: Homozygous clone selection

	Day and Duration	Materials in	Process Step	Materials Out	Location (Room and Grade)	Equipment
Step 2.54	Day 16 (15 minutes)	70% IPA	**Reagent spray** Spray accurately all reagents before placing them in the BSC.		Tissue Culture Lab (Grade B)	BSC
Step 2.55	Day 16 (5 minutes)	E8 basal media (1 x 500 mL)<br>E8 media supplement (10 mL)<br>10 mL stripette (x1)<br>50 mL stripette (x1)<br>50 mL conical tube (x1)	**Media preparation** Thaw E8 supplement overnight at 4°C. Add 10mL supplement to new bottle of E8 basal media. Using a 50 mL stripette, transfer 50 mL E8 Complete media to a 50 mL conical tube.	E8 Complete media (450 mL)<br>E8 Complete media aliquot (50 mL)<br>Waste	Tissue Culture Lab (Grade B)	4-8°C Fridge<br>BSC<br>Pipette boy
Step 2.56	Day 16 (5 minutes)	KOSR (1 x 100 mL)<br>DMSO (1 x 5 mL)<br>25 mL stripettes (x1)<br>5 mL stripettes (x1)<br>50 mL conical tube (x1)	**Freezing media B preparation** Using a 25 mL stripette, transfer 16 mL KOSR into a 50 mL conical tube. Using a 5 mL stripette, add 4 mL DMSO.	KOSR (1 x 84 mL)<br>Freezing media B (1 x 20 mL)<br>Waste	Tissue Culture Lab (Grade B)	BSC<br>Pipette boy
Step 2.57	Day 16 (10 minutes)	Plated iPS cells into 12-well plates (x 3 plates)<br>PBS (1 x 500 mL)<br>Aspirator straw (x1)<br>10 mL stripette (x3)	**Cell wash** Using an aspirator, carefully remove the media by tilting the flask. Using a 10 mL stripette and tilting the flask, add 1 mL PBS to each well.	PBS (1 x 470 mL)<br>Waste	Tissue Culture Lab (Grade B)	BSC<br>Aspirator<br>Pipette boy
Step 2.58	Day 16 (10 minutes)	TrypLE (100 mL)<br>Aspirator straw (x1)<br>10 mL stripette (x3)	**Cell harvest** Using an aspirator, carefully remove the PBS by tilting the flask. Using a 10 mL stripette, add 1 mL of TrypLE to each well. Incubate for 3 minutes in the incubator.	TrypLE (1 x 70 mL)<br>Waste	Tissue Culture Lab (Grade B)	BSC<br>Aspirator<br>Pipette boy<br>Incubator

Day 16: Homozygous clone selection

	Day and Duration	Materials in	Process Step	Materials Out	Location (Room and Grade)	Equipment
Step 2.59	Day 16<br>(5 minutes)		Visual inspection at the microscope<br>Are cells rounded and loosely attached to the plate?<br>NO: Incubate for 2 minutes in the incubator<br>YES		Tissue Culture Lab<br>(Grade B)	Microscope<br>Incubator
Step 2.60	Day 16<br>(15 minutes)	E8 complete media aliquot (1 x 50 mL)<br>Aspirator straw (x1)<br>P1000 pipette tips (x 1 box)<br>1.5 mL Eppendorf tube (x33)<br>P10 pipette tips (x 1 box)	**Cell harvest**<br>Carefully tilt the plate and remove TrypLE from the well by aspiration. Using a p1000 pipette, add 1 mL E8 media on each well and pipette on the surface until cells dissociate from the plate. Using a p1000 pipette transfer the cell solution from each well to a 1.5 mL Eppendorf tube. Using a p10 pipette, transfer 10 µL cell solution from 3 different wells to 1.5 mL Eppendorf tubes.	iPS cells in suspension (30 x 1 mL)<br>iPS cells aliquots for counting (3 x 10 µL)<br>E8 complete media (20 mL)<br>Waste	Tissue Culture Lab<br>(Grade B)	BSC<br>P1000 pipette<br>P10 pipette<br>Aspirator
Step 2.61	Day 16<br>(15 minutes)	iPS cells aliquots for counting (3 x 10 µL)<br>Trypan Blue (1 mL)<br>P10 pipette tips (x1 box)	**Cell counting**<br>Using a p10 pipette, combine each aliquot of 10 µL cells with 10 µL Trypan Blue. Count cells using an haemocytometer	Total cell number N_{cell}<br>Waste	Tissue Culture Lab<br>(Grade B)	Haemacytometer<br>P10 pipette<br>Microscope

Day 16: Homozygous clone selection

	Day and Duration	Materials in	Process Step	Materials Out	Location (Room and Grade)	Equipment
Step 2.62	Day 16 (5 minutes)	Total cell number N_{cell}	**Calculation of cell volume for banking** Based on the average cell number, measured with the haemocytometer, calculate the volume of cell solution containing 1E06 cells (V_{cell}).	Volume containing 1E06 cells V_{cell}	Tissue Culture Lab (Grade B)	Calculator
Step 2.63	Day 16 (15 minutes)	iPS cells in suspension (30 x 1 mL)<br>Volume containing 1E06 cells (V_{cell})<br>P1000 pipette tips (x 1 box)<br>1.5 mL Eppendorf tube (x30)	**Cell aliquoting** Using a p1000 pipette, transfer V_{cell} for each vial to new 1.5 mL Eppendorf tubes. Centrifuge all the samples at 300g for 5 minutes.	iPS cells pellet for banking (x30)<br>iPS cells pellet for genotyping (x30)<br>Waste	Tissue Culture Lab (Grade B)	BSC<br>P1000 pipette<br>Centrifuge
Step 2.64	Day 16 (15 minutes)	iPS cells pellet for banking (x30)<br>KOSR (1 x 84 mL)<br>Freezing media B (20 mL)<br>P1000 pipette tips (x1 box)<br>Cryovial (x30)<br>Coolcell	**Cell resuspension into freezing media** Gently flick each tube to fully dislodge the pellet from the tube bottom. Using a p1000 pipette resuspend the cells into 500 µL of KOSR (Freezing media A). Following uniform suspension, using a p1000 pipette, add 500 µL of Freezing media B in a drop-wise manner. Using a p1000 pipette, transfer 1 mL cell solution to each cryovial.<br><br>Quickly place the vial into a Coolcell and transfer to -80°C overnight. Transfer the cells to a liquid nitrogen tank for long-term storage.	Frozen iPS cells (x30 cryovials)<br>Waste<br>Coolcell	Tissue Culture Lab (Grade B)<br>Liquid nitrogen storage	BSC<br>P1000 pipette<br>Coolcell<br>Liquid nitrogen tank

Day 16: Homozygous clone selection

	Day and Duration	Materials in	Process Step	Materials Out	Location (Room and Grade)	Equipment
Step 2.65		iPS cells pellet for genotyping (x30)	**Genotyping** Perform DNA extraction and 7-step PCR to genotype each individual clone.	Genotyping data	Analytical Lab	PCR machine
Step 2.66		Genotyping data	Is clone homozygous for the editing? NO → Rejected Product; YES → Go to Step 2.67.	Rejected Product		

iPS cells thawing

Step	Day and Duration	Materials in	Process Step	Materials Out	Location (Room and Grade)	Equipment
Step 2.67	Day x (15 minutes)	70% IPA	**Reagent spray** Spray accurately all reagents before placing them in the BSC.		Tissue Culture Lab (Grade B)	BSC
Step 2.68	Day x (5 minutes)	E8 basal media (1 x 500 mL); E8 media supplement (10 mL); Rock inhibitor 10 mM (1 x 591 µL vial); Vitronectin (1 x 1mL vial)	**Reagent preparation** Thaw E8 supplement overnight at 4°C. Take out media bottle from fridge and place at room temperature for 30 minutes. Add 10 mL supplement to new bottle of E8 basal media. Thaw one vial of Rock inhibitor at 4°C. Thaw one vial of vitronectin at room temperature.	E8 complete media (1 x 500 mL); Rock inhibitor 10 mM (1 x 591 µL vial); Vitronectin (1 x 1mL vial)	Tissue Culture Lab (Grade B)	4-8˚C Fridge
Step 2.69	Day x (5 minutes)	Thawed vitronectin vial (1 x 1 mL); PBS (1 x 500 mL); 10 mL stripette (x1); P200 pipette tips (x1 box); 15 mL conical tube (x 1)	**Preparation of vitronectin-working solution** Using a 10 mL stripette, transfer 6 mL PBS into a 15 mL conical tube. Using a p200 pipette, add 60 µL vitronectin into the PBS solution.	Vitronectin working solution (1 x 6 mL); Waste	Tissue Culture Lab (Grade B)	BSC; Pipette boy; P200 pipette
Step 2.70	Day x (60 min)	Vitronectin working solution (1 x 6 mL); 6-well plate (x1); 10 mL stripette (x1)	**Preparation of vitronectin-coated plates** Using a 10 mL stripette, transfer 1 mL of vitronectin working solution into each well of a 6-well plate. Incubate at room temperature for 1 hour.	Vitronectin-coated 6-well plate (x1); Waste	Tissue Culture Lab (Grade B)	BSC; Pipette boy

iPS cells thawing

Step	Day and Duration	Materials in	Process Step	Materials Out	Location (Room and Grade)	Equipment
Step 2.71	Day x (10 minutes)	E8 complete media (1 x 500 mL)<br>Rock inhibitor (1 x 591 µL)<br>50 mL stripette (x1)<br>P200 pipette tips (x 1 box)<br>50 mL conical tube	**Media preparation** Using a 50 mL stripette, transfer 50 mL E8 complete media to a 50 mL conical tube. Using a p200 pipette, add 50 µL Rock inhibitor.	E8 complete media (450 mL)<br>Rock inhibitor (1 x 541 µL)<br>Supplemented E8 media (50 mL)<br>Waste	Tissue Culture Lab (Grade B)	BSC<br>Pipette boy<br>P200 pipette
Step 2.72	Day x (5 minutes)	iPS cells vial (x1)	**Cell retrieval** Remove the vial from the liquid nitrogen and immediately place on dry ice.		Liquid Nitrogen storage room	Dry ice<br>Liquid Nitrogen vessel
Step 2.73	Day x (5 minutes)	iPS cells vial (x1)	**Cell thaw** Partially submerge vial into a water bath at 37˚C. Inspect the vial till it is half or 2/3 thawed.	Thawed iPS cells vial (x1)	Tissue Culture Lab (Grade B)	Water bath
Step 2.74	Day x (5 minutes)	E8 complete media (450 mL)<br>Thawed iPS cells vial (x1)<br>15 mL conical tube (x1)<br>P1000 pipette tops (1 box)<br>10 mL stripette (x1)	**Cell harvest** Using a 10mL stripette, transfer 10mL E8 complete media into a 15 mL conical tube. Using a p1000 pipette, transfer the thawed iPS cells (1 mL) to the 15 mL tube containing the media.	E8 complete media (440 mL)<br>iPS cells in suspension (1 x 15 mL conical tube)<br>Waste	Tissue Culture Lab (Grade B)	BSC<br>Pipette boy<br>P1000 pipette

iPS cells thawing

	Day and Duration	Materials in	Process Step	Materials Out	Location (Room and Grade)	Equipment
Step 2.75	Day x (10 minutes)	iPS cells in suspension (1 x 15 mL conical tube)<br>Supplemented E8 media (50 mL)<br>Vitronectin-coated 6-well plate (x1)<br>P1000 pipette tips (x1 box)<br>Aspirator straw (x2)<br>5 mL stripette (x1)	**Cell seeding**<br>Centrifuge cells at 300g for 5 minutes. Using an aspirator, remove the media without touching the pellet. Using a p1000 pipette, resuspend cells into 1 mL of Supplemented E8 media with Rock inhibitor. Using an aspirator, remove the vitronectin from a vitronectin-coated 6-well plate. Using a 5 mL stripette, transfer 1 mL of supplemented E8 media to 2 wells of a vitronectin-coated 6-well plate. Using a p1000 pipette, transfer 500 µL of cell solution into each well.<br><br>Place the plate in the incubator set at 37°C, 5% CO_2. Mix the culture by cross-shaped movements to distribute cells evenly.	iPS cells into plates (1 x 6-well plate)<br>Supplemented E8 media (45 mL)<br>Waste	Tissue Culture Lab (Grade B)	BSC<br>Aspirator<br>P1000 pipette<br>Incubator<br>Centrifuge<br>Pipet boy

Day x: Expansion

Step	Day and Duration	Materials in	Process Step	Materials Out	Location (Room and Grade)	Equipment
Step 2.76	Day x (15 minutes)	70% IPA	**Reagent spray** Spray accurately all reagents before placing them in the BSC.		Tissue Culture Lab (Grade B)	BSC
Step 2.77	Day x (10 minutes)	iPS cells in 6-well plate (x N wells)	**Microscope observation** Take the cells from the incubator and observe under the microscope<br>Is cell confluency >85%? YES → Go to Step 2.80 (Cell splitting); NO → next step	iPS cells in 6-well plate (x N wells)<br>Go to Step 2.80 (Cell splitting)		Incubator<br>Microscope
Step 2.78	Day x (10 minutes)	E8 basal media (1 x 500 mL)<br>E8 media supplement (10 mL)<br>10 mL stripette (x1)<br>50 mL stripette (x1)<br>50 mL conical tube (x1)	**Media preparation** Thaw E8 supplement overnight at 4°C. Add 10mL supplement to new bottle of E8 basal media. Using a 50 mL stripette, transfer 50 mL E8 Complete media to a 50 mL conical tube.	E8 Complete media (450 mL)<br>E8 Complete media aliquot (50 mL)<br>Waste	Tissue Culture Lab (Grade B)	4-8°C Fridge<br>BSC<br>Pipette boy
Step 2.79		iPS cells in a 6-well plate (x n well)<br>E8 Complete media aliquot (50 mL)<br>Aspirator straw (x1)<br>10 mL stripette (x1)	**Media exchange** Using an aspirator, carefully remove the old media by tilting the plate. Using a 10 mL stripette and tilting the plate, add 1 mL E8 complete media slowly to the edge of each well.<br><br>Place the flask in the incubator set at 37˚C, 5% CO_2.	iPS cells in a 6-well plate (x n well)<br>Waste	Tissue Culture Lab (Grade B)	BSC<br>Incubator<br>Aspirator<br>Pipette boy

Day x: Cell splitting

	Day and Duration	Materials in	Process Step	Materials Out	Location (Room and Grade)	Equipment
Step 2.80	Day x<br>(5 minutes)	E8 basal media (1 x 500 mL)<br>E8 media supplement (10 mL)<br>Rock inhibitor 10 mM (1 x 591 µL vial)<br>Vitronectin (1 x 1mL vial)	**Reagent preparation**<br>Thaw E8 supplement overnight at 4°C. Take out media bottle from fridge and place at room temperature for 30 minutes. Add 10 mL supplement to new bottle of E8 basal media. Thaw one vial of Rock inhibitor at 4°C. Thaw one vial of vitronectin at room temperature.	E8 complete media (1 x 500 mL)<br>Rock inhibitor 10 mM (1 x 591 µL vial)<br>Vitronectin (1 x 1mL vial)	Tissue Culture Lab (Grade B)	4-8˚C Fridge
Step 2.81	Day x<br>(10 minutes)	E8 complete media (1 x 500 mL)<br>Rock inhibitor (1 x 591 µL)<br>50 mL stripette (x1)<br>P200 pipette tips (x 1 box)<br>50 mL conical tube (x1)	**Media preparation**<br>Using a 50 mL stripette, transfer 50 mL E8 media to a 50 mL conical tube. Using a p200 pipette, add 50 µL Rock inhibitor.	E8 complete media (450 mL)<br>Rock inhibitor (1 x 541 µL)<br>Supplemented E8 media (50 mL)<br>Waste	Tissue Culture Lab (Grade B)	BSC<br>Pipette boy<br>P200 pipette
Step 2.82	Day x<br>(10 minutes)	Thawed vitronectin vial (1 x 1 mL)<br>PBS (1 x 500 mL)<br>50 mL stripette (x1)<br>P1000 pipette tips (x1 box)<br>50 mL conical tube (x 1)	**Preparation of vitronectin-working solution**<br>Using a 50 mL stripette, transfer 50 mL PBS into a 50 mL conical tube. Using a p1000 pipette, add 500 µL vitronectin into the PBS solution.	PBS (1 x 450 mL)<br>Vitronectin working solution (1 x 50 mL)<br>Waste	Tissue Culture Lab (Grade B)	BSC<br>Pipette boy<br>P1000 pipette
Step 2.83	Day x<br>(60 min)	Vitronectin working solution (1 x 50 mL)<br>6-well plate (10 x N wells)<br>10 mL stripette (x1)	**Preparation of vitronectin-coated plates**<br>Using a 10 mL stripette, transfer 1 mL of vitronectin working solution into each well of a 6-well plate. Incubate at room temperature for 1 hour.	Vitronectin-coated 6-well plate (10 x N wells)<br>Waste	Tissue Culture Lab (Grade B)	BSC<br>Pipette boy

Day x: Cell splitting

	Day and Duration	Materials in	Process Step	Materials Out	Location (Room and Grade)	Equipment
Step 2.84	Day x<br>(5 minutes)	iPS cells in 6-well plate (x N wells)<br>PBS (1 x 500 mL)<br>Aspirator straw (x1)<br>P1000 pipette tips (x 1box)	**Cell wash**<br>Using an aspirator, carefully remove the media by tilting the flask.<br>Using a 10 mL stripette and tilting the flask, add 1 mL PBS to each well.	PBS (500 – N_{wells} mL)<br>Waste	Tissue Culture Lab (Grade B)	BSC<br>Aspirator<br>P1000 pipette
Step 2.85	Day x<br>(10 minutes)	TrypLE (1 x 100 mL)<br>Aspirator straw (x1)<br>P1000 pipette (x 1box)	**Cell harvest**<br>Using an aspirator, carefully remove the PBS by tilting the flask.<br>Using a 10 mL stripette, add 1 mL of TrypLE to each well.<br>Incubate for 3 minutes in the incubator.	TrypLE (100 – N_{wells} mL)<br>Waste	Tissue Culture Lab (Grade B)	BSC<br>Aspirator<br>Pipette boy<br>Incubator
Step 2.86	Day x<br>(5 minutes)		Visual inspection at the microscope<br>Are cells rounded and loosely attached to the plate?<br>NO → Incubate for 2 minutes in the incubator. (return to visual inspection)<br>YES → next step		Tissue Culture Lab (Grade B)	Microscope<br>Incubator
Step 2.87	Day x<br>(10 minutes)	Supplemented E8 media (50 mL)<br>Aspirator straw (x1)<br>P1000 pipette tips (x1 box)<br>15 mL conical tube (x1)<br>P10 pipette tips (x 1 box)<br>1.5 mL Eppendorf tube (x1)	**Cell harvest**<br>Carefully tilt the plate and remove the TrypLE from the well by aspiration.<br>Using a p1000 pipette, add 1 mL supplemented E8 media and pipette on the surface until cells dissociate from the plate.<br>Using a p1000 pipette transfer the cell solution to a 15 mL conical tube.<br>Using a P10 pipette, transfer 10µL cells solution to a new 1.5 mL Eppendorf tube.	iPS cells in suspension (N_{wells} mL)<br>iPS cells aliquot for counting (10 µL)<br>Supplemented E8 media (50- N_{wells} mL)<br>Waste	Tissue Culture Lab (Grade B)	BSC<br>P10 pipette<br>P1000 pipette<br>Aspirator

Day x: Cell splitting

Step	Day and Duration	Materials in	Process Step	Materials Out	Location (Room and Grade)	Equipment
Step 2.88	Day x (10 minutes)	iPS cells aliquot for counting (10 µL)<br>Trypan Blue (1 mL)<br>P10 pipette tips (x1 box)	**Cell counting**<br>Using a p10 pipette, combine 10 µL cells with 10 µL Trypan Blue. Count cells using an haemocytometer<br><br>Is total cell number >100E06? YES → Go to step 2.90 (Cell banking); NO → Cell seeding	Total cell number N_{cell}<br>Waste	Tissue Culture Lab (Grade B)	Haemacytometer<br>P10 pipette<br>Microscope
Step 2.89	Day x (20 minutes)	iPS cells in suspension (N_{wells} mL)<br>Vitronectin-coated 6-well plate (10 x N wells)<br>Supplemented E6 media (50- N_{wells} mL)<br>10 mL stripette (x1)<br>P1000 pipette tips (x1 box)<br>Aspirator straw (x1)	**Cell seeding**<br>For each passage, calculate the number of wells to be seeded as:<br><br>$N_{seed} = N_{harvest}$ x 10<br><br>Where $N_{harvest}$ is the number of wells from the previous passage.<br><br>Using an aspirator, remove the vitronectin from the vitronectin-coated 6-well plates. Using a 10 mL stripette, transfer 1.5 mL supplemented E8 media to each well. Using a P200 pipette, transfer 100µL cells solution to each well.<br><br>Place the plate in the incubator set at 37˚C, 5% CO2. Mix the culture by cross-shaped movements to distribute cells evenly.<br><br>Go to Step 2.76 (repeat till required cell number is met)	iPS cells in 6-well plates (10 x N_{wells})<br>Waste	Tissue Culture Lab (Grade B)	Calculator<br>BSC<br>P200 pipette<br>Pipette boy<br>Incubator<br>Aspirator

Cryopreservation

Step	Day and Duration	Materials in	Process Step	Materials Out	Location (Room and Grade)	Equipment
Step 2.90	Day x (5 minutes)	Total cell number N_{cell}	**Calculation of the volume of freezing media required** Based on the cell number, measured with the haemocytometer, calculate the volume of freezing solution, in order to resuspend the cells at a concentration of 1E06 cells/mL	$V_{freezing\ solution}$		Calculator
Step 2.91	Day x (5 minutes)	KOSR (1 x 100 mL); DMSO (1 x 5 mL); 25 mL stripette (x1); 10 mL stripette (x1); 50 mL conical tube (x1)	**Freezing media B preparation** Using a 25 mL stripette, transfer 20 mL KOSR into a 50 mL conical tube. Using a 10 mL stripette, add 5 mL DMSO.	KOSR (1 x 80 mL); Freezing media B (25 mL); Waste	Tissue Culture Lab (Grade B)	BSC; Pipette boy
Step 2.92	Day x (20 minutes)	iPS cells in suspension (n mL); KOSR (1 x 80 mL); Freezing media B (25 mL); Aspirator straw (x1); 10 mL stripette (x2); Cryovial (x 100); Coolcell; P1000 pipette tips (2 x 1box)	**Cell resuspension into freezing media** Centrifuge cells at 300g for 5 minutes. Using an aspirator, remove the supernatant. Gently flick the tube to fully dislodge the pellet from the tube bottom. Using a 10 mL stripette, resuspend the cells into ½ $V_{freezing\ solution}$ of KOSR. Following uniform suspension, using a 10 mL stripette, add ½ $V_{freezing\ solution}$ of Freezing media B in a drop-wise manner. Using a p1000 pipette, transfer 1 mL cell solution to each cryovial.<br><br>Quickly place the vial into a Coolcell and transfer to -80˚C overnight. Transfer the cells to a liquid nitrogen tank for long-term storage.	Frozen iPS cells (x 100 cryovials); Waste; Coolcell	Tissue Culture Lab (Grade B); Liquid nitrogen storage	BSC; Aspirator; P1000 pipette; Coolcell; Liquid nitrogen tank

iPS cells thawing

	Day and Duration	Materials in	Process Step	Materials Out	Location (Room and Grade)	Equipment
Step 3.1	Day -x<br>(15 minutes)	70% IPA	**Reagent spray**<br>Spray accurately all reagents before placing them in the BSC.		Tissue Culture Lab<br>(Grade B)	BSC
Step 3.2	Day -x<br>(5 minutes)	E8 basal media (1 x 500 mL)<br>E8 media supplement (10 mL)<br>Rock inhibitor 10 mM (1 x 591 µL vial)<br>Vitronectin (1 x 1mL vial)	**Reagent preparation**<br>Thaw E8 supplement overnight at 4°C. Take out media bottle from fridge and place at room temperature for 30 minutes. Add 10 mL supplement to new bottle of E8 basal media. Thaw one vial of Rock inhibitor at 4°C. Thaw one vial of vitronectin at room temperature.	E8 complete media (1 x 500 mL)<br>Rock inhibitor 10 mM (1 x 591 µL vial)<br>Vitronectin (1 x 1mL vial)	Tissue Culture Lab<br>(Grade B)	4-8˚C Fridge
Step 3.3	Day -x<br>(5 minutes)	Thawed vitronectin vial (1 x 1 mL)<br>PBS (1 x 500 mL)<br>10 mL stripette (x1)<br>P200 pipette tips (x1 box)<br>15 mL conical tube (x 1)	**Preparation of vitronectin-working solution**<br>Using a 10 mL stripette, transfer 6 mL PBS into a 15 mL conical tube. Using a p200 pipette, add 60 µL vitronectin into the PBS solution.	Vitronectin working solution (1 x 6 mL)<br>Waste	Tissue Culture Lab<br>(Grade B)	BSC<br>Pipette boy<br>P200 pipette
Step 3.4	Day -x<br>(60 min)	Vitronectin working solution (1 x 6 mL)<br>6-well plate (x1)<br>10 mL stripette (x1)	**Preparation of vitronectin-coated plates**<br>Using a 10 mL stripette, transfer 1 mL of vitronectin working solution into each well of a 6-well plate. Incubate at room temperature for 1 hour.	Vitronectin-coated 6-well plate (x1)<br>Waste	Tissue Culture Lab<br>(Grade B)	BSC<br>Pipette boy

iPS cells thawing

	Day and Duration	Materials in	Process Step	Materials Out	Location (Room and Grade)	Equipment
Step 3.5	Day -x (10 minutes)	E8 complete media (1 x 500 mL)<br>Rock inhibitor (1 x 591 µL)<br>50 mL stripette (x1)<br>P200 pipette tips (x 1 box)<br>50 mL conical tube	**Media preparation**<br>Using a 50 mL stripette, transfer 50 mL E8 media to a 50 mL conical tube. Using a p200 pipette, add 50 µL Rock inhibitor.	E8 complete media (450 mL)<br>Rock inhibitor (1 x 541 µL)<br>Supplemented E8 media (50 mL)<br>Waste	Tissue Culture Lab (Grade B)	BSC<br>Pipette boy<br>P200 pipette
Step 3.6	Day -x (5 minutes)	iPS cells vial (x1)	**Cell retrieval**<br>Remove the vial from the liquid nitrogen and immediately place on dry ice.		Liquid Nitrogen storage room	Dry ice<br>Liquid Nitrogen vessel
Step 3.7	Day -x (5 minutes)	iPS cells vial (x1)	**Cell thaw**<br>Partially submerge vial into a water bath at 37˚C. Inspect the vial till it is half or 2/3 thawed.	Thawed iPS cells vial (x1)	Tissue Culture Lab (Grade B)	Water bath
Step 3.8	Day -x (5 minutes)	E8 complete media (450 mL)<br>Thawed iPS cells vial (x1)<br>15 mL conical tube (x1)<br>P1000 pipette tops (1 box)<br>10 mL stripette (x1)	**Cell harvest**<br>Using a 10mL stripette, transfer 10mL E8 basal media into a 15 mL conical tube. Using a p1000 pipette, transfer the thawed iPS cells (1 mL) to the 15 mL tube containing the media.	E8 complete media (440 mL)<br>iPS cells in suspension (1 x 15 mL conical tube)<br>Waste	Tissue Culture Lab (Grade B)	BSC<br>Pipette boy<br>P1000 pipette

iPS cells thawing

	Day and Duration	Materials in	Process Step	Materials Out	Location (Room and Grade)	Equipment
Step 3.9	Day -x (10 minutes)	iPS cells in suspension (1 x 15 mL conical tube)<br>Supplemented E8 media (50 mL)<br>Vitronectin-coated 6-well plate (x1)<br>P1000 pipette tips (x1 box)<br>Aspirator straw (x2)<br>5 mL stripette (x1)	**Cell seeding**<br>Centrifuge cells at 300g for 5 minutes. Using an aspirator, remove the media without touching the pellet. Using a p1000 pipette, resuspend cells into 1 mL of Supplemented E8 media with Rock inhibitor. Using an aspirator, remove the vitronectin from a vitronectin-coated 6-well plate. Using a 5 mL stripette, transfer 1 mL of supplemented E8 media to 2 wells of a vitronectin-coated 6-well plate. Using a p1000 pipette, transfer 500 µL of cell solution into each well.<br><br>Place the plate in the incubator set at 37˚C, 5% CO_2. Mix the culture by cross-shaped movements to distribute cells evenly.	iPS cells into plates (1 x 6-well plate)<br>Supplemented E8 media (45 mL)<br>Waste	Tissue Culture Lab (Grade B)	BSC<br>Aspirator<br>P1000 pipette<br>Incubator<br>Centrifuge<br>Pipet boy

Day -x: iPS cells expansion

Step	Day and Duration	Materials in	Process Step	Materials Out	Location (Room and Grade)	Equipment
Step 3.10	Day -x (15 minutes)	70% IPA	**Reagent spray** Spray accurately all reagents before placing them in the BSC.		Tissue Culture Lab (Grade B)	BSC
Step 3.11	Day -x (10 minutes)	iPS cells in 6-well plate (x N_{wells})	**Microscope observation** Take the cells from the incubator and observe under the microscope<br>Is cell confluency >85%? YES → Go to Step 3.15 (Cell splitting); NO → next step	iPS cells in 6-well plate (x N_{wells})<br>Go to Step 3.15 (Cell splitting)		Incubator<br>Microscope
Step 3.12	Day -x (5 minutes)		**Calculation of total E8 media volume** The volume of E8 media required is the same as the number of wells that are in culture:<br>$Vol_{E8media}$ (mL) = N_{wells}	E8 media Volume $Vol_{E8media}$		Calculator
Step 3.13		E8 basal media (1 x 500 mL)<br>E8 media supplement (10 mL)<br>10 mL stripette (x1)<br>50 mL stripette (x1)<br>50 mL conical tube (x1)<br>E8 media Volume $Vol_{E8media}$	**Media preparation** Thaw E8 supplement overnight at 4°C. Add 10mL supplement to a new bottle of E8 basal media. Using a 50 mL stripette, transfer $Vol_{E8media}$ (mL) E8 Complete media to a 50 mL conical tube.	E8 Complete media (450 mL)<br>E8 Complete media aliquot ($Vol_{E8media}$)<br>Waste	Tissue Culture Lab (Grade B)	4-8°C Fridge<br>BSC<br>Pipette boy

Day -x: iPS cells expansion

	Day and Duration	Materials in	Process Step	Materials Out	Location (Room and Grade)	Equipment
Step 3.14	Day -x (10 minutes)	iPS cells in a 6-well plate (x N_{well})<br>E8 Complete media aliquot ($Vol_{E8media}$)<br>Aspirator straw (x1)<br>10 mL stripette (x1)	**Media exchange** Using an aspirator, carefully remove PBS by tilting the plate. Using a 10 mL stripette and tilting the plate, add 1 mL E8 complete media slowly to the edge of each well.<br><br>Place the flask in the incubator set at 37˚C, 5% CO_2.<br><br>Return to Step 3.10	iPS cells in a 6-well plate (x N_{well})<br>Waste	Tissue Culture Lab (Grade B)	BSC<br>Incubator<br>Aspirator<br>Pipette boy

Day -x: Cell splitting

Step	Day and Duration	Materials in	Process Step	Materials Out	Location (Room and Grade)	Equipment
Step 3.15	Day -x (5 minutes)		**Calculation of total E8 media and vitronectin volume** The volume of E8 media and the volume of vitronectin required are calculated from the number of wells that are in culture as: $Vol_{E8media}$ (mL) = $Vol_{vitronectin}$ (mL) = 10 x N_{wells}	E8 media Volume $Vol_{E8media}$<br>Vitronectin Volume $Vol_{Vitronectin}$		Calculator
Step 3.16	Day -x (5 minutes)	E8 basal media (1 x 500 mL)<br>E8 media supplement (10 mL)<br>Rock inhibitor 10 mM (1 x 591 µL vial)<br>Vitronectin (1 x 1mL vial)	**Reagent preparation** Thaw E8 supplement overnight at 4°C. Take out media bottle from fridge and place at room temperature for 30 minutes. Add 10 mL supplement to new bottle of E8 basal media. Thaw one vial of Rock inhibitor at 4°C. Thaw one vial of vitronectin at room temperature.	E8 complete media (1 x 500 mL)<br>Rock inhibitor 10 mM (1 x 591 µL vial)<br>Vitronectin (1 x 1 mL vial)	Tissue Culture Lab (Grade B)	4-8 °C Fridge
Step 3.17	Day -x (10 minutes)	E8 complete media<br>Rock inhibitor<br>50 mL stripette (x1)<br>P200 pipette tips (x 1 box)<br>50 mL conical tube (x1)<br>E8 media Volume $Vol_{E8media}$	**Media preparation** Transfer $Vol_{E8media}$ to a conical tube. Add 1 µL/mL of Rock inhibitor.	E8 complete media<br>Rock inhibitor<br>Supplemented E8 media ($Vol_{E8media}$)<br>Waste	Tissue Culture Lab (Grade B)	BSC<br>Pipette boy<br>P200 pipette
Step 3.18	Day -x (10 minutes)	Vitronectin vial<br>PBS<br>50 mL stripette (x1)<br>P1000 pipette tips (x1 box)<br>50 mL conical tube (x 1)<br>Vitronectin Volume $Vol_{Vitronectin}$	**Preparation of vitronectin-working solution** Transfer $Vol_{Vitronectin}$ of PBS to a conical tube. Add 10 µL/mL of vitronectin and mix well.	PBS<br>Vitronectin working solution ($Vol_{Vitronectin}$)<br>Waste	Tissue Culture Lab (Grade B)	BSC<br>Pipette boy<br>P1000 pipette

Day -x: Cell splitting

Step	Day and Duration	Materials in	Process Step	Materials Out	Location (Room and Grade)	Equipment
Step 3.19	Day -x (60 min)	Vitronectin working solution ($Vol_{Vitronectin}$)<br>6-well plate (10 x N_{wells})<br>10 mL stripette	**Preparation of vitronectin-coated plates**<br>Using a 10 mL stripette, transfer 1 mL of vitronectin working solution into each well of a 6-well plate. Incubate at room temperature for 1 hour.	Vitronectin-coated 6-well plate (10 x N_{wells})<br>Waste	Tissue Culture Lab (Grade B)	BSC<br>Pipette boy
Step 3.20	Day -x (5 minutes)	iPS cells in 6-well plate (x N_{wells})<br>PBS (1 x 500 mL)<br>Aspirator straw (x1)<br>P1000 pipette tips (x 1box)	**Cell wash**<br>Using an aspirator, carefully remove the media by tilting the flask.<br>Using a 10 mL stripette and tilting the flask, add 1 mL PBS to each well.	PBS (500 – N_{wells} mL)<br>Waste	Tissue Culture Lab (Grade B)	BSC<br>Aspirator<br>P1000 pipette
Step 3.21	Day -x (10 minutes)	TrypLE (1 x 100 mL)<br>Aspirator straw (x1)<br>P1000 pipette (x 1box)	**Cell harvest**<br>Using an aspirator, carefully remove the PBS by tilting the flask.<br>Using a 10 mL stripette, add 1 mL of TrypLE to each well.<br>Incubate for 3 minutes in the incubator.	TrypLE (100 – N_{wells} mL)<br>Waste	Tissue Culture Lab (Grade B)	BSC<br>Aspirator<br>Pipette boy<br>Incubator
Step 3.22	Day -x (5 minutes)		Visual inspection at the microscope<br>Are cells rounded and loosely attached to the plate?<br>NO → Incubate for 2 minutes in the incubator. (return to visual inspection)<br>YES → next step		Tissue Culture Lab (Grade B)	Microscope<br>Incubator

Day -x: Cell splitting

	Day and Duration	Materials in	Process Step	Materials Out	Location (Room and Grade)	Equipment
Step 3.23	Day -x (10 minutes)	Supplemented E8 media ($Vol_{E8media}$)<br>Aspirator straw (x1)<br>P1000 pipette tips (x1 box)<br>15 mL conical tube (x1)<br>P10 pipette tips (x 1 box)<br>1.5 mL Eppendorf tube (x1)	**Cell harvest**<br>Carefully tilt the plate and remove the TrypLE from the well by aspiration.<br>Using a p1000 pipette, add 1 mL supplemented E8 media and pipette on the surface until cells dissociate from the plate.<br>Using a p1000 pipette transfer the cell solution to a 15 mL conical tube.<br>Using a P10 pipette, transfer 10µL cells solution to a new 1.5 mL Eppendorf tube.	iPS cells in suspension (N_{wells} mL)<br>iPS cells aliquot for counting (10 µL)<br>Supplemented E8 media ($Vol_{E8media}$ - N_{wells})<br>Waste	Tissue Culture Lab (Grade B)	BSC<br>P10 pipette<br>P1000 pipette<br>Aspirator
Step 3.24	Day -x (10 minutes)	iPS cells aliquot for counting (10 µL)<br>Trypan Blue (1 mL)<br>P10 pipette tips (x1 box)	**Cell counting**<br>Using a p10 pipette, combine 10 µL cells with 10 µL Trypan Blue. Count cells using an haemocytometer<br><br>Is total cell number >8.00E07?<br>YES → Go to step 3.26 (Pre-differentiation seeding)<br>NO ↓	Total cell number N_{cell}<br>Waste	Tissue Culture Lab (Grade B)	Haemacytometer<br>P10 pipette<br>Microscope

Day -x: Cell splitting

	Day and Duration	Materials in	Process Step	Materials Out	Location (Room and Grade)	Equipment
Step 3.25	Day x<br>(20 minutes)	iPS cells in suspension (N_{wells} mL)<br>Vitronectin-coated 6-well plate (10 x N_{wells})<br>Supplemented E8 media ($Vol_{E8media}$ - N_{wells})<br>10 mL stripette (x1)<br>P1000 pipette tips (x1 box)<br>Aspirator straw (x1)	**Cell seeding**<br>For each passage, calculate the number of wells to be seeded as:<br><br>$N_{seed} = N_{harvest} \times 10$<br><br>Where $N_{harvest}$ is the number of wells from the previous passage.<br><br>Using an aspirator, remove the vitronectin from the vitronectin-coated 6-well plates. Using a 10 mL stripette, transfer 1.5 mL supplemented E8 media to each well. Using a P200 pipette, transfer 100µL cells solution to each well.<br><br>Place the plate in the incubator set at 37˚C, 5% CO_2. Mix the culture by cross-shaped movements to distribute cells evenly.<br><br>Return to Step 3.10 (repeat till required cell number is met)	iPS cells in 6-well plates (10 x N_{wells})<br>Waste	Tissue Culture Lab<br>(Grade B)	Calculator<br>BSC<br>P200 pipette<br>Pipette boy<br>Incubator<br>Aspirator

Day -1: Pre-differentiation seeding

	Day and Duration	Materials in	Process Step	Materials Out	Location (Room and Grade)	Equipment
Step 3.26	Day -1 (10 minutes)	Total cell number N_{cell}	**Calculation of the required volume of cells to be seeded** Based on the cell number, measured with the haemocytometer, calculate the volume of cells required to seed each well of a 6-well plate at 1.14 E05 cells per well:<br><br>Vol_{cells} (µL) = (114,000 x 10000 / N_{cell})	Cell Volume to be seeded Vol_{cell}		Calculator
Step 3.27	Day -1 (90 minutes)	iPS cells in suspension (1 x 120 mL)<br>Supplemented E8 media (3 x 400 mL)<br>Vitronectin-coated 6-well plates (x 94)<br>50 mL conical tube (x3)<br>10 mL stripette (x 94)<br>P200 pipette tips (x 9)<br>Cell Volume to be seeded Vol_{cell}	**Cell seeding** Seeding should occur in three parallel batches (by 3 operators) to avoid cells remaining outside of an incubator for an extended period. Swirl the cell suspension to mix well and transfer 40 mL into 3 x 50 mL conical tube. Transfer two aliquots of cells and medium to other two BSCs. Using an aspirator, remove the vitronectin from each vitronectin-coated 6-well plate. Using a 10 mL stripette, add 1.5 mL of E8 media to each well. Using a p200 pipette, add Vol_{cell} to each well.<br><br>Place the flask in the incubator set at 37˚C, 5% CO_2. Mix the culture by cross-shaped movements to distribute cells evenly.	iPS cells plated into vitronectin-coated 6-well plates (x94)<br>Waste	Tissue Culture (Grade B)	BSC (x3)<br>Pipette boy (x3)<br>P1000 pipette (x3)

Step	Day and Duration	Materials in	Process Step	Materials Out	Location (Room and Grade)	Equipment
Step 3.28	Day 0 (15 minutes)	70% IPA	**Reagent spray** Spray accurately all reagents before placing them in the BSC.		Tissue Culture Lab (Grade B)	BSC
Step 3.29	Day 0 (5 minutes)	E6 media bottle (3 x 500 mL)<br>FGF2 (25µg) (x 1 vial)<br>BMP4 (50 µg) (x 1 vial)<br>Doxycycline (50 mg) (x1 vial)	**Reagent preparation** Take out media bottle from fridge and place at room temperature for 30 minutes. Thaw FGF2, BMP4 and Dox at room temperature.	E6 media bottle (3 x 500 mL)<br>FGF2 (25µg) (x 1 vial)<br>BMP4 (50 µg) (x 1 vial)<br>Doxycycline (50 mg) (x1 vial)	Tissue Culture Lab (Grade B)	4-8°C Fridge<br>-20°C Freezer
Step 3.30	Day 0 (10 minutes)	HCl 1 M<br>Embryo Water (100 mL)<br>100 mL stripette (x1)<br>5 mL stripette (x1)<br>250 mL bottle (x1)	**10 mM HCl preparation** Using a 100 mL stripette, transfer 99 mL Embryo water to a 250 mL bottle. Using a 5 mL stripette, add 1 mL HCl 1M.	HCl 10 mM (100 mL)<br>Waste	Tissue Culture Lab (Grade B)	BSC<br>Pipet boy
Step 3.31	Day 0 (10 minutes)	FGF2 (25µg) (x 1 vial)<br>Embryo Water (100 mL)<br>5 mL stripette (x1)<br>15 mL conical tubes (x1)<br>P1000 pipette tips (1 box)	**FGF2 reconstitution** Using a 5 mL stripette, transfer 4 mL water into a 15 mL conical tubes. Using a p1000 pipette, add 1 mL water to the FGF2 vial and mix well to resuspend. Using a p1000 pipette transfer the FGF2 solution into the 15 mL tube containing water and mix well.	FGF2 5 µg/mL (1 x 5 mL)<br>Embryo Water (95 mL)<br>Waste	Tissue Culture (Grade B)	BSC<br>P1000 pipette<br>Pipet boy

Day 0: Mesoderm Media addition

Step	Day and Duration	Materials in	Process Step	Materials Out	Location (Room and Grade)	Equipment
Step 3.32	Day 0 (10 minutes)	BMP4 (50 µg) (x 1 vial)<br>Embryo Water (95 mL)<br>HCl 10 mM (100 mL)<br>5 mL stripette (x2)<br>15 mL conical tubes (x1)<br>P1000 pipette tips (1 box)	**BMP4 reconstitution** Using a 5 mL stripette, transfer 2 mL water and 2 mL HCl 10mM into a 15 mL conical tubes. Using a p1000 pipette, add 1 mL water to the BMP4 vial and mix well to resuspend. Using a p1000 pipette transfer the BMP4 solution into the 15 mL tube containing water and HCl and mix well.	BMP4 10 µg/mL (1 x 5 mL)<br>Embryo Water (90 mL)<br>Waste	Tissue Culture (Grade B)	BSC<br>P1000 pipette<br>Pipet boy
Step 3.33	Day 0 (10 minutes)	Doxycycline (50 mg) (x 1 vial)<br>Embryo Water (90 mL)<br>50 mL conical tube (x1)<br>P1000 pipette tips (1 box)<br>50 mL stripette (x1)	**Doxycycline reconstitution** Using a 50 mL stripette, transfer 49 mL water into a 50 mL conical tube. Using a p1000 pipette, add 1 mL water to the Doxycycline vial and mix well to resuspend. Using a p1000 pipette transfer the Doxycycline solution into the 50 mL tube containing water and mix well.	Doxycycline 1 mg/mL (1 x 50 mL)<br>Embryo Water (40 mL)<br>Waste	Tissue Culture (Grade B)	BSC<br>P1000 pipette<br>Pipet boy
Step 3.34	Day 0 (10 minutes)	E6 media (3 x 500 mL)<br>FGF2 5 µg/mL (1 x 5 mL)<br>BMP4 10 µg/mL (1 x 5 mL)<br>Dox aliquot 1 mg/mL (1 x 50 mL)<br>500 mL bottle (x3)<br>100 mL stripette (x3)<br>P1000 pipette tips (1 box)<br>P20 pipette tips (1 box)	**Mesoderm media preparation** Using a 100 mL stripette, transfer 400 mL E6 media into 3 x 500 mL bottle. Using a p1000 pipette, add 1600 µL FGF2 (20 ng/mL) and 400 µL BMP4 (10 ng/mL) into each bottle. Using a p20 pipette add 12.5 µL Dox (0.03125 µg/mL) into each bottle.	E6 media (300 mL)<br>Mesoderm media (3 x 400 mL)<br>Waste	Tissue Culture (Grade B)	BSC<br>P1000 pipette<br>P200 pipette<br>Pipette boy

	Day and Duration	Materials in	Process Step	Materials Out	Location (Room and Grade)	Equipment
Step 3.35	Day 0 (180 minutes)	Plated iPS cells (94 x 6-well plates)<br>Mesoderm media (3 x 400 mL)<br>Aspirator straw (x 94)<br>10 mL stripette (x 94)	**Media exchange**<br>Media exchange should occur in three parallel batches (by 3 operators). Using an aspirator, carefully remove old media from all wells of a 6-well plate by tilting the plate. Using a 10 mL stripette and tilting the plate, add 1.5 mL mesoderm media slowly to the edge of all wells in a single plate.<br><br>Place the plate in the incubator set at 37˚C, 5% CO_2.	Plated iPS cells in Mesoderm media (94 x 6-well plates)<br>Waste	Tissue Culture (Grade B)	BSC (x3)<br>Incubator<br>Aspirator (x3)<br>Pipette boy (x3)

Step	Day and Duration	Materials in	Process Step	Materials Out	Location (Room and Grade)	Equipment
Step 3.36	Day 1 (15 minutes)	70% IPA	**Reagent spray** Spray accurately all reagents before placing them in the BSC.		Tissue Culture Lab (Grade B)	BSC
Step 3.37	Day 1 (5 minutes)	E6 media bottle (3 x 500 mL)<br>FGF2 (25 µg) (x 1 vial)<br>BMP4 (50 µg) (x 1 vial)<br>Doxycycline (50 mg) (x1 vial)	**Reagent preparation** Take out media bottle from fridge and place at room temperature for 30 minutes. Thaw FGF2, BMP4 and Dox at room temperature.	E6 media bottle (3 x 500 mL)<br>FGF2 (25 µg) (x 1 vial)<br>BMP4 (50 µg) (x 1 vial)<br>Doxycycline (50 mg) (x1 vial)	Tissue Culture Lab (Grade B)	4-8°C Fridge<br>-20°C Freezer
Step 3.38	Day 1 (10 minutes)	HCl 1 M<br>Embryo Water (100 mL)<br>100 mL stripette (x1)<br>5 mL stripette (x1)<br>250 mL bottle (x1)	**10 mM HCl preparation** Using a 100 mL stripette, transfer 99 mL Embryo water to a 250 mL bottle. Using a 5 mL stripette, add 1 mL HCl 1M.	HCl 10 mM (100 mL)<br>Waste	Tissue Culture Lab (Grade B)	BSC<br>Pipet boy
Step 3.39	Day 1 (10 minutes)	FGF2 (25 µg) (x 1 vial)<br>Embryo Water (100 mL)<br>5 mL stripette (x1)<br>15 mL conical tubes (x1)<br>P1000 pipette tips (1 box)	**FGF2 reconstitution** Using a 5 mL stripette, transfer 4 mL water into a 15 mL conical tubes. Using a p1000 pipette, add 1 mL water to the FGF2 vial and mix well to resuspend. Using a p1000 pipette transfer the FGF2 solution into the 15 mL tube containing water and mix well.	FGF2 5 µg/mL (1 x 5 mL)<br>Embryo Water (95 mL)<br>Waste	Tissue Culture (Grade B)	BSC<br>P1000 pipette<br>Pipet boy

Step	Day and Duration	Materials in	Process Step	Materials Out	Location (Room and Grade)	Equipment
Step 3.40	Day 1 (10 minutes)	BMP4 (50 µg) (x 1 vial)<br>Embryo Water (95 mL)<br>HCl 10 mM (100 mL)<br>5 mL stripette (x2)<br>15 mL conical tubes (x1)<br>P1000 pipette tips (1 box)	**BMP4 reconstitution**<br>Using a 5 mL stripette, transfer 2 mL water and 2 mL HCl 10mM into a 15 mL conical tubes. Using a p1000 pipette, add 1 mL water to the BMP4 vial and mix well to resuspend. Using a p1000 pipette transfer the BMP4 solution into the 15 mL tube containing water and HCl and mix well.	BMP4 10 µg/mL (1 x 5 mL)<br>Embryo Water (90 mL)<br>Waste	Tissue Culture (Grade B)	BSC<br>P1000 pipette<br>Pipet boy
Step 3.41	Day 1 (10 minutes)	Doxycycline (50 mg) (x 1 vial)<br>Embryo Water (90 mL)<br>50 mL conical tube (x1)<br>P1000 pipette tips (1 box)<br>50 mL stripette (x1)	**Doxycycline reconstitution**<br>Using a 50 mL stripette, transfer 49 mL water into a 50 mL conical tube. Using a p1000 pipette, add 1 mL water to the Doxycycline vial and mix well to resuspend. Using a p1000 pipette transfer the Doxycycline solution into the 50 mL tube containing water and mix well.	Doxycycline 1 mg/mL (1 x 50 mL)<br>Embryo Water (40 mL)<br>Waste	Tissue Culture (Grade B)	BSC<br>P1000 pipette<br>Pipet boy
Step 3.42	Day 1 (10 minutes)	CHIR99021 (10 nM) (x1 vial)<br>DMSO (100 mL)<br>5 mL stripette (x1)<br>15 mL conical tubes (x1)<br>P1000 pipette tips (1 box)	**CHIR99021 dilution**<br>Using a 5 mL stripette, transfer 3.5 mL DMSO into a 15 mL conical tube. Using a p1000 pipette, add 1500 µL CHIR99021 into the tube and mix well to resuspend.	CHIR99021 3 mM (5 mL)<br>DMSO (96.5 mL)<br>Waste	Tissue Culture (Grade B)	BSC<br>P1000 pipette

Day 1: Mesoderm Media exchange

	Day and Duration	Materials in	Process Step	Materials Out	Location (Room and Grade)	Equipment
Step 3.43	Day 1 (10 minutes)	E6 media (3 x 500 mL)<br>FGF2 5 µg/mL (1 x 5 mL)<br>BMP4 10 µg/mL (1 x 5 mL)<br>Dox aliquot 1 mg/mL (1 x 50 mL)<br>CHIR99021 3 mM (1 x 5 mL)<br>500 mL bottle (x3)<br>100 mL stripette (x3)<br>P1000 pipette tips (1 box)<br>P20 pipette tips (1 box)	**Mesoderm media preparation**<br>Using a 100 mL stripette, transfer 400 mL E6 media into 3 x 500 mL bottle. Using a p1000 pipette, add 1600 µL FGF2 (20 ng/mL), 400 µL BMP4 (10 ng/mL) and 400 µL CHIR99021 into each bottle. Using a p20 pipette add 12.5 µL Dox (0.03125 µg/mL) into each bottle.	E6 media (300 mL)<br>Mesoderm media (3 x 400 mL)<br>Waste	Tissue Culture (Grade B)	BSC<br>P1000 pipette<br>P200 pipette<br>Pipette boy
Step 3.44	Day 1 (180 minutes)	Plated iPS cells (94 x 6-well plates)<br>Mesoderm media (3 x 400 mL)<br>Aspirator straw (x 94)<br>10 mL stripette (x 94)	**Media exchange**<br>Media exchange should occur in three parallel batches (by 3 operators). Using an aspirator, carefully remove old media from all wells of a 6-well plate by tilting the plate. Using a 10 mL stripette and tilting the plate, add 1.5 mL mesoderm media slowly to the edge of all wells in a single plate.<br><br>Place the plate in the incubator set at 37˚C, 5% CO_2.	Plated iPS cells in Mesoderm media (94 x 6-well plates)<br>Waste	Tissue Culture (Grade B)	BSC (x3)<br>Incubator<br>Aspirator (x3)<br>Pipette boy (x3)

Day 2: Mesoderm Media exchange

Step	Day and Duration	Materials in	Process Step	Materials Out	Location (Room and Grade)	Equipment
Step 3.45	Day 2 (15 minutes)	70% IPA	**Reagent spray** Spray accurately all reagents before placing them in the BSC.		Tissue Culture Lab (Grade B)	BSC
Step 3.46	Day 2 (5 minutes)	E6 media bottle (3 x 500 mL)<br>FGF2 (25 µg) (x 1 vial)<br>BMP4 (50 µg) (x 1 vial)<br>Doxycycline (50 mg) (x1 vial)	**Reagent preparation** Take out media bottle from fridge and place at room temperature for 30 minutes. Thaw FGF2, BMP4 and Dox at room temperature.	E6 media bottle (3 x 500 mL)<br>FGF2 (25µg) (x 1 vial)<br>BMP4 (50 µg) (x 1 vial)<br>Doxycycline (50 mg) (x1 vial)	Tissue Culture Lab (Grade B)	4-8°C Fridge<br>-20°C Freezer
Step 3.47	Day 2 (10 minutes)	HCl 1 M<br>Embryo Water (100 mL)<br>100 mL stripette (x1)<br>5 mL stripette (x1)<br>250 mL bottle (x1)	**10 mM HCl preparation** Using a 100 mL stripette, transfer 99 mL Embryo water to a 250 mL bottle. Using a 5 mL stripette, add 1 mL HCl 1M.	HCl 10 mM (100 mL)<br>Waste	Tissue Culture Lab (Grade B)	BSC<br>Pipet boy
Step 3.48	Day 2 (10 minutes)	FGF2 (25 µg) (x 1 vial)<br>Embryo Water (100 mL)<br>5 mL stripette (x1)<br>15 mL conical tubes (x1)<br>P1000 pipette tips (1 box)	**FGF2 reconstitution** Using a 5 mL stripette, transfer 4 mL water into a 15 mL conical tubes. Using a p1000 pipette, add 1 mL water to the FGF2 vial and mix well to resuspend. Using a p1000 pipette transfer the FGF2 solution into the 15 mL tube containing water and mix well.	FGF2 5 µg/mL (1 x 5 mL)<br>Embryo Water (95 mL)<br>Waste	Tissue Culture (Grade B)	BSC<br>P1000 pipette<br>Pipet boy

Step	Day and Duration	Materials in	Process Step	Materials Out	Location (Room and Grade)	Equipment
Step 3.49	Day 2 (10 minutes)	BMP4 (50 µg) (x 1 vial)<br>Embryo Water (95 mL)<br>HCl 10 mM (100 mL)<br>5 mL stripette (x2)<br>15 mL conical tubes (x1)<br>P1000 pipette tips (1 box)	**BMP4 reconstitution** Using a 5 mL stripette, transfer 2 mL water and 2 mL HCl 10mM into a 15 mL conical tubes. Using a p1000 pipette, add 1 mL water to the BMP4 vial and mix well to resuspend. Using a p1000 pipette transfer the BMP4 solution into the 15 mL tube containing water and HCl and mix well.	BMP4 10 µg/mL (1 x 5 mL)<br>Embryo Water (90 mL)<br>Waste	Tissue Culture (Grade B)	BSC<br>P1000 pipette<br>Pipet boy
Step 3.50	Day 2 (10 minutes)	Doxycycline (50 mg) (x 1 vial)<br>Embryo Water (90 mL)<br>50 mL conical tube (x1)<br>P1000 pipette tips (1 box)<br>50 mL stripette (x1)	**Doxycycline reconstitution** Using a 50 mL stripette, transfer 49 mL water into a 50 mL conical tube. Using a p1000 pipette, add 1 mL water to the Doxycycline vial and mix well to resuspend. Using a p1000 pipette transfer the Doxycycline solution into the 50 mL tube containing water and mix well.	Doxycycline 1 mg/mL (1 x 50 mL)<br>Embryo Water (40 mL)<br>Waste	Tissue Culture (Grade B)	BSC<br>P1000 pipette<br>Pipet boy
Step 3.51	Day 2 (10 minutes)	E6 media (3 x 500 mL)<br>FGF2 5 µg/mL (1 x 5 mL)<br>BMP4 10 µg/mL (1 x 5 mL)<br>Dox aliquot 1 mg/mL (1 x 50 mL)<br>500 mL bottle (x3)<br>100 mL stripette (x3)<br>P1000 pipette tips (1 box)<br>P20 pipette tips (1 box)	**Mesoderm media preparation** Using a 100 mL stripette, transfer 400 mL E6 media into 3 x 500 mL bottle. Using a p1000 pipette, add 1600 µL FGF2 (20 ng/mL) and 400 µL BMP4 (10 ng/mL) into each bottle. Using a p20 pipette add 12.5 µL Dox (0.03125 µg/mL) into each bottle.	E6 media (300 mL)<br>Mesoderm media (3 x 400 mL)<br>Waste	Tissue Culture (Grade B)	BSC<br>P1000 pipette<br>P200 pipette<br>Pipette boy

Day 2: Mesoderm Media exchange

	Day and Duration	Materials in	Process Step	Materials Out	Location (Room and Grade)	Equipment
Step 3.52	Day 2 (180 minutes)	Plated iPS cells (94 x 6-well plates)<br>Mesoderm media (3 x 400 mL)<br>Aspirator straw (x 94)<br>10 mL stripette (x 94)	**Media exchange**<br>Media exchange should occur in three parallel batches (by 3 operators). Using an aspirator, carefully remove old media from all wells of a 6-well plate by tilting the plate. Using a 10 mL stripette and tilting the plate, add 1.5 mL mesoderm media slowly to the edge of all wells in a single plate.<br><br>Place the plate in the incubator set at 37˚C, 5% CO_2.	Plated iPS cells in Mesoderm media (94 x 6-well plates)<br>Waste	Tissue Culture (Grade B)	BSC (x3)<br>Incubator<br>Aspirator (x3)<br>Pipette boy (x3)

Step	Day and Duration	Materials in	Process Step	Materials Out	Location (Room and Grade)	Equipment
Step 3.53	Day 3 (15 minutes)	70% IPA	**Reagent spray** Spray accurately all reagents before placing them in the BSC.		Tissue Culture Lab (Grade B)	BSC
Step 3.54	Day 3 (5 minutes)	IMDM media (3 x 500 mL)<br>TPO (50 µg) (x1 vial)<br>SCF (100 µg) (x1 vials)<br>Doxycycline (50 mg) (x1 vial)<br>10% HSA (1 x 100 mL)<br>ITS 100X (2 x 10 mL)	**Reagent preparation** Take out media bottle and ITS from fridge and place at room temperature for 30 minutes. Thaw TPO, BMP4, Dox and 10% HSA at room temperature.	IMDM media (3 x 500 mL)<br>TPO (50 µg) (x1 vial)<br>SCF (100 µg) (x1 vials)<br>Doxycycline (50 mg) (x1 vial)<br>10% HSA (1 x 100 mL)<br>ITS 100X (2 x 10 mL)	Tissue Culture Lab (Grade B)	4-8°C Fridge<br>-20°C Freezer
Step 3.55	Day 3 (10 minutes)	TPO (50 µg) (x1 vial)<br>Embryo Water (100 mL)<br>15 mL conical tube (x1)<br>P1000 pipette tips (1 box)<br>5 mL stripette (x1)	**TPO reconstitution** Using a 5 mL stripette, transfer 4 mL water into a 15 mL conical tubes. Using a p1000 pipette, add 1 mL water to the TPO vial and mix well to resuspend. Using a p1000 pipette transfer the TPO solution into the 15 mL tube containing water and mix well.	TPO 10 µg/mL (1 x 5 mL)<br>Embryo Water (95 mL)<br>Waste	Tissue Culture (Grade B)	BSC<br>P1000 pipette<br>Pipet boy
Step 3.56	Day 3 (10 minutes)	SCF (100 µg) (x1 vials)<br>Embryo Water (95 mL)<br>5 mL stripette (x1)<br>15 mL conical tubes (x1)<br>P1000 pipette tips (1 box)	**SCF reconstitution** Using a 5 mL stripette, transfer 1 mL water a 15 mL conical tube. Using a p1000 pipette, add 1 mL water to the SCF vial and mix well to resuspend. Using a p1000 pipette transfer the SCF solution into the 15 mL tube containing water and mix well.	SCF 50 µg/mL (1 x 2 mL)<br>Embryo Water (93 mL)<br>Waste	Tissue Culture (Grade B)	BSC<br>P1000 pipette<br>Pipet boy

	Day and Duration	Materials in	Process Step	Materials Out	Location (Room and Grade)	Equipment
Step 3.57	Day 3 (10 minutes)	Doxycycline (50 mg) (x 1 vial)<br>Embryo Water (93 mL)<br>50 mL conical tube (x1)<br>P1000 pipette tips (1 box)<br>50 mL stripette (x1)	**Doxycycline reconstitution** Using a 50 mL stripette, transfer 49 mL water into a 50 mL conical tube. Using a p1000 pipette, add 1 mL water to the Doxycycline vial and mix well to resuspend. Using a p1000 pipette transfer the Doxycycline solution into the 50 mL tube containing water and mix well.	Doxycycline 1 mg/mL (1 x 50 mL)<br>Embryo Water (43 mL)<br>Waste	Tissue Culture (Grade B)	BSC<br>P1000 pipette<br>Pipet boy
Step 3.58	Day 3 (15 minutes)	IMDM media (3 x 500 mL)<br>10% HSA (1 x 100 mL)<br>ITS 100X (2 x 10 mL)<br>Beta-mercaptoethanol 55 mM (50mL)<br>TPO 10 µg/mL (1 x 5 mL)<br>SCF 50 µg/mL (1 x 2 mL)<br>Dox 1 mg/mL (1 x 50 mL)<br>100 mL stripette (x6)<br>25 mL stripette (x3)<br>5 mL stripette (x3)<br>P1000 pipette tips (x1 box)<br>500 mL bottle (x3)<br>P20 pipette tips (x1 box)	**MK media preparation** Using a 100 mL stripette, add 376 mL IMDM media to 3 x 500 mL bottles. Using a 25 mL stripette, add 20 mL 10% HSA. Using a 5 mL pipette, add 4 mL ITS 100X. Using a p1000 pipette, add 364 µL of Beta-mercaptoethanol to each bottle. With a 100 mL stripette mix well. Using a p1000 pipette, add 800 µL TPO (20 ng/mL) and 200 µL SCF (25 ng/mL) into each bottle. Using a p20 pipette add 12.5 µL Dox (0.03125 µg/mL).	MK media (3 x 400 mL)<br>10% HSA (40 mL)<br>ITS 100X (8 mL)<br>Beta-mercaptoethanol 55 mM (49 mL)<br>Waste	Tissue Culture (Grade B)	BSC<br>Pipet boy<br>P1000 pipette<br>P20 pipette

	Day and Duration	Materials in	Process Step	Materials Out	Location (Room and Grade)	Equipment
Step 3.59	Day 3 (180 minutes)	Plated iPS cells (94 x 6-well plates)<br>MK media (3 x 400 mL)<br>Aspirator straw (x 94)<br>10 mL stripette (x 94)	**Media exchange**<br>Media exchange should occur in three parallel batches (by 3 operators). Using an aspirator, carefully remove old media from all wells of a 6-well plate by tilting the plate. Using a 10 mL stripette and tilting the plate, add 1.5 mL MK media slowly to the edge of all wells in a single plate.<br><br>Place the plate in the incubator set at 37˚C, 5% CO_2.	Plated iPS cells in MK media (94 x 6-well plates)<br>Waste	Tissue Culture (Grade B)	BSC (x3)<br>Incubator<br>Aspirator (x3)<br>Pipette boy (x3)

Step	Day and Duration	Materials in	Process Step	Materials Out	Location (Room and Grade)	Equipment
Step 3.60	Day 5/7 (15 minutes)	70% IPA	**Reagent spray** Spray accurately all reagents before placing them in the BSC.		Tissue Culture Lab (Grade B)	BSC
Step 3.61	Day 5/7 (5 minutes)	IMDM media (4 x 500 mL)<br>TPO (50 µg) (x2 vial)<br>SCF (100 µg) (x2 vials)<br>Doxycycline (50 mg) (x2 vial)<br>10% HSA (2 x 100 mL)<br>ITS 100X (2 x 10 mL)	**Reagent preparation** Take out media bottle and ITS from fridge and place at room temperature for 30 minutes. Thaw TPO, BMP4, Dox and 10% HSA at room temperature.	IMDM media (4 x 500 mL)<br>TPO (50 µg) (x2 vial)<br>SCF (100 µg) (x2 vials)<br>Doxycycline (50 mg) (x2 vial)<br>10% HSA (2 x 100 mL)<br>ITS 100X (2 x 10 mL)	Tissue Culture Lab (Grade B)	4-8°C Fridge<br>-20°C Freezer
Step 3.62	Day 5/7 (10 minutes)	TPO (50 µg) (x2 vial)<br>Embryo Water (2 x 100 mL)<br>15 mL conical tube (x2)<br>P1000 pipette tips (2 x 1 box)<br>5 mL stripette (x2)	**TPO reconstitution** Using a 5 mL stripette, transfer 4 mL water into a 15 mL conical tubes. Using a p1000 pipette, add 1 mL water to the TPO vial and mix well to resuspend. Using a p1000 pipette transfer the TPO solution into the 15 mL tube containing water and mix well.	TPO 10 µg/mL (2 x 5 mL)<br>Embryo Water (2 x 95 mL)<br>Waste	Tissue Culture (Grade B)	BSC<br>P1000 pipette<br>Pipet boy
Step 3.63	Day 5/7 (10 minutes)	SCF (100 µg) (x2 vials)<br>Embryo Water (2 x 95 mL)<br>5 mL stripette (x2)<br>15 mL conical tubes (x2)<br>P1000 pipette tips (2 x 1 box)	**SCF reconstitution** Using a 5 mL stripette, transfer 1 mL water a 15 mL conical tube. Using a p1000 pipette, add 1 mL water to the SCF vial and mix well to resuspend. Using a p1000 pipette transfer the SCF solution into the 15 mL tube containing water and mix well.	SCF 50 µg/mL (2 x 2 mL)<br>Embryo Water (2 x 93 mL)<br>Waste	Tissue Culture (Grade B)	BSC<br>P1000 pipette<br>Pipet boy

Step	Day and Duration	Materials in	Process Step	Materials Out	Location (Room and Grade)	Equipment
Step 3.64	Day 5/7 (10 minutes)	Doxycycline (50 mg) (x 2 vial)<br>Embryo Water (2 x 93 mL)<br>50 mL conical tube (x2)<br>P1000 pipette tips (2 x 1 box)<br>50 mL stripette (x2)	**Doxycycline reconstitution**<br>Using a 50 mL stripette, transfer 49 mL water into a 50 mL conical tube. Using a p1000 pipette, add 1 mL water to the Doxycycline vial and mix well to resuspend. Using a p1000 pipette transfer the Doxycycline solution into the 50 mL tube containing water and mix well.	Doxycycline 1 mg/mL (2 x 50 mL)<br>Embryo Water (2 x 43 mL)<br>Waste	Tissue Culture (Grade B)	BSC<br>P1000 pipette<br>Pipet boy
Step 3.65	Day 5/7 (15 minutes)	IMDM media (4 x 500 mL)<br>10% HSA (2 x 100 mL)<br>ITS 100X (2 x 10 mL)<br>Beta-mercaptoethanol 55 mM (2 x 50mL)<br>TPO 10 µg/mL (2 x 5 mL)<br>SCF 50 µg/mL (2 x 2 mL)<br>Dox 1 mg/mL (2 x 50 mL)<br>100 mL stripette (x12)<br>25 mL stripette (x6)<br>5 mL stripette (x6)<br>P1000 pipette tips (x2 box)<br>250 mL bottle (x6)<br>P20 pipette tips (x2 box)	**2x MK media preparation**<br>Using a 100 mL stripette, add 235 mL IMDM media to 3 x 250 mL bottles. Using a 25 mL stripette, add 12.5 mL 10% HSA. Using a 5 mL pipette, add 2.5 mL ITS 100X. Using a p1000 pipette, add 227 µL of Beta-mercaptoethanol to each bottle. With a 100 mL stripette mix well. Using a p1000 pipette, add 1000 µL TPO (40 ng/mL) and 250 µL SCF (50 ng/mL) into each bottle. Using a p20 pipette add 15.6 µL Dox (0.0625 µg/mL).	2 x MK media (6 x 250 mL)<br>10% HSA (2 x 62.5 mL)<br>ITS 100X (2 x 2.5 mL)<br>Beta-mercaptoethanol 55 mM (2 x 49 mL)<br>Waste	Tissue Culture (Grade B)	BSC<br>Pipet boy<br>P1000 pipette<br>P20 pipette

Day 5/7: 2x MK media exchange

	Day and Duration	Materials in	Process Step	Materials Out	Location (Room and Grade)	Equipment
Step 3.66	Day 5/7 (180 minutes)	Plated iPS cells (94 x 6-well plates)<br>MK media (6 x 250 mL)<br>P1000 pipette tips (2 x 15 box)	**Media exchange**<br>Media exchange should occur in three parallel batches (by 3 operators). Using a P1000 pipette, carefully remove 0.8 mL of old media by tilting the plate. Using a P1000 pipette and tilting the plate, add 1 mL 2X MK media slowly to the edge.<br><br>Place the plate in the incubator set at 37˚C, 5% CO_2.	Plated iPS cells in MK media (94 x 6-well plates)<br>Waste	Tissue Culture (Grade B)	BSC (x3)<br>Incubator<br>P1000 pipette (x3)

Day 9: 3x MK media addition

Step	Day and Duration	Materials in	Process Step	Materials Out	Location (Room and Grade)	Equipment
Step 3.67	Day 9 (15 minutes)	70% IPA	**Reagent spray** Spray accurately all reagents before placing them in the BSC.		Tissue Culture Lab (Grade B)	BSC
Step 3.68	Day 9 (5 minutes)	IMDM media (2 x 500 mL)<br>TPO (50 µg) (x1 vial)<br>SCF (100 µg) (x1 vials)<br>Doxycycline (50 mg) (x1 vial)<br>10% HSA (1 x 100 mL)<br>ITS 100X (1 x 10 mL)	**Reagent preparation** Take out media bottle and ITS from fridge and place at room temperature for 30 minutes. Thaw TPO, BMP4, Dox and 10% HSA at room temperature.	IMDM media (2 x 500 mL)<br>TPO (50 µg) (x1 vial)<br>SCF (100 µg) (x1 vials)<br>Doxycycline (50 mg) (x1 vial)<br>10% HSA (1 x 100 mL)<br>ITS 100X (1 x 10 mL)	Tissue Culture Lab (Grade B)	4-8°C Fridge<br>-20°C Freezer
Step 3.69	Day 9 (10 minutes)	TPO (50 µg) (x1 vial)<br>Embryo Water (1 x 100 mL)<br>15 mL conical tube (x1)<br>P1000 pipette tips (x 1 box)<br>5 mL stripette (x1)	**TPO reconstitution** Using a 5 mL stripette, transfer 4 mL water into a 15 mL conical tubes. Using a p1000 pipette, add 1 mL water to the TPO vial and mix well to resuspend. Using a p1000 pipette transfer the TPO solution into the 15 mL tube containing water and mix well.	TPO 10 µg/mL (1 x 5 mL)<br>Embryo Water (1 x 95 mL)<br>Waste	Tissue Culture (Grade B)	BSC<br>P1000 pipette<br>Pipet boy
Step 3.70	Day 9 (10 minutes)	SCF (100 µg) (x1 vials)<br>Embryo Water (1 x 95 mL)<br>5 mL stripette (x1)<br>15 mL conical tubes (x1)<br>P1000 pipette tips (x 1 box)	**SCF reconstitution** Using a 5 mL stripette, transfer 1 mL water a 15 mL conical tube. Using a p1000 pipette, add 1 mL water to the SCF vial and mix well to resuspend. Using a p1000 pipette transfer the SCF solution into the 15 mL tube containing water and mix well.	SCF 50 µg/mL (1 x 2 mL)<br>Embryo Water (1 x 93 mL)<br>Waste	Tissue Culture (Grade B)	BSC<br>P1000 pipette<br>Pipet boy

Step	Day and Duration	Materials in	Process Step	Materials Out	Location (Room and Grade)	Equipment
Step 3.71	Day 9 (10 minutes)	Doxycycline (50 mg) (x 1 vial); Embryo Water (1 x 93 mL); 50 mL conical tube (x1); P1000 pipette tips (x 1 box); 50 mL stripette (x1)	**Doxycycline reconstitution** Using a 50 mL stripette, transfer 49 mL water into a 50 mL conical tube. Using a p1000 pipette, add 1 mL water to the Doxycycline vial and mix well to resuspend. Using a p1000 pipette transfer the Doxycycline solution into the 50 mL tube containing water and mix well.	Doxycycline 1 mg/mL (1 x 50 mL); Embryo Water (1 x 43 mL); Waste	Tissue Culture (Grade B)	BSC; P1000 pipette; Pipet boy
Step 3.72	Day 9 (15 minutes)	IMDM media (2 x 500 mL); 10% HSA (1 x 100 mL); ITS 100X (1 x 10 mL); Beta-mercaptoethanol 55 mM (1 x 50mL); TPO 10 µg/mL (1 x 5 mL); SCF 50 µg/mL (1 x 2 mL); Dox 1 mg/mL (1 x 50 mL); 100 mL stripette (x6); 25 mL stripette (x3); 5 mL stripette (x3); P1000 pipette tips (x1 box); 250 mL bottle (x3); P200 pipette tips (x1 box)	**3x MK media preparation** Using a 100 mL stripette, add 235 mL IMDM media to 3 x 250 mL bottles. Using a 25 mL stripette, add 12.5 mL 10% HSA. Using a 5 mL pipette, add 2.5 mL ITS 100X. Using a p1000 pipette, add 227 µL of Beta-mercaptoethanol to each bottle. With a 100 mL stripette mix well. Using a p1000 pipette, add 1500 µL TPO (60 ng/mL) and 375 µL SCF (75 ng/mL) into each bottle. Using a p200 pipette add 23.4 µL Dox (0.0625 µg/ mL).	3x MK media (3 x 250 mL); 10% HSA (1 x 62.5 mL); ITS 100X (1 x 2.5 mL); Beta-mercaptoethanol 55 mM (1 x 49 mL); Waste	Tissue Culture (Grade B)	BSC; Pipet boy; P1000 pipette; P200 pipette

Day 9: 3x MK media addition

Step	Day and Duration	Materials in	Process Step	Materials Out	Location (Room and Grade)	Equipment
Step 3.73	Day 9 (180 minutes)	Plated iPS cells (94 x 6-well plates)<br>MK media (3 x 250 mL)<br>10 mL stripette (x 94)	**Media top-up**<br>Media additon should occur in three parallel batches (by 3 operators). Using a 10 mL stripette and tilting the plate, add 1 mL 3X MK media slowly to the edge.<br><br>Place the plate in the incubator set at 37˚C, 5% CO_2.	Plated iPS cells in MK media (94 x 6-well plates)<br>Waste	Tissue Culture (Grade B)	BSC (x3)<br>Incubator<br>Pipette boy (x3)

Step	Day and Duration	Materials in	Process Step	Materials Out	Location (Room and Grade)	Equipment
Step 3.74	Day 10 (15 minutes)	70% IPA	**Reagent spray** Spray accurately all reagents before placing them in the BSC.		Tissue Culture Lab (Grade B)	BSC
Step 3.75	Day 10 (5 minutes)	IMDM media (7 x 500 mL)<br>TPO (50 µg) (x 1 vial)<br>SCF (100 µg) (x 1 vial)<br>Doxycycline (50 mg) (x1 vial)<br>10% HSA (2 x 100 mL)<br>ITS 100X (3 x 10 mL)	**Reagent preparation** Take out media bottle and ITS from fridge and place at room temperature for 30 minutes. Thaw TPO, BMP4, Dox and 10% HSA at room temperature.	IMDM media (5 x 500 mL)<br>TPO (50 µg) (x 1 vial)<br>SCF (100 µg) (x 1 vial)<br>Doxycycline (50 mg) (x1 vial)<br>10% HSA (2 x 100 mL)<br>ITS 100X (3 x 10 mL)	Tissue Culture Lab (Grade B)	4-8°C Fridge<br>-20°C Freezer
Step 3.76	Day 10 (10 minutes)	TPO (50 µg) (x1 vial)<br>Embryo Water (100 mL)<br>15 mL conical tube (x1)<br>P1000 pipette tips (1 box)<br>5 mL stripette (x1)	**TPO reconstitution** Using a 5 mL stripette, transfer 4 mL water into a 15 mL conical tubes. Using a p1000 pipette, add 1 mL water to the TPO vial and mix well to resuspend. Using a p1000 pipette transfer the TPO solution into the 15 mL tube containing water and mix well.	TPO 10 µg/mL (1 x 5 mL)<br>Embryo Water (95 mL)<br>Waste	Tissue Culture (Grade B)	BSC<br>P1000 pipette<br>Pipet boy
Step 3.77	Day 10 (10 minutes)	SCF (100 µg) (x1 vials)<br>Embryo Water (95 mL)<br>5 mL stripette (x1)<br>15 mL conical tubes (x1)<br>P1000 pipette tips (1 box)	**SCF reconstitution** Using a 5 mL stripette, transfer 1 mL water a 15 mL conical tube. Using a p1000 pipette, add 1 mL water to the SCF vial and mix well to resuspend. Using a p1000 pipette transfer the SCF solution into the 15 mL tube containing water and mix well.	SCF 50 µg/mL (1 x 2 mL)<br>Embryo Water (93 mL)<br>Waste	Tissue Culture (Grade B)	BSC<br>P1000 pipette<br>Pipet boy

Step	Day and Duration	Materials in	Process Step	Materials Out	Location (Room and Grade)	Equipment
Step 3.78	Day 10<br>(10 minutes)	Doxycycline (50 mg) (x 1 vial)<br>Embryo Water (93 mL)<br>50 mL conical tube (x1)<br>P1000 pipette tips (1 box)<br>50 mL stripette (x1)	**Doxycycline reconstitution**<br>Using a 50 mL stripette, transfer 49 mL water into a 50 mL conical tube. Using a p1000 pipette, add 1 mL water to the Doxycycline vial and mix well to resuspend. Using a p1000 pipette transfer the Doxycycline solution into the 50 mL tube containing water and mix well.	Doxycycline 1 mg/mL (1 x 50 mL)<br>Embryo Water (43 mL)<br>Waste	Tissue Culture (Grade B)	BSC<br>P1000 pipette<br>Pipet boy
Step 3.79	Day 10<br>(15 minutes)	IMDM media (5 x 500 mL)<br>10% HSA (2 x 100 mL)<br>ITS 100X (3 x 10 mL)<br>Beta-mercaptoethanol 55 mM (50mL)<br>TPO 10 µg/mL (1 x 5 mL)<br>SCF 50 µg/mL (1 x 2 mL)<br>Dox 1 mg/mL (1 x 50 mL)<br>100 mL stripette (x6)<br>50 mL stripette (x3)<br>10 mL stripette (x3)<br>P1000 pipette tips (x1 box)<br>1 L bottle (x3)<br>P200 pipette tips (x1 box)	**MK media preparation**<br>Using a 100 mL stripette, add 752 IMDM media to 3 x 1 L bottles. Using a 50 mL stripette, add 40 mL 10% HSA. Using a 10 mL pipette, add 8 mL ITS 100X. Using a p1000 pipette, add 728 µL of Beta-mercaptoethanol to each bottle. With a 100 mL stripette mix well. Using a p1000 pipette, add 1600 µL TPO (20 ng/mL) and 400 µL SCF (25 ng/mL) into each bottle. Using a p200 pipette add 25 µL Dox (0.03125 µg/ mL).	IMDM media (244 mL)<br>MK media (3 x 800 mL)<br>10% HSA (80 mL)<br>ITS 100X (6 mL)<br>Beta-mercaptoethanol 55 mM (49 mL)<br>Waste	Tissue Culture (Grade B)	BSC<br>Pipet boy<br>P1000 pipette<br>P200 pipette

D10: Cell Harvest and dissociation

	Day and Duration	Materials in	Process Step	Materials Out	Location (Room and Grade)	Equipment
Step 3.80	Day 10 (180 minutes)	IMDM media (2 x 500 mL)<br>Differentiated iPS cells (94 x 6-well plates)<br>TrypLe (8 x 100 mL)<br>10 mL stripette (x282)<br>100 mL stripette (x9)<br>500 mL conical Tube (x9)	**Cell collection**<br>Cell collection should occur in three parallel batches (by 3 operators). Add 100 mL of IMDM media to each conical tube using a 100 mL stripette. Using a 10 mL stripette collect the supernatant containing all the suspension cells (MK progenitors are usually in supernatant) from all wells of a 6-well plate and transfer to 9 x 500 mL conical centrifuge tubes (13 plates per tube). Using a 10 mL stripette, add 1 mL of TrypLe to each well and incubate for 10 minutes at 37° C. Using a 10 mL stripette, add 1.5 mL IMDM media and mix well to resuspend the cells. Transfer the TrypLe/Cell mix into the 500 mL conical centrifuge tube containing the cells in suspension.	MK cells in suspension (9 x 500 mL conical centrifuge tubes)<br>TrypLe (98 mL)<br>Waste	Tissue Culture (Grade B)	BSC x3<br>Incubator<br>Pipette boy (x3)

Day 10: Cell harvest and dissociation

Step	Day and Duration	Materials in	Process Step	Materials Out	Location (Room and Grade)	Equipment
Step 3.81	Day 10 (15 minutes)	MK cells in suspension (9x 500 mL conical centrifuge tubes)<br>Supplemented MK media (3 x 800 mL)<br>Aspirator straw (x9)<br>1.5 mL Eppendorf tubes (x4)<br>25 mL stripette (x9)<br>100 mL stripette (x1)<br>P200 pipette tips (x1 box)	**Cell pooling**<br>Centrifuge cells for 5 mins at 300g. Using an aspirator remove the supernatant. Using a 25 mL stripette, add 20 mL MK media to each of the 500 mL conical centrifuge tubes and resuspend the cells. Using a 100 mL pipette, mix the cell suspensions into a single 500 mL conical centrifuge tube. Using a p200 pipette, take 2 x 100 µL aliquots for counting and 2 x 100 µL aliquot for flow cytometry and transfer to 4 x 1.5 mL Eppendorf tubes.	MK cells in suspension (1x 180 mL)<br>MK cells aliquot for counting (2 x 100 µL)<br>MK cells aliquots for flow cytometry (2 x 100 µL)<br>Supplemented MK media (3 x 740 mL)<br>Waste	Tissue Culture (Grade B)	BSC<br>Pipette boy<br>Centrifuge<br>Aspirator<br>P200 pipette
Step 3.82	Day 10 (10 minutes)	MK cells aliquots for counting (2 x 100 µL)<br>Trypan Blue<br>P10 pipette tips (x1 box)	**Cell counting**<br>Mix 10 µL cell solution with 10 µL Trypan blue. Count cells using an haemocytometer	Total cell number N_{cell}<br>Waste	Tissue Culture (Grade B)	Haemacytometer<br>P10 pipette
Step 3.83	Day 10 (10 minutes)	Total cell number N_{cell}	**Evaluation of cell density**<br>Based on the cell number, measured with the haemocytometer, evaluate the cell density. If cell density is >1E06 cells/mL, split the cells into new culture vessels. In this study, at Day 10 cells will be seeded at 5E05 cells/mL into 188 x 6-well plates .	Number of plates to seed<br>Volume of cells per well V_{cell}		Calculator

	Day and Duration	Materials in	Process Step	Materials Out	Location (Room and Grade)	Equipment
Step 3.84	Day 10 (120 minutes)	MK cells in suspension (1x 180 mL)<br>Supplemented MK media (3 x 740 mL)<br>100 mL stripette (x1)<br>100 mL conical tube (x3)<br>10 mL stripette (x 188)<br>P200 pipette tips (15 x 1 box)	**Cell Seeding**<br>Cell seeding should occur in three parallel batches (by 3 operators). Using a 100 mL stripette, mix well the cell suspension and aliquot 60 mL into 3 x 100 mL conical tubes. Transfer each tube to a different BSC. Using a 10 mL stripette, transfer 1.5 mL MK media into each well of a 6-well plate. Using a p200 pipette, transfer V_{cells} of cell mix into each well.<br><br>Place the flask in the incubator set at 37˚C, 5% CO_2.	Plated MK cells into 6-well plates (x 188)<br>Waste	Tissue Culture (Grade B)	BSC (x3)<br>Incubator<br>Pipette boy (x3)<br>P200 pipette (x3)
Step 3.85	Day 10 (60 minutes)	MK cells aliquot for Flow Cytometry (2 x 100 µL)<br>Flow cytometry antibodies<br>P200 pipette tips (1 box)	**Flow Cytometry analysis**<br>Stain 100 µL for flow with CD235aPEcy7, CD42aAPC, CD41APCH7 antibodies. Incubate 20 minutes at RT in the dark. Wash with 500 µL flow buffer (PBS supplemented with 0.5% BSA and 2 mM EDTA). Centrifuge 100 x g, 8 minutes. Discard supernatant and resuspend in 300 µL PBE + Flow count beads (5000 beads, 5 uL) + 1 µg/mL DAPI. Run samples on a flow cytometer.	Flow cytometry data	Analytical lab	P200 pipette<br>Flow cytometer

D10: Cell Harvest and dissociation

	Day and Duration	Materials in	Process Step	Materials Out	Location (Room and Grade)	Equipment
Step 3.86		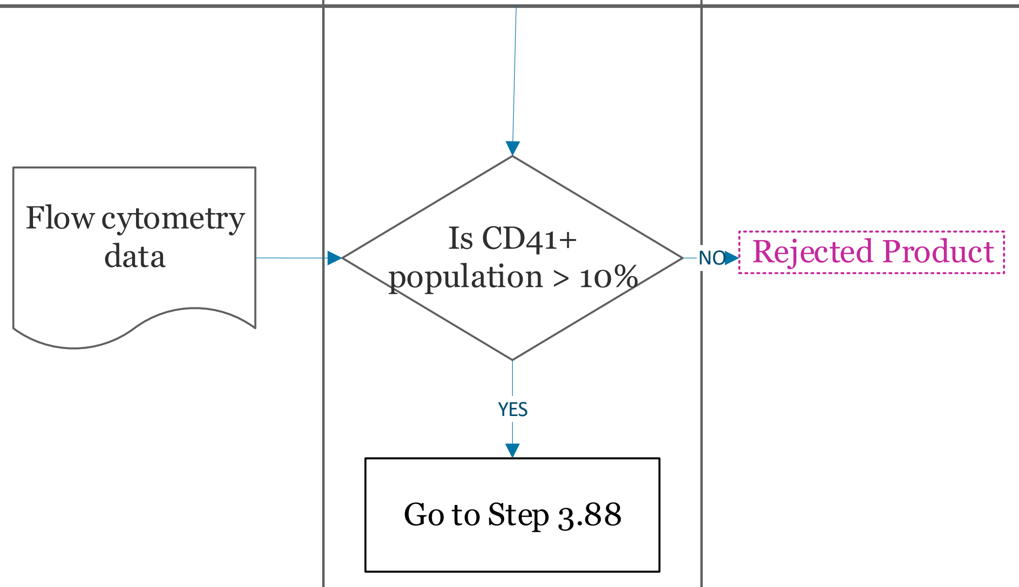				

Step	Day and Duration	Materials in	Process Step	Materials Out	Location (Room and Grade)	Equipment
Step 3.87	Day 12-18 (15 minutes)	70% IPA	**Reagent spray** Spray accurately all reagents before placing them in the BSC.		Tissue Culture Lab (Grade B)	BSC
Step 3.88	Day 12-18 (5 minutes)	IMDM media; TPO (50µg); SCF (100 µg); Doxycycline (50 mg); 10% HSA; ITS 100X	**Reagent preparation** Take out media bottle and ITS from fridge and place at room temperature for 30 minutes. Thaw TPO, BMP4, Dox and 10% HSA at room temperature. The amount of reagent required is based on Step 3.95 or 3.98.	IMDM media; TPO (50µg); SCF (100 µg); Doxycycline (50 mg); 10% HSA; ITS 100X	Tissue Culture Lab (Grade B)	4-8°C Fridge; -20°C Freezer
Step 3.89	Day 12-18 (10 minutes)	TPO (50 µg); Embryo Water; 15 mL conical tube; P1000 pipette tips; 5 mL stripette	**TPO reconstitution** Using a 5 mL stripette, transfer 4 mL water into a 15 mL conical tubes. Using a p1000 pipette, add 1 mL water to the TPO vial and mix well to resuspend. Using a p1000 pipette transfer the TPO solution into the 15 mL tube containing water and mix well.	TPO 10 µg/mL; Embryo Water; Waste	Tissue Culture (Grade B)	BSC; P1000 pipette; Pipet boy
Step 3.90	Day 12-18 (10 minutes)	SCF (100 µg); Embryo Water; 5 mL stripette; 15 mL conical tubes; P1000 pipette tips	**SCF reconstitution** Using a 5 mL stripette, transfer 1 mL water a 15 mL conical tube. Using a p1000 pipette, add 1 mL water to the SCF vial and mix well to resuspend. Using a p1000 pipette transfer the SCF solution into the 15 mL tube containing water and mix well.	SCF 50 µg/mL; Embryo Water; Waste	Tissue Culture (Grade B)	BSC; P1000 pipette; Pipet boy

Step	Day and Duration	Materials in	Process Step	Materials Out	Location (Room and Grade)	Equipment
Step 3.91	Day 12-18 (10 minutes)	Doxycycline (50 mg)<br>Embryo Water<br>50 mL conical tube<br>P1000 pipette tips<br>50 mL stripette	**Doxycycline reconstitution**<br>Using a 50 mL stripette, transfer 49 mL water into a 50 mL conical tube. Using a p1000 pipette, add 1 mL water to the Doxycycline vial and mix well to resuspend. Using a p1000 pipette transfer the Doxycycline solution into the 50 mL tube containing water and mix well.	Doxycycline 1 mg/mL<br>Embryo Water<br>Waste	Tissue Culture (Grade B)	BSC<br>P1000 pipette<br>Pipet boy
Step 3.92	Day 12-18 (60 minutes)	Plated MK cells (N_{wells})<br>P200 pipette tips (x 1 box)<br>1.5 mL Eppendorf tubes (x N_{plates} / 10)<br><br>MK cells aliquots for counting (N_{plates} /10 x 50 µL)<br>Trypan Blue<br>P10 pipette tips (x1 box)<br>1.5 mL Eppendorf tube (x1)	**Cell counting**<br>Using a P200 pipette, transfer 50 µL cells into a 1.5 mL eppendorf tube. Count 1 every 10 plates.<br><br>**Cell counting**<br>Using a p10 pipette, combine 10 µL cells with 10 µL Trypan Blue in a 1.5 mL Eppendorf tube. Count cells using an haemocytometer<br><br>Cell count >1E06/mL?<br>No → Go to step 3.97<br>YES ↓	Plated MK cells (N_{wells})<br>MK cells aliquots for counting (N_{plates} /10 x 50 µL)<br>Waste<br><br>Total cell number N_{cell}<br>Waste	Tissue Culture (Grade B)	BSC<br>P200 pipette<br>Haemacytometer<br>P10 pipette<br>Calculator

Step	Day and Duration	Materials in	Process Step	Materials Out	Location (Room and Grade)	Equipment
Step 3.93	Day 12-18 (10 minutes)	Total cell number N_{cell}	**Calculation of the required volume of cells to be seeded** Based on the cell number, measured with the haemocytometer, calculate the volume of cells required to seed each well of a 6-well plate at 7.5 E05 cells per well: Vol_{cells} (mL) = (750,000/N_{cell}) Calculate the required amount of MK media to add, to have a final volume of 1.5 mL per well: $Vol_{MKmedia}$ (mL)= (1.5-Vol_{cell})	Cell Volume to be seeded Vol_{cell}; MK media Volume $Vol_{MKmedia}$		Calculator
Step 3.94	Day 12-18 (10 minutes)	MK media Volume $Vol_{MKmedia}$; Number of wells in culture N_{wells}	**Calculation of total MK media volume** To calculate the total volume of MK media required, multiply the volume of MK media per well by the number of wells that are in culture x 1.5: $Vol_{MKmediatotal}$ (mL) = $Vol_{MKmedia}$ x N_{wells} x 1.5	MK media Volume $Vol_{MKmediatotal}$		

Step	Day and Duration	Materials in	Process Step	Materials Out	Location (Room and Grade)	Equipment
Step 3.95	Day 12, 14 (15 minutes)	IMDM media ($Vol_{MKmediatotal}$ mL)<br>10% HSA<br>ITS 100X<br>Beta-mercaptoethanol 55 mM<br>TPO aliquot 10 µg/mL<br>SCF aliquot 50 µg/mL<br>Dox aliquot 1 mg/mL<br>1 L bottles<br>P1000 pipette tips<br>P200 pipette tips<br>100 mL stripettes	**MK media preparation**<br>Using a 100 mL stripette, transfer IMDM media into 1 L bottle(s). Using a 50 mL stripette, add 50 µL/mL of 10% HSA. Using a 10 mL pipette, add 10 µL/mL of ITS 100X. Using a p1000 pipette, add 0.91 µL/mL of Beta-mercaptoethanol to each bottle. Using a p1000 add TPO at 2 µL/mL (20 ng/mL) and SCF at 0.5 µL/mL (25 ng/mL) into each bottle. Using a p200 pipette add 0.031 µL/mL Dox (0.03125 µg/mL) into each bottle.	Supplemented MK media ($Vol_{MKmediatotal}$ mL)<br>Waste	Tissue Culture (Grade B)	BSC<br>Pipette boy<br>P1000 pipette<br>P200 pipette
Step 3.96	Day 12-18 (180 minutes)	Plated MK cells into 6-well plates (N_{wells})<br>Supplemented MK media ($Vol_{MKmediatotal}$ mL)<br>P1000 pipette tips<br>Cell Volume to be seeded Vol_{cell}<br>MK media Volume $Vol_{MKmedia}$	**Cell Seeding**<br>Cell seeding should occur in three parallel batches (by 3 operators). Using a p1000 pipette, transfer $Vol_{MKmedia}$ into each well of a new 6-well plate (N_{wells}). Using a p1000 pipette, resuspend each well containing the cells and transfer Vol_{cell} of cell suspension into a new well containing the media. Top-up each well of the old 6-well plates with Vol_{cell} of media.<br><br>Place the plates in the incubator set at 37˚C, 5% CO_2.<br><br>Is it D20? No → Return to Step 3.88; Yes → Go to Step 3.101	Plated MK cells into 6-well plates (N_{wells} x 2)<br>Waste<br>Return to Step 3.88<br>Go to Step 3.101	Tissue Culture (Grade B)	BSC (x3)<br>Incubator<br>Pipette boy (x3)<br>Aspirator (x3)

Step	Day and Duration	Materials in	Process Step	Materials Out	Location (Room and Grade)	Equipment
Step 3.97	Day 12-18 (10 minutes)	Number of wells in culture N_{wells}	**Calculation of total 2xMK media volume** The volume of 2xMK media required is the same as the number of wells that are in culture: $Vol_{MKmedia}$ (mL) = N_{wells}	MK media Volume $Vol_{MKmedia}$		Calculator
Step 3.98	Day 12-18 (15 minutes)	IMDM media ($Vol_{MKmedia}$ mL); 10% HSA; ITS 100X; Beta-mercaptoethanol 55 mM; TPO aliquot 10 µg/mL; SCF aliquot 50 µg/mL; Dox aliquot 1 mg/mL; 1 L bottles; P1000 pipette tips; P200 pipette tips; 100 mL stripettes	**2x MK media preparation** Using a 100 mL stripette, transfer IMDM media into 1 L bottle(s). Using a 50 mL stripette, add 50 µL/mL of 10% HSA. Using a 10 mL pipette, add 10 µL/mL of ITS 100X. Using a p1000 pipette, add 0.91 µL/mL of Beta-mercaptoethanol to each bottle. Using a p1000 add TPO at 4 µL/mL (40 ng/mL) and SCF at 1 µL/mL (50 ng/mL) into each bottle. Using a p200 pipette add 0.062 µL/mL Dox (0.0625 µg/mL) into each bottle.	Supplemented 2x MK media ($Vol_{MKmedia}$ mL); Waste	Tissue Culture (Grade B)	BSC; Pipette boy; P1000 pipette; P200 pipette

D12-18: MK Media Exchange

	Day and Duration	Materials in	Process Step	Materials Out	Location (Room and Grade)	Equipment
Step 3.99	Day 12-18 (180 minutes)	Plated MK cells into 6-well plates (N_{wells})<br>Supplemented 2xMK media ($Vol_{MKmedia}$)<br>P1000 pipette tips	**Medium exchange**<br>Media exchange should occur in three parallel batches (by 3 operators). Using a P1000 pipette, carefully remove 0.8 mL of old media by tilting the plate. Using a P1000 pipette and tilting the plate, add 1 mL 2X MK media slowly to the edge.<br><br>Place the plate in the incubator set at 37˚C, 5% CO_2.<br><br>Is it D20?<br>No → Return to Step 3.87<br>Yes → Go to Step 3.100	Plated MK cells into 6-well plates (N_{wells})<br>Waste<br>Return to Step 3.87<br>Go to Step 3.100	Tissue Culture (Grade B)	BSC (x3)<br>Incubator<br>Pipette boy (x3)<br>Aspirator (x3)

Step	Day and Duration	Materials in	Process Step	Materials Out	Location (Room and Grade)	Equipment
Step 3.100	Day 20 (15 minutes)	70% IPA	**Reagent spray** Spray accurately all reagents before placing them in the BSC.		Tissue Culture Lab (Grade B)	BSC
Step 3.101	Day 20 (180 minutes)	Plated MK cells (376 x 6-well plates)<br>Phosphate buffered saline<br>10 mL stripette (x 376)<br>1 L Conical centrifuge Tubes (x6)<br>25 mL stripette (x6)<br>100 mL stripette (x1)<br>500 mL Conical centrifuge Tubes (x6)<br>P200 pipette tips (x 1box)<br>1.5 mL Eppendorf tube (x4)	**Cell collection** Cell collection should occur in three parallel batches (by 3 operators). Using a 10 mL stripette, transfer the content of each well into 6 x 1 L conical centrifuge tube. Centrifuge at 120g for 8 minutes at RT (accel3/brake3). Using a 25 mL stripette, add 20 mL PBS to each of the 1 L conical centrifuge tubes and resuspend the cells. Using a 100 mL pipette, mix the cell suspensions into a single 500 mL conical centrifuge tube. Using a p200 pipette, take a 2 x 100 µL aliquots for flow cytometry and 2 x 100 µL aliquots for cell count and transfer it to 4 x 1.5 mL Eppendorf tubes.	MK cells (1 x 120 mL)<br>MK cells aliquot for Flow Cytometry and cell counting (4 x 100 µL)<br>Waste	Tissue Culture (Grade B)	BSC (x3)<br>Pipette boy (x3)<br>P200 pipette<br>Centrifuge

D20: MK collection

Step	Day and Duration	Materials in	Process Step	Materials Out	Location (Room and Grade)	Equipment
Step 3.102	Day 20 (60 minutes)	MK cells aliquot for Flow Cytometry (2 x 100 µL); Flow cytometry antibodies; P200 pipette tips (x 1box)	**Flow Cytometry analysis** Stain 100 µL for flow with CD235aPEcy7, CD42aAPC, CD41APCH7 antibodies. Incubate 20 minutes at RT in the dark. Wash with 500 µL flow buffer (PBS supplemented with 0.5% BSA and 2 mM EDTA). Centrifuge 100 x g, 8 minutes. Discard supernatant and resuspend in 300 µL PBE + Flow count beads (5000 beads, 5 uL) + 1 µg/mL DAPI. Run samples on a flow cytometer. → Is CD41/CD42+ population > 90% → NO: Rejected Product; YES: next step	Flow cytometry data; Waste	Analytical lab	P200 pipette; Flow cytometer
Step 3.103	Day 20 (30 minutes)	MK cells aliquots for counting (2 x 100 µL); Trypan Blue; P10 pipette tips (x1 box); 1.5 mL Eppendorf tube (x1)	**Cell counting** Using a p10 pipette, combine 10 µL cells with 10 µL Trypan Blue in a 1.5 mL Eppendorf tube. Count cells using an haemocytometer	Total cell number N_{cell}; Waste	Tissue Culture (Grade B)	BSC; P200 pipette; Haemacytometer; P10 pipette; Calculator

Step	Day and Duration	Materials in	Process Step	Materials Out	Location (Room and Grade)	Equipment
Step 3.104	Day 20 (10 minutes)	Total cell number N_{cell}	**Calculation of the required volume of cryoprotectant to add** Based on the cell number, measured with the haemocytometer, calculate the volume of cryoprotectant required to resuspend cells at 1 E06 cells/mL: Vol_{cryo} (mL) = (N_{cell} / 1,000,000)	Cryoprotectant Volume Vol_{Cryo}	Tissue Culture (Grade B)	Calculator
Step 3.105	Day 20 (30 minutes)	Cryoprotectant Volume Vol_{Cryo}; MK cells (1 x120 mL); Cryoprotectant; 10 mL stripette (x6)	**Formulation** Centrifuge MK suspension at 120g for 8 mins. Aspirate supernatant and discard. Resuspend in chilled cryoprotectant and mix by trituration using a 10 mL stripette. Maintain cell suspension on cool beads to prevent cell suspension warming.	Formulated MK cells; Waste	Tissue Culture (Grade B)	BSC; Pipette boy; Centrifuge
Step 3.106	Day 20 (30 minutes)	Formulated MK cells; Cryoprotectant; 250 mL Cryobags (x10); Sterile syringe	**Filling** Using a sterile syringe fill each cryobag with 250 mL of cell suspension and place in a cool rack to maintain temperature within 2-8 °C.	Bags filled with formulated MK cells; Waste	Tissue Culture (Grade B)	BSC; Pipette boy; Cool Rack

D20: Freezing

Step	Day and Duration	Materials in	Process Step	Materials Out	Location (Room and Grade)	Equipment
Step 3.107	Day 20 (180 minutes)	Bags filled with formulated MK cells<br>10 mL stripette (x6)	**Freezing** Place cryobags containing formulated MKs into a CRF and initiate the freezing programme. Once complete, transfer to liquid nitrogen for long term storage.	Frozen MK	Cryostorage room (Grade D)	Controlled rate freezer
Step 3.108	Day X (Y minutes)	Frozen MK cells	**Platelet production**	Platelets<br>Waste	Tissue Culture (Grade B)	Device

Supplementary Figure 1: Batch Sequence Diagram of entire process from iPSC to mature MK.

Priority Number (PN) and associated risk description, as a function of priority level (High, Medium, Low), as defined by the FMEA PN Grid Map.

Risk Description (Failure Mode)			
**High Priority**			
Priority Number (PN)		Priority Number (PN)	
2	Wet / Dry Ice / Water - Breach of Sterility	92	Inability to use CHIR99021
5	Breach of Sterility	93	Inconsistent exchange of plates
14	Incomplete/over-dissociation of cells	95	Inconsistent exchange of plates
26	Incomplete/over-dissociation of cells	97	Inconsistent exchange of plates
31	Incomplete/over-dissociation of cells	99	Inconsistent exchange of plates
32	High variability in counting method	101	Inconsistent exchange of plates
35	Impact of DMSO on cells	103	Inconsistent cell collection
44	Incomplete/over-dissociation of cells	104	Pooling
45	High variability in counting method	106	Inconsistent seeding of plates
63	High variability in counting method	108	Inconsistent seeding of plates
64	Heterogenous formulation	109	Uncontrolled thawing of TPO / SCF / Dox
65	Impact of DMSO on cells	110	Inconsistent cell collection
75	Incomplete/over-dissociation of cells	112	Impact of DMSO on cells
76	Impact of DMSO on cells	113	Heterogenous filling
86	Incomplete/over-dissociation of cells	114	Uncontrolled freezing
88	Inconsistent seeding of plates	115	Platelet production
90	Inconsistent exchange of plates		
**Medium Priority**			
1	Wet / Dry Ice / Water - GMP compliance	51	Inconsistent transfection mix formulation
3	Vacuum aspirator	52	Not uniform distribution of transfection mix to cells
4	Daily Observation of Cultures (Over-manipulation of Cultures)	53	Uncontrolled thawing of ROCKi
6	Uncontrolled thawing of VTN and ROCKi	54	Incorrect concentration of antibiotics
7	Inconsistent coating of plates	55	Uncontrolled thawing of ROCKi
8	Uncontrolled thawing of cells during transit	56	Operator error
9	Uncontrolled thawing of cells	57	Operator error
10	Inconsistent cell seeding	58	Uncontrolled thawing of VTN and ROCKi
11	Uncontrolled thawing of VTN and ROCKi	59	Inconsistent coating of plates
12	Uncontrolled thawing of VTN and ROCKi	60	Inconsistent seeding of plates
13	Inconsistent coating of plates	61	Uncontrolled thawing of ROCKi
16	Inconsistent seeding of plates	62	Visual observation
17	Uncontrolled thawing of sgRNA/Cas9	66	Uncontrolled thawing of VTN and ROCKi
18	Inconsistent concentration of sgRNA	67	Inconsistent coating of plates

19	Inconsistent concentration of Cas9	68	Uncontrolled thawing of cells during transit
20	Inconsistent concentration of DNA	69	Uncontrolled thawing of cells
21	Inconsistent transfection mix formulation	70	Inconsistent seeding of plates
22	Not uniform distribution of transfection mix to cells	71	Insufficient/ over-culturing of cells
23	Uncontrolled thawing of ROCKi	72	Uncontrolled thawing of ROCKi
24	Uncontrolled thawing of VTN and ROCKi	73	Uncontrolled thawing of VTN and ROCKi
25	Inconsistent coating of plates	74	Inconsistent coating of plates
27	Daily Observation of Cultures (Over-manipulation of Cultures)	77	Uncontrolled thawing of VTN and ROCKi
28	Uncontrolled thawing of ROCKi	78	Inconsistent coating of plates
29	Uncontrolled thawing of VTN and ROCKi	79	Uncontrolled thawing of cells during transit
30	Inconsistent coating of plates	80	Uncontrolled thawing of cells
33	Inconsistent seeding of plates	81	Inconsistent seeding of plates
34	Freezing medium temperature	82	Insufficient/ over-culturing of cells
36	Uncontrolled thawing of VTN and ROCKi	83	Uncontrolled thawing of ROCKi
37	Inconsistent coating of plates	84	Uncontrolled thawing of VTN and ROCKi
38	Uncontrolled thawing of cells during transit	85	Inconsistent coating of plates
39	Uncontrolled thawing of cells	89	Uncontrolled thawing of FGF2 / BMP4 / Dox
40	Inconsistent seeding of plates	91	Uncontrolled thawing of FGF2 / BMP4 / Dox
41	Uncontrolled thawing of ROCKi	94	Uncontrolled thawing of FGF2 / BMP4 / Dox
42	Uncontrolled thawing of VTN and ROCKi	96	Uncontrolled thawing of FGF2 / BMP4 / Dox
43	Inconsistent coating of plates	98	Uncontrolled thawing of TPO / SCF / Dox
46	Inconsistent seeding of plates	100	Uncontrolled thawing of TPO / SCF / Dox
47	Uncontrolled thawing of sgRNA/Cas9	102	Uncontrolled thawing of TPO / SCF / Dox
48	Inconsistent concentration of sgRNA	107	Uncontrolled thawing of TPO / SCF / Dox
49	Inconsistent concentration of Cas9	115	Platelet production
50	Inconsistent concentration of DNA		
**Low Priority**			
15	High variability in counting method	105	High variability in counting method
87	High variability in counting method	111	High variability in counting method

CATAPULT Cell and Gene Therapy

0

Project Code: Template01x4

Priority Grid # Reference	Process Step (from BSD/PFD)	Potential Failure Mode	Potential Failure Effects	Severity	Potential Cause(s) / Mechanisms of Failure	Occurrence	Current Process Controls (Prevention & Detection)	Detection	RPN	RPC	Further Controls	Method of Implementation	Ease of Implementation	Process Benefit
1	**Raw Material**	Wet / Dry Ice / Water - GMP compliance	Inability to Use	9	Acceptance into the Manufacturing Facility	5	None	1	45	MH	Test alternative technologies for cold storage / temp control	Purchase Kit / Experimental Evaluation	3	3
2	**Raw Material**	Wet / Dry Ice / Water - Breach of Sterility	Batch Failure	9	Raw Material / Cell Material Contamination	5	None	1	45	MH	Test alternative technologies for cold storage / temp control	Purchase Kit / Experimental Evaluation	3	5
3	**Equipment**	Vacuum aspirator	Increased processing times / reduced cell growth or quality	9	Acceptance into the Manufacturing Facility	9	CMO equipment acceptance policy	1	81	HC	Assess impact of alternative approaches (pipetting material)	Experimental evaluation	3	3
4	**Visual Observation**	Daily Observation of Cultures (Over-manipulation of Cultures)	Reduced Cell Expansion / Suboptimal growth	1	Extended duration outside of optimal culture conditions	1	None	5	5	L	Evaluate need for daily monitoring - reduce to key days	Procedularise	1	3
5	**Operator**	Breach of Sterility	Batch Failure	9	Volume of manipulations as a function of no of. consumables & reagents, aliquots sizes & numbers, scaling out process operations, process duration (tiredness)	5	Training	5	225	HC	Appropriately scaled aliquots<br>Scale-up key processing steps<br>Automate processing steps	Experimental Evaluation Process Implementation	3	5
6	**Thaw - HLA Knockout**	Uncontrolled thawing of VTN and ROCKi	Reagent Stability / Quality	1	Time of thaw not defined and/or monitored	5	Visual inspection	5	25	L	Set time, operating window, perform stability studies, for materials that need to be pre-thawed at 4°C / RT, evaluate technologies to perform controlled thawing	Equipment Purchase / Experimental Evaluation	3	3
7	**Thaw - HLA Knockout**	Inconsistent coating of plates	Variability between plates	5	Volume of manipulations as a function of no of. consumables & reagents, aliquots sizes & numbers, scaling out process operations, process duration (tiredness)	9	Training	1	45	MH	Training, automated liquid handling	Equipment Purchase / Experimental Evaluation	3	3
8	**Thaw - HLA Knockout**	Uncontrolled thawing of cells during transit	Batch inconsistency / failure	5	Time to transfer / Too little dry ice / uncontrolled temperature	9	Dry ice transfer	5	225	HC	Set time, operating window, perform stability studies, for materials that need to be pre-thawed at 4°C / RT, evaluate technologies to perform controlled thawing	Equipment Purchase / Experimental Evaluation	3	3
9	**Thaw - HLA Knockout**	Uncontrolled thawing of cells	Cell Stability / Quality	1	Time of thaw not defined and/or monitored	5	Visual inspection	5	25	L	Set time, operating window, perform stability studies, for materials that need to be pre-thawed at 4°C / RT, evaluate technologies to perform controlled thawing	Equipment Purchase / Experimental Evaluation	3	3
10	**Thaw - HLA Knockout**	Inconsistent cell seeding	Batch inconsistency / failure	1	Incorrect volume transfer / non-homogenous cell suspension	1	Visual inspection	5	5	L	Training, automated liquid handling	Equipment Purchase / Experimental Evaluation	3	3
11	**Medium exchange - HLA Knockout**	Uncontrolled thawing of VTN and ROCKi	Reagent Stability / Quality	1	Time of thaw not defined and/or monitored	5	Visual inspection	5	25	L	Set time, operating window, perform stability studies, for materials that need to be pre-thawed at 4°C / RT, evaluate technologies to perform controlled thawing	Equipment Purchase / Experimental Evaluation	3	3

12	**Passage - HLA Knockout**	Uncontrolled thawing of VTN and ROCKi	Reagent Stability / Quality	1	Time of thaw not defined and/or monitored	5	Visual inspection	5	25	L	Set time, operating window, perform stability studies, for materials that need to be pre-thawed at 4°C / RT, evaluate technologies to perform controlled thawing	Equipment Purchase / Experimental Evaluation	3	3
13	**Passage - HLA Knockout**	Inconsistent coating of plates	Variability between plates	5	Volume of manipulations as a function of no of. consumables & reagents, aliquots sizes & numbers, scaling out process operations, process duration (tiredness)	9	Training	1	45	MH	Training, automated liquid handling	Equipment Purchase / Experimental Evaluation	3	3
14	**Passage - HLA Knockout**	Incomplete/over-dissociation of cells	Extended dissociation leading to reduced cell viability or cell quality / Poor cell recovery	5	Improper assessment of dissociation	9	Periodic visual observation	1	45	MH	Alternative passaging methods, automatic pipettors to control shear force	Training, documentation	1	5
15	**Passage - HLA Knockout**	High variability in counting method	Batch inconsistency / failure	5	Low statistical power in counting method / High variability leading to inaccurate calculation of seeding densities	5	Training	5	125	HC	Automated cell counting	Equipment Purchase / Experimental Evaluation	1	1
16	**Passage - HLA Knockout**	Inconsistent seeding of plates	Sub-optimal cell distribution / growth / quality	5	Incorrect volume transfer / non-homogenous cell suspension	1	Visual inspection	5	25	MH	Training, automated liquid handling	Equipment Purchase / Experimental Evaluation	3	3
17	**Transfection - HLA Knockout**	Uncontrolled thawing of sgRNA/Cas9	Reagent Stability / Quality	1	Time of thaw not defined and/or monitored	5	Visual inspection	5	25	L	Set time, operating window, perform stability studies, for materials that need to be pre-thawed at 4°C / RT, evaluate technologies to perform controlled thawing	Equipment Purchase / Experimental Evaluation	3	3
18	**Transfection - HLA Knockout**	Inconsistent concentration of sgRNA	Disruption of formation of transfection	5	Pipetting of low volumes, manipulation of transfectionmix	1	Training	9	45	MH	Training, automated liquid handling	Training/ Equipment purchase	3	3
19	**Transfection - HLA Knockout**	Inconsistent concentration of Cas9	Disruption of formation of transfection	5	Pipetting of low volumes, manipulation of transfectionmix	1	Training	9	45	MH	Training, automated liquid handling	Training/ Equipment purchase	3	3
20	**Transfection - HLA Knockout**	Inconsistent concentration of DNA	Disruption of formation of transfection	5	Pipetting of low volumes, manipulation of transfectionmix	1	Training	9	45	MH	Training, automated liquid handling	Training/ Equipment purchase	3	3
21	**Transfection - HLA Knockout**	Inconsistent transfection mix formulation	Disruption of formation of transfection	5	Pipetting of low volumes, manipulation of transfectionmix	1	Training	9	45	MH	Training, automated liquid handling	Training/ Equipment purchase	3	3
22	**Transfection - HLA Knockout**	Not uniform distribution of transfection mix to cells	Low transfection efficiency	5	Uneven transfection efficiency among different areas of the well	1	Training	9	45	MH	Training, cross-shaped movement of plate while adding transfection mix	Training	3	3
23	**Medium exchange - HLA Knockout**	Uncontrolled thawing of ROCKi	Reagent Stability / Quality	1	Time of thaw not defined and/or monitored	5	Visual inspection	5	25	L	Set time, operating window, perform stability studies, for materials that need to be pre-thawed at 4°C / RT, evaluate technologies to perform controlled thawing	Equipment Purchase / Experimental Evaluation	3	3
24	**Passage - HLA Knockout**	Uncontrolled thawing of VTN and ROCKi	Reagent Stability / Quality	1	Time of thaw not defined and/or monitored	5	Visual inspection	5	25	L	Set time, operating window, perform stability studies, for materials that need to be pre-thawed at 4°C / RT, evaluate technologies to perform controlled thawing	Equipment Purchase / Experimental Evaluation	3	3
25	**Passage - HLA Knockout**	Inconsistent coating of plates	Variability between plates	5	Volume of manipulations as a function of no of. consumables & reagents, aliquots sizes & numbers, scaling out process operations, process duration (tiredness)	9	Training	1	45	MH	Training, automated liquid handling	Equipment Purchase / Experimental Evaluation	3	3
26	**Passage - HLA Knockout**	Incomplete/over-dissociation of cells	Extended dissociation leading to reduced cell viability or cell quality / Poor cell recovery	5	Improper assessment of dissociation	9	Periodic visual observation	1	45	MH	Alternative passaging methods, automatic pipettors to control shear force	Training, documentation	1	5
27	**Clone selection - HLA Knockout**	Daily Observation of Cultures (Over-manipulation of Cultures)	Reduced Cell Expansion / Suboptimal growth	5	Extended duration outside of optimal culture conditions	9	Training / Visual observation	1	45	MH	Evaluate need for daily monitoring - reduce to key days	Procedularise	1	3
28	**Medium exchange - HLA Knockout**	Uncontrolled thawing of ROCKi	Reagent Stability / Quality	1	Time of thaw not defined and/or monitored	5	Visual inspection	5	25	L	Set time, operating window, perform stability studies, for materials that need to be pre-thawed at 4°C / RT, evaluate technologies to perform controlled thawing	Equipment Purchase / Experimental Evaluation	3	3

29	**Passage - HLA Knockout**	Uncontrolled thawing of VTN and ROCKi	Reagent Stability / Quality	1	Time of thaw not defined and/or monitored	5	Visual inspection	5	25	L	Set time, operating window, perform stability studies, for materials that need to be pre-thawed at 4°C / RT, evaluate technologies to perform controlled thawing	Equipment Purchase / Experimental Evaluation	3	3
30	**Passage - HLA Knockout**	Inconsistent coating of plates	Variability between plates	5	Volume of manipulations as a function of no of. consumables & reagents, aliquots sizes & numbers, scaling out process operations, process duration (tiredness)	9	Training	1	45	MH	Training, automated liquid handling	Equipment Purchase / Experimental Evaluation	3	3
31	**Passage - HLA Knockout**	Incomplete/over-dissociation of cells	Extended dissociation leading to reduced cell viability or cell quality / Poor cell recovery	5	Improper assessment of dissociation	9	Periodic visual observation	1	45	MH	Alternative passaging methods, automatic pipettors to control shear force	Training, documentation	1	5
32	**Passage - HLA Knockout**	High variability in counting method	Batch inconsistency / failure	5	Low statistical power in counting method / High variability leading to inaccurate calculation of seeding densities	5	Training	5	125	HC	Automated cell counting	Equipment Purchase / Experimental Evaluation	1	5
33	**Passage - HLA Knockout**	Inconsistent seeding of plates	Sub-optimal cell distribution / growth / quality	5	Incorrect volume transfer / non-homogenous cell suspension	1	Visual inspection	5	25	MH	Training, automated liquid handling	Equipment Purchase / Experimental Evaluation	3	3
34	**Freezing - HLA Knockout**	Freezing medium temperature	Batch inconsistency / failure	5	Uncontrolled temperature	3	Training	1	15	MH	Set time, operating window, perform stability studies, evaluate technologies to control temperature	Equipment Purchase / Experimental Evaluation	3	3
35	**Freezing - HLA Knockout**	Impact of DMSO on cells	Cell Stability / Quality	5	Extended hold time in DMSO	5	Anecdotal	5	125	HC	Set time, operating window, perform stability studies	Training, documentation, Experimental evaluation	3	5
36	**Thaw - Inducible cassette**	Uncontrolled thawing of VTN and ROCKi	Reagent Stability / Quality	1	Time of thaw not defined and/or monitored	5	Visual inspection	5	25	L	Set time, operating window, perform stability studies, for materials that need to be pre-thawed at 4°C / RT, evaluate technologies to perform controlled thawing	Equipment Purchase / Experimental Evaluation	3	3
37	**Thaw - Inducible cassette**	Inconsistent coating of plates	Variability between plates	5	Volume of manipulations as a function of no of. consumables & reagents, aliquots sizes & numbers, scaling out process operations, process duration (tiredness)	9	Training	1	45	MH	Training, automated liquid handling	Equipment Purchase / Experimental Evaluation	3	3
38	**Thaw - Inducible cassette**	Uncontrolled thawing of cells during transit	Batch inconsistency / failure	5	Time to transfer / Too little dry ice / uncontrolled temperature	9	Dry ice transfer	5	225	HC	Set time, operating window, perform stability studies, for materials that need to be pre-thawed at 4°C / RT, evaluate technologies to perform controlled thawing	Equipment Purchase / Experimental Evaluation	3	3
39	**Thaw - Inducible cassette**	Uncontrolled thawing of cells	Cell Stability / Quality	1	Time of thaw not defined and/or monitored	5	Visual inspection	5	25	L	Set time, operating window, perform stability studies, for materials that need to be pre-thawed at 4°C / RT, evaluate technologies to perform controlled thawing	Equipment Purchase / Experimental Evaluation	3	3
40	**Thaw - Inducible cassette**	Inconsistent seeding of plates	Sub-optimal cell distribution / growth / quality	5	Incorrect volume transfer / non-homogenous cell suspension	1	Visual inspection	5	25	MH	Training, automated liquid handling	Equipment Purchase / Experimental Evaluation	3	3
41	**Medium exchange - Inducible cassette**	Uncontrolled thawing of ROCKi	Reagent Stability / Quality	1	Time of thaw not defined and/or monitored	5	Visual inspection	5	25	L	Set time, operating window, perform stability studies, for materials that need to be pre-thawed at 4°C / RT, evaluate technologies to perform controlled thawing	Equipment Purchase / Experimental Evaluation	3	3
42	**Passage - Inducible cassette**	Uncontrolled thawing of VTN and ROCKi	Reagent Stability / Quality	1	Time of thaw not defined and/or monitored	5	Visual inspection	5	25	L	Set time, operating window, perform stability studies, for materials that need to be pre-thawed at 4°C / RT, evaluate technologies to perform controlled thawing	Equipment Purchase / Experimental Evaluation	3	3

43	**Passage - Inducible cassette**	Inconsistent coating of plates	Variability between plates	5	Volume of manipulations as a function of no of. consumables & reagents, aliquots sizes & numbers, scaling out process operations, process duration (tiredness)	9	Training	1	45	MH	Training, automated liquid handling	Equipment Purchase / Experimental Evaluation	3	3
44	**Passage - Inducible cassette**	Incomplete/over-dissociation of cells	Extended dissociation leading to reduced cell viability or cell quality / Poor cell recovery	5	Improper assessment of dissociation	9	Periodic visual observation	1	45	MH	Alternative passaging methods, automatic pipettors to control shear force	Training, documentation	1	5
45	**Passage - Inducible cassette**	High variability in counting method	Batch inconsistency / failure	5	Low statistical power in counting method / High variability leading to inaccurate calculation of seeding densities	5	Training	5	125	HC	Automated cell counting	Equipment Purchase / Experimental Evaluation	1	5
46	**Passage - Inducible cassette**	Inconsistent seeding of plates	Sub-optimal cell distribution / growth / quality	5	Incorrect volume transfer / non-homogenous cell suspension	1	Visual inspection	5	25	MH	Training, automated liquid handling	Equipment Purchase / Experimental Evaluation	3	3
47	**Transfection - Inducible cassette**	Uncontrolled thawing of sgRNA/Cas9	Reagent Stability / Quality	1	Time of thaw not defined and/or monitored	5	Visual inspection	5	25	L	Set time, operating window, perform stability studies, for materials that need to be pre-thawed at 4°C / RT, evaluate technologies to perform controlled thawing	Equipment Purchase / Experimental Evaluation	3	3
48	**Transfection - Inducible cassette**	Inconsistent concentration of sgRNA	Disruption of formation of transfection	5	Pipetting of low volumes, manipulation of transfectionmix	1	Training	9	45	MH	Training, automated liquid handling	Training/ Equipment purchase	3	3
49	**Transfection - Inducible cassette**	Inconsistent concentration of Cas9	Disruption of formation of transfection	5	Pipetting of low volumes, manipulation of transfectionmix	1	Training	9	45	MH	Training, automated liquid handling	Training/ Equipment purchase	3	3
50	**Transfection - Inducible cassette**	Inconsistent concentration of DNA	Disruption of formation of transfection	5	Pipetting of low volumes, manipulation of transfectionmix	1	Training	9	45	MH	Training, automated liquid handling	Training/ Equipment purchase	3	3
51	**Transfection - Inducible cassette**	Inconsistent transfection mix formulation	Disruption of formation of transfection	5	Pipetting of low volumes, manipulation of transfectionmix	1	Training	9	45	MH	Training, automated liquid handling	Training/ Equipment purchase	3	3
52	**Transfection - Inducible cassette**	Not uniform distribution of transfection mix to cells	Low transfection efficiency	5	Uneven transfection efficiency among different areas of the well	1	Training	9	45	MH	Training, cross-shaped movement of plate while adding transfection mix	Training	3	3
53	**Medium exchange - Inducible cassette**	Uncontrolled thawing of ROCKi	Reagent Stability / Quality	1	Time of thaw not defined and/or monitored	5	Visual inspection	5	25	L	Set time, operating window, perform stability studies, for materials that need to be pre-thawed at 4°C / RT, evaluate technologies to perform controlled thawing	Equipment Purchase / Experimental Evaluation	3	3
54	**Killing assay - Inducible cassette**	Incorrect concentration of antibiotics	Incorrect selection of desired cell population	5	Concentration too high or too low	1	Training	5	25	MH	Development activity	Training	1	3
55	**Killing assay - Inducible cassette**	Uncontrolled thawing of ROCKi	Reagent Stability / Quality	1	Time of thaw not defined and/or monitored	5	Visual inspection	5	25	L	Set time, operating window, perform stability studies, for materials that need to be pre-thawed at 4°C / RT, evaluate technologies to perform controlled thawing	Equipment Purchase / Experimental Evaluation	3	3
56	**Killing assay - Inducible cassette**	Operator error	Batch inconsistency / failure	5	Incorrect reagent concentrations	1	Certificate of Analysis (CoA) from Supplier Calculations are proceduralised (e.g. part of BMR)	5	25	MH	Introduce centrifugation of vials (e.g. when powder may accumulate in lid of vial) / Time and temp study / Assess need for pipetting to support reagent dissolution (automated solutions). Training for operators. Introduce QC tests.	Establish Protocol and Procedures	3	3

57	**Homozygous clone selection - Colony picking**	Operator error	Colony mixing	5	Picking of more than one colony with the same tip	3	Colony observation by eye	5	75	HC	Training, use of a suitable microscope	Training/ Equipment purchase	3	3
58	**Passage - Inducible cassette**	Uncontrolled thawing of VTN and ROCKi	Reagent Stability / Quality	1	Time of thaw not defined and/or monitored	5	Visual inspection	5	25	L	Set time, operating window, perform stability studies, for materials that need to be pre-thawed at 4°C / RT, evaluate technologies to perform controlled thawing	Equipment Purchase / Experimental Evaluation	3	3
59	**Passage - Inducible cassette**	Inconsistent coating of plates	Variability between plates	5	Volume of manipulations as a function of no of. consumables & reagents, aliquots sizes & numbers, scaling out process operations, process duration (tiredness)	9	Training	1	45	MH	Training, automated liquid handling	Equipment Purchase / Experimental Evaluation	3	3
60	**Passage - Inducible cassette**	Inconsistent seeding of plates	Sub-optimal cell distribution / growth / quality	5	Incorrect volume transfer / non-homogenous cell suspension	1	Visual inspection	5	25	MH	Training, automated liquid handling	Equipment Purchase / Experimental Evaluation	3	3
61	**Medium exchange - Inducible cassette**	Uncontrolled thawing of ROCKi	Reagent Stability / Quality	1	Time of thaw not defined and/or monitored	5	Visual inspection	5	25	L	Set time, operating window, perform stability studies, for materials that need to be pre-thawed at 4°C / RT, evaluate technologies to perform controlled thawing	Equipment Purchase / Experimental Evaluation	3	3
62	**Freezing - Inducible cassette**	Visual observation	Extended dissociation leading to reduced cell viability or cell quality / Poor cell recovery	5	Ill-defined, qualitative / Dissociation time	9	Training / Visual observation	1	45	MH	Alternative passaging methods, automatic pipettors to control shear force	Training, documentation	3	3
63	**Freezing - Inducible cassette**	High variability in counting method	Batch inconsistency / failure	5	Low statistical power in counting method / High variability leading to inaccurate calculation of seeding densities	5	Training	5	125	HC	Automated cell counting	Equipment Purchase / Experimental Evaluation	1	5
64	**Freezing - Inducible cassette**	Heterogenous formulation	Cell Stability / Quality	5	Dropwise addition of DMSO	1	Anecdotal	9	45	MH	Investigate pre-formulated excipient / Introduce mixing step	Experimental Evaluation Process Implementation	3	5
65	**Freezing - Inducible cassette**	Impact of DMSO on cells	Cell Stability / Quality	5	Extended hold time in DMSO	5	Anecdotal	5	125	HC	Set time, operating window, perform stability studies	Training, documentation, Experimental evaluation	3	5
66	**Thaw - Cell bank generation**	Uncontrolled thawing of VTN and ROCKi	Reagent Stability / Quality	1	Time of thaw not defined and/or monitored	5	Visual inspection	5	25	L	Set time, operating window, perform stability studies, for materials that need to be pre-thawed at 4°C / RT, evaluate technologies to perform controlled thawing	Equipment Purchase / Experimental Evaluation	3	3
67	**Thaw - Cell bank generation**	Inconsistent coating of plates	Variability between plates	5	Volume of manipulations as a function of no of. consumables & reagents, aliquots sizes & numbers, scaling out process operations, process duration (tiredness)	9	Training	1	45	MH	Training, automated liquid handling	Equipment Purchase / Experimental Evaluation	3	3
68	**Thaw - Cell bank generation**	Uncontrolled thawing of cells during transit	Batch inconsistency / failure	5	Time to transfer / Too little dry ice / uncontrolled temperature	9	Dry ice transfer	5	225	HC	Set time, operating window, perform stability studies, for materials that need to be pre-thawed at 4°C / RT, evaluate technologies to perform controlled thawing	Equipment Purchase / Experimental Evaluation	3	3
69	**Thaw - Cell bank generation**	Uncontrolled thawing of cells	Cell Stability / Quality	1	Time of thaw not defined and/or monitored	5	Visual inspection	5	25	L	Set time, operating window, perform stability studies, for materials that need to be pre-thawed at 4°C / RT, evaluate technologies to perform controlled thawing	Equipment Purchase / Experimental Evaluation	3	3
70	**Thaw - Cell bank generation**	Inconsistent seeding of plates	Sub-optimal cell distribution / growth / quality	5	Incorrect volume transfer / non-homogenous cell suspension	1	Visual inspection	5	25	MH	Training, automated liquid handling	Equipment Purchase / Experimental Evaluation	3	3

71	**Passage - Cell bank generation**	Insufficient/ over-culturing of cells	Extended culture leading to reduced cell viability or cell quality	5	Ill-defined, qualitative / Culture time	9	Training / Visual observation	1	45	MH	Automated image analysis tool, metabolite-based in-process control	Training, documentation	3	3
72	**Passage - Cell bank generation**	Uncontrolled thawing of ROCKi	Reagent Stability / Quality	1	Time of thaw not defined and/or monitored	5	Visual inspection	5	25	L	Set time, operating window, perform stability studies, for materials that need to be pre-thawed at 4°C / RT, evaluate technologies to perform controlled thawing	Equipment Purchase / Experimental Evaluation	3	3
73	**Passage - Cell bank generation**	Uncontrolled thawing of VTN and ROCKi	Reagent Stability / Quality	1	Time of thaw not defined and/or monitored	5	Visual inspection	5	25	L	Set time, operating window, perform stability studies, for materials that need to be pre-thawed at 4°C / RT, evaluate technologies to perform controlled thawing	Equipment Purchase / Experimental Evaluation	3	3
74	**Passage - Cell bank generation**	Inconsistent coating of plates	Variability between plates	5	Volume of manipulations as a function of no of. consumables & reagents, aliquots sizes & numbers, scaling out process operations, process duration (tiredness)	9	Training	1	45	MH	Training, automated liquid handling	Equipment Purchase / Experimental Evaluation	3	3
75	**Passage - Cell bank generation**	Incomplete/over-dissociation of cells	Extended dissociation leading to reduced cell viability or cell quality / Poor cell recovery	5	Improper assessment of dissociation	9	Periodic visual observation	1	45	MH	Alternative passaging methods, automatic pipettors to control shear force	Training, documentation	1	5
76	**Freezing - Cell bank generation**	Impact of DMSO on cells	Cell Stability / Quality	5	Extended hold time in DMSO	5	Anecdotal	5	125	HC	Set time, operating window, perform stability studies	Training, documentation, Experimental evaluation	3	5
77	**Thaw - MK Differentiation**	Uncontrolled thawing of VTN and ROCKi	Reagent Stability / Quality	1	Time of thaw not defined and/or monitored	5	Visual inspection	5	25	L	Set time, operating window, perform stability studies, for materials that need to be pre-thawed at 4°C / RT, evaluate technologies to perform controlled thawing	Equipment Purchase / Experimental Evaluation	3	3
78	**Thaw - MK Differentiation**	Inconsistent coating of plates	Variability between plates	5	Volume of manipulations as a function of no of. consumables & reagents, aliquots sizes & numbers, scaling out process operations, process duration (tiredness)	9	Training	1	45	MH	Training, automated liquid handling	Equipment Purchase / Experimental Evaluation	3	3
79	**Thaw - MK Differentiation**	Uncontrolled thawing of cells during transit	Batch inconsistency / failure	5	Time to transfer / Too little dry ice / uncontrolled temperature	9	Dry ice transfer	5	225	HC	Set time, operating window, perform stability studies, for materials that need to be pre-thawed at 4°C / RT, evaluate technologies to perform controlled thawing	Equipment Purchase / Experimental Evaluation	3	3
80	**Thaw - MK Differentiation**	Uncontrolled thawing of cells	Cell Stability / Quality	1	Time of thaw not defined and/or monitored	5	Visual inspection	5	25	L	Set time, operating window, perform stability studies, for materials that need to be pre-thawed at 4°C / RT, evaluate technologies to perform controlled thawing	Equipment Purchase / Experimental Evaluation	3	3
81	**Thaw - MK Differentiation**	Inconsistent seeding of plates	Sub-optimal cell distribution / growth / quality	5	Incorrect volume transfer / non-homogenous cell suspension	1	Visual inspection	5	25	MH	Training, automated liquid handling	Equipment Purchase / Experimental Evaluation	3	3
82	**Seed train - MK Differentiation**	Insufficient/ over-culturing of cells	Extended culture leading to reduced cell viability or cell quality	5	Ill-defined, qualitative / Culture time	9	Training / Visual observation	1	45	MH	Automated image analysis tool, metabolite-based in-process control	Training, documentation	3	3
83	**Seed train - MK Differentiation**	Uncontrolled thawing of ROCKi	Reagent Stability / Quality	1	Time of thaw not defined and/or monitored	5	Visual inspection	5	25	L	Set time, operating window, perform stability studies, for materials that need to be pre-thawed at 4°C / RT, evaluate technologies to perform controlled thawing	Equipment Purchase / Experimental Evaluation	3	3
84	**Passage - MK Differentiation**	Uncontrolled thawing of VTN and ROCKi	Reagent Stability / Quality	1	Time of thaw not defined and/or monitored	5	Visual inspection	5	25	L	Set time, operating window, perform stability studies, for materials that need to be pre-thawed at 4°C / RT, evaluate technologies to perform controlled thawing	Equipment Purchase / Experimental Evaluation	3	3

85	**Passage - MK Differentiation**	Inconsistent coating of plates	Variability between plates	5	Volume of manipulations as a function of no of. consumables & reagents, aliquots sizes & numbers, scaling out process operations, process duration (tiredness)	9	Training	1	45	MH	Training, automated liquid handling	Equipment Purchase / Experimental Evaluation	3	3
86	**Passage - MK Differentiation**	Incomplete/over-dissociation of cells	Extended dissociation leading to reduced cell viability or cell quality / Poor cell recovery	5	Improper assessment of dissociation	9	Periodic visual observation	1	45	MH	Alternative passaging methods, automatic pipettors to control shear force	Training, documentation	1	5
87	**Passage - MK Differentiation**	High variability in counting method	Batch inconsistency / failure	5	Low statistical power in counting method / High variability leading to inaccurate calculation of seeding densities	5	Training	5	125	HC	Automated cell counting	Equipment Purchase / Experimental Evaluation	1	1
88	**Passage - MK Differentiation**	Inconsistent seeding of plates	Sub-optimal cell distribution / growth / quality	5	Volume of manipulations as a function of no of. consumables & reagents, aliquot sizes & numbers, scaling out process operations, process duration (tiredness)	5	Training	5	125	HC	Appropriately scaled aliquots<br>Scale-up key processing steps<br>Automate processing steps	Experimental Evaluation<br>Process Implementation	5	5
89	**<D10 - MK Differentiation**	Uncontrolled thawing of FGF2 / BMP4 / Dox	Reagent Stability / Quality	1	Time of thaw not defined and/or monitored	5	Visual inspection	5	25	L	Set time, operating window, perform stability studies, for materials that need to be pre-thawed at 4°C / RT, evaluate technologies to perform controlled thawing	Equipment Purchase / Experimental Evaluation	3	3
90	**<D10 - MK Differentiation**	Inconsistent exchange of plates	Sub-optimal medium distribution / growth / quality	5	Volume of manipulations as a function of no of. consumables & reagents, aliquot sizes & numbers, scaling out process operations, process duration (tiredness)	5	Training	5	125	HC	Appropriately scaled aliquots<br>Scale-up key processing steps<br>Automate processing steps	Experimental Evaluation<br>Process Implementation	3	5
91	**<D10 - MK Differentiation**	Uncontrolled thawing of FGF2 / BMP4 / Dox	Reagent Stability / Quality	1	Time of thaw not defined and/or monitored	5	Visual inspection	5	25	L	Set time, operating window, perform stability studies, for materials that need to be pre-thawed at 4°C / RT, evaluate technologies to perform controlled thawing	Equipment Purchase / Experimental Evaluation	3	3
92	**<D10 - MK Differentiation**	Inability to use CHIR99021	Inability to Use	9	Restrictive licensing terms	5	None	1	45	MH	Freedom to Operate Searches<br>Liaise early with Licence Holders to understand potential ability to licence	Put in place agreements / contracts	5	5
93	**<D10 - MK Differentiation**	Inconsistent exchange of plates	Sub-optimal medium distribution / growth / quality	5	Volume of manipulations as a function of no of. consumables & reagents, aliquot sizes & numbers, scaling out process operations, process duration (tiredness)	5	Training	5	125	HC	Appropriately scaled aliquots<br>Scale-up key processing steps<br>Automate processing steps	Experimental Evaluation<br>Process Implementation	5	5
94	**<D10 - MK Differentiation**	Uncontrolled thawing of FGF2 / BMP4 / Dox	Reagent Stability / Quality	1	Time of thaw not defined and/or monitored	5	Visual inspection	5	25	L	Set time, operating window, perform stability studies, for materials that need to be pre-thawed at 4°C / RT, evaluate technologies to perform controlled thawing	Equipment Purchase / Experimental Evaluation	3	3
95	**<D10 - MK Differentiation**	Inconsistent exchange of plates	Sub-optimal medium distribution / growth / quality	5	Volume of manipulations as a function of no of. consumables & reagents, aliquot sizes & numbers, scaling out process operations, process duration (tiredness)	5	Training	5	125	HC	Appropriately scaled aliquots<br>Scale-up key processing steps<br>Automate processing steps	Experimental Evaluation<br>Process Implementation	5	5
96	**<D10 - MK Differentiation**	Uncontrolled thawing of FGF2 / BMP4 / Dox	Reagent Stability / Quality	1	Time of thaw not defined and/or monitored	5	Visual inspection	5	25	L	Set time, operating window, perform stability studies, for materials that need to be pre-thawed at 4°C / RT, evaluate technologies to perform controlled thawing	Equipment Purchase / Experimental Evaluation	3	3
97	**<D10 - MK Differentiation**	Inconsistent exchange of plates	Sub-optimal medium distribution / growth / quality	5	Volume of manipulations as a function of no of. consumables & reagents, aliquot sizes & numbers, scaling out process operations, process duration (tiredness)	5	Training	5	125	HC	Appropriately scaled aliquots<br>Scale-up key processing steps<br>Automate processing steps	Experimental Evaluation<br>Process Implementation	5	5
98	**<D10 - MK Differentiation**	Uncontrolled thawing of TPO / SCF / Dox	Reagent Stability / Quality	1	Time of thaw not defined and/or monitored	5	Visual inspection	5	25	L	Set time, operating window, perform stability studies, for materials that need to be pre-thawed at 4°C / RT, evaluate technologies to perform controlled thawing	Equipment Purchase / Experimental Evaluation	3	3

99	**<D10 - MK Differentiation**	Inconsistent exchange of plates	Sub-optimal medium distribution / growth / quality	5	Volume of manipulations as a function of no of. consumables & reagents, aliquot sizes & numbers, scaling out process operations, process duration (tiredness)	5	Training	5	125	HC	Appropriately scaled aliquots Scale-up key processing steps Automate processing steps	Experimental Evaluation Process Implementation	5	5
100	**<D10 - MK Differentiation**	Uncontrolled thawing of TPO / SCF / Dox	Reagent Stability / Quality	1	Time of thaw not defined and/or monitored	5	Visual inspection	5	25	L	Set time, operating window, perform stability studies, for materials that need to be pre-thawed at 4°C / RT, evaluate technologies to perform controlled thawing	Equipment Purchase / Experimental Evaluation	3	3
101	**<D10 - MK Differentiation**	Inconsistent exchange of plates	Sub-optimal medium distribution / growth / quality	5	Volume of manipulations as a function of no of. consumables & reagents, aliquot sizes & numbers, scaling out process operations, process duration (tiredness)	5	Training	5	125	HC	Appropriately scaled aliquots Scale-up key processing steps Automate processing steps	Experimental Evaluation Process Implementation	5	5
102	**IPC - MK Differentiation**	Uncontrolled thawing of TPO / SCF / Dox	Reagent Stability / Quality	1	Time of thaw not defined and/or monitored	5	Visual inspection	5	25	L	Set time, operating window, perform stability studies, for materials that need to be pre-thawed at 4°C / RT, evaluate technologies to perform controlled thawing	Equipment Purchase / Experimental Evaluation	3	3
103	**IPC - MK Differentiation**	Inconsistent cell collection	Batch inconsistency / failure	5	Volume of manipulations as a function of no of. consumables & reagents, aliquot sizes & numbers, scaling out process operations, process duration (tiredness)	5	Training	5	125	HC	Scale-up key processing steps Automate processing steps	Experimental Evaluation Process Implementation	5	5
104	**IPC - MK Differentiation**	Pooling	Batch inconsistency / failure	5	Volume of manipulations as a function of no of. consumables & reagents, aliquot sizes & numbers, scaling out process operations, process duration (tiredness)	5	Training	5	125	HC	Scale-up key processing steps Automate processing steps	Experimental Evaluation Process Implementation	3	5
105	**IPC - MK Differentiation**	High variability in counting method	Batch inconsistency / failure	5	Low statistical power in counting method / High variability leading to inaccurate calculation of seeding densities	5	Training	5	125	HC	Automated cell counting	Equipment Purchase / Experimental Evaluation	1	1
106	**IPC - MK Differentiation**	Inconsistent seeding of plates	Sub-optimal cell distribution / growth / quality	5	Volume of manipulations as a function of no of. consumables & reagents, aliquot sizes & numbers, scaling out process operations, process duration (tiredness)	5	Training	5	125	HC	Appropriately scaled aliquots Scale-up key processing steps Automate processing steps	Experimental Evaluation Process Implementation	5	5
107	**>D10 - MK Differentiation**	Uncontrolled thawing of TPO / SCF / Dox	Reagent Stability / Quality	1	Time of thaw not defined and/or monitored	5	Visual inspection	5	25	L	Set time, operating window, perform stability studies, for materials that need to be pre-thawed at 4°C / RT, evaluate technologies to perform controlled thawing	Equipment Purchase / Experimental Evaluation	3	3
108	**>D10 - MK Differentiation**	Inconsistent seeding of plates	Sub-optimal cell distribution / growth / quality	5	Volume of manipulations as a function of no of. consumables & reagents, aliquot sizes & numbers, scaling out process operations, process duration (tiredness)	5	Training	5	125	HC	Appropriately scaled aliquots Scale-up key processing steps Automate processing steps	Experimental Evaluation Process Implementation	5	5
109	**>D10 - MK Differentiation**	Uncontrolled thawing of TPO / SCF / Dox	Reagent Stability / Quality	5	Volume of manipulations as a function of no of. consumables & reagents, aliquot sizes & numbers, scaling out process operations, process duration (tiredness)	5	Training	5	125	HC	Appropriately scaled aliquots Scale-up key processing steps Automate processing steps	Experimental Evaluation Process Implementation	3	5
110	**IPC - MK Differentiation**	Inconsistent cell collection	Batch inconsistency / failure	5	Volume of manipulations as a function of no of. consumables & reagents, aliquot sizes & numbers, scaling out process operations, process duration (tiredness)	5	Training	5	125	HC	Scale-up key processing steps Automate processing steps	Experimental Evaluation Process Implementation	5	5
111	**IPC - MK Differentiation**	High variability in counting method	Batch inconsistency / failure	5	Low statistical power in counting method / High variability leading to inaccurate calculation of seeding densities	5	Training	5	125	HC	Automated cell counting	Equipment Purchase / Experimental Evaluation	1	1
112	**Freezing - MK Differentiation**	Impact of DMSO on cells	Cell Stability / Quality	5	Extended hold time in DMSO	5	Anecdotal	5	125	HC	Set time, operating window, perform stability studies	Training, documentation, Experimental evaluation	3	5
113	**Freezing - MK Differentiation**	Heterogenous filling	Impact on freezing cycle, poor cell viability and recovery on thaw.	5	Poor mixing of formulated cell suspension	5	None	5	125	HC	Perform stability studies, evaluate technologies to perform homogenous filling	Experimental Evaluation Process Implementation	5	5
114	**Freezing - MK Differentiation**	Uncontrolled freezing	Impact on freezing cycle, poor cell viability and recovery on thaw.	5	Large fill volume and heterogenous distribution within container	5	None	5	125	HC	Perform freezing studies, evaluate technologies to perform controlled rate freezing	Experimental Evaluation Process Implementation	5	5

Supplementary Figure 2: Priority Number (PN) and associated risk description, as a function of priority level (High, Medium, Low), as defined by the Failure Method and Effect Analysis priority number Grid Map (Figure 3c).

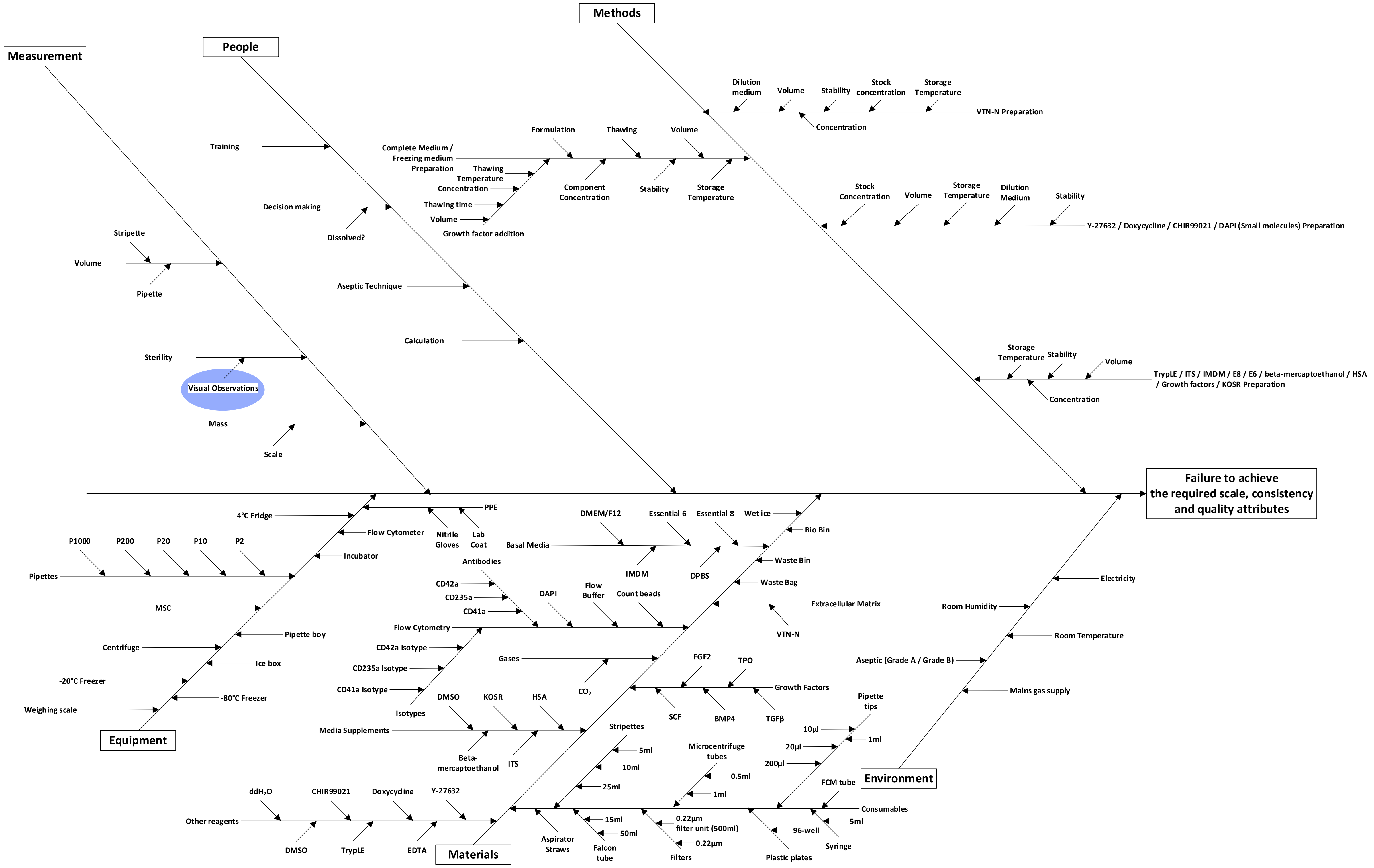

Ishikawa Diagram –Reagent preparation – Root causes of failure (highlighted) with respect to yield and quality.

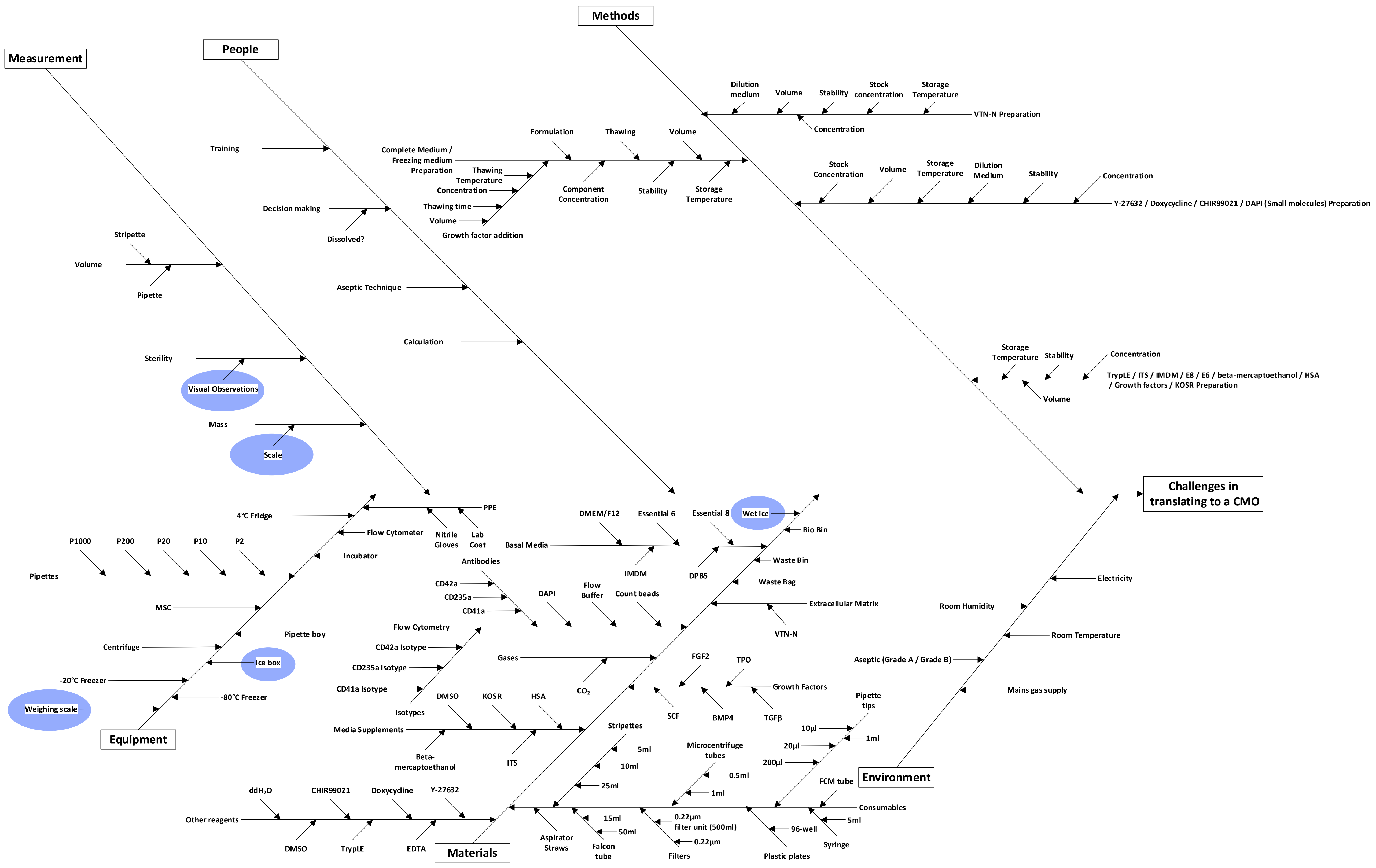

Ishikawa Diagram –Reagent preparation – Root causes of failure (highlighted) with respect to potential transfer to a Contract Manufacturing Organisation (CMO).

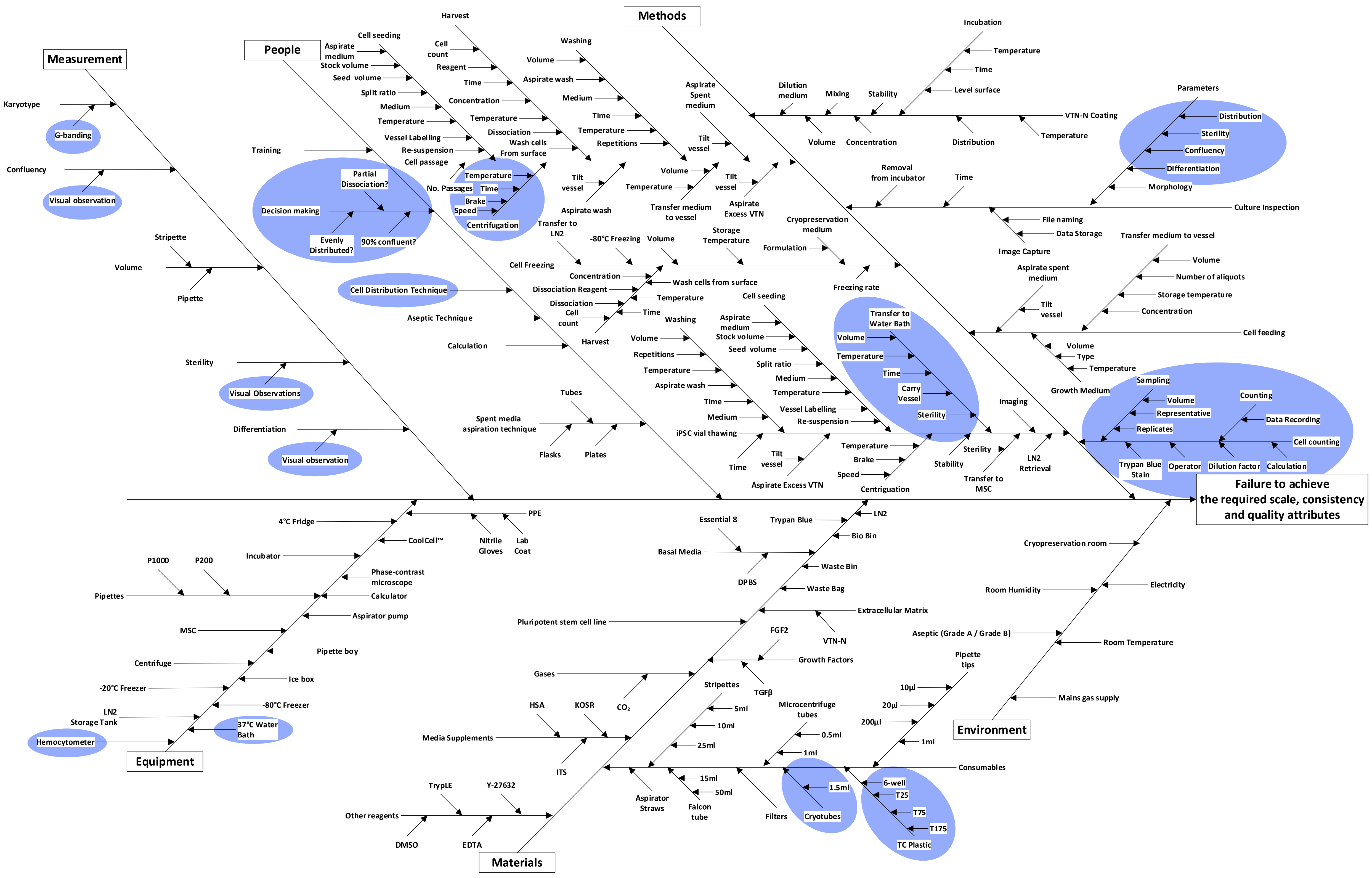

Ishikawa Diagram –PSC Banking and Expansion – Root causes of failure (highlighted) with respect to yield and quality.

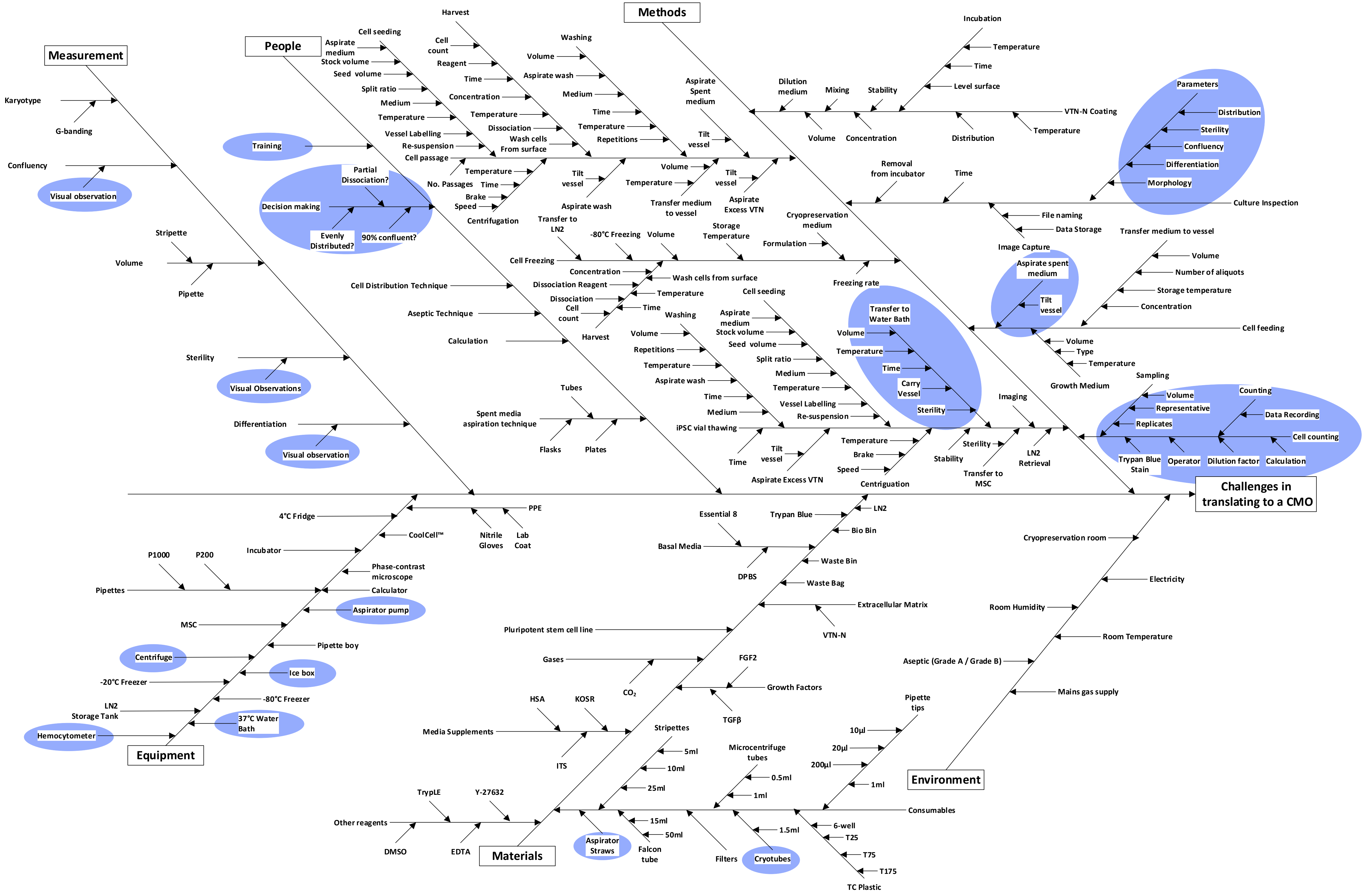

Ishikawa Diagram –PSC Banking and Expansion – Root causes of failure (highlighted) with respect to potential transfer to a Contract Manufacturing Organisation (CMO).

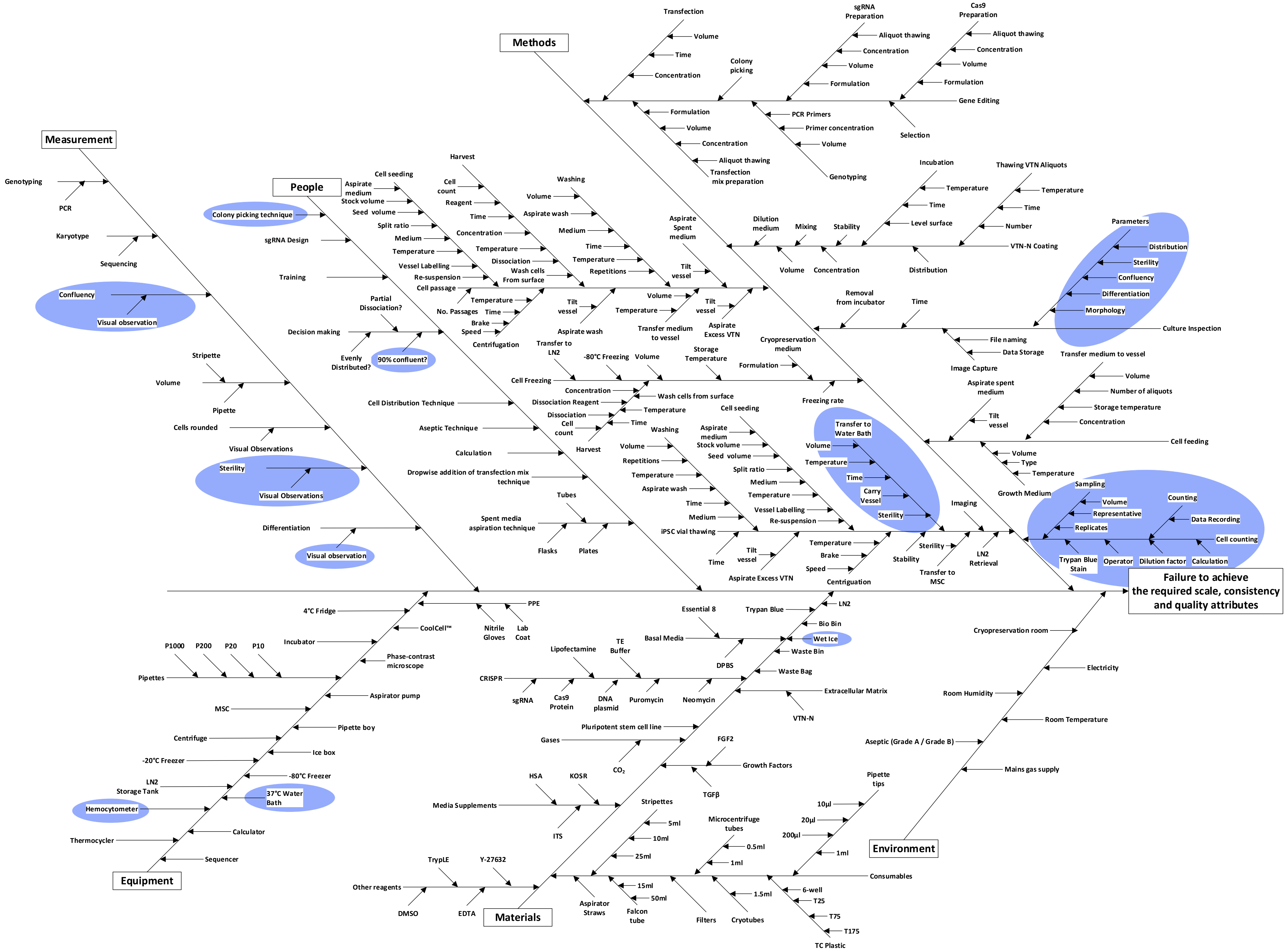

Ishikawa Diagram –PSC Editing – Root causes of failure (highlighted) with respect to yield and quality.

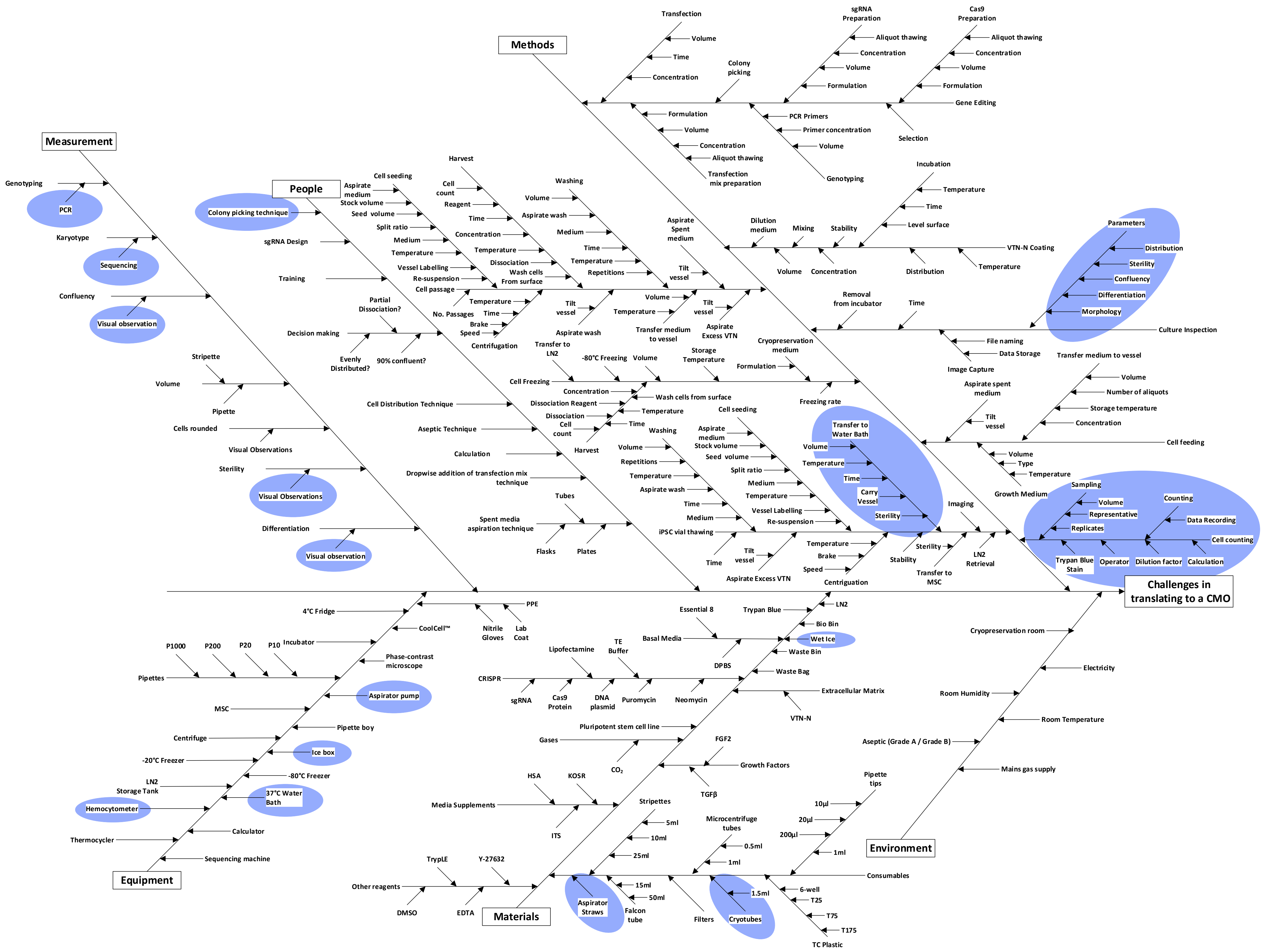

Ishikawa Diagram –PSC Editing – Root causes of failure (highlighted) with respect to potential transfer to a Contract Manufacturing Organisation (CMO).

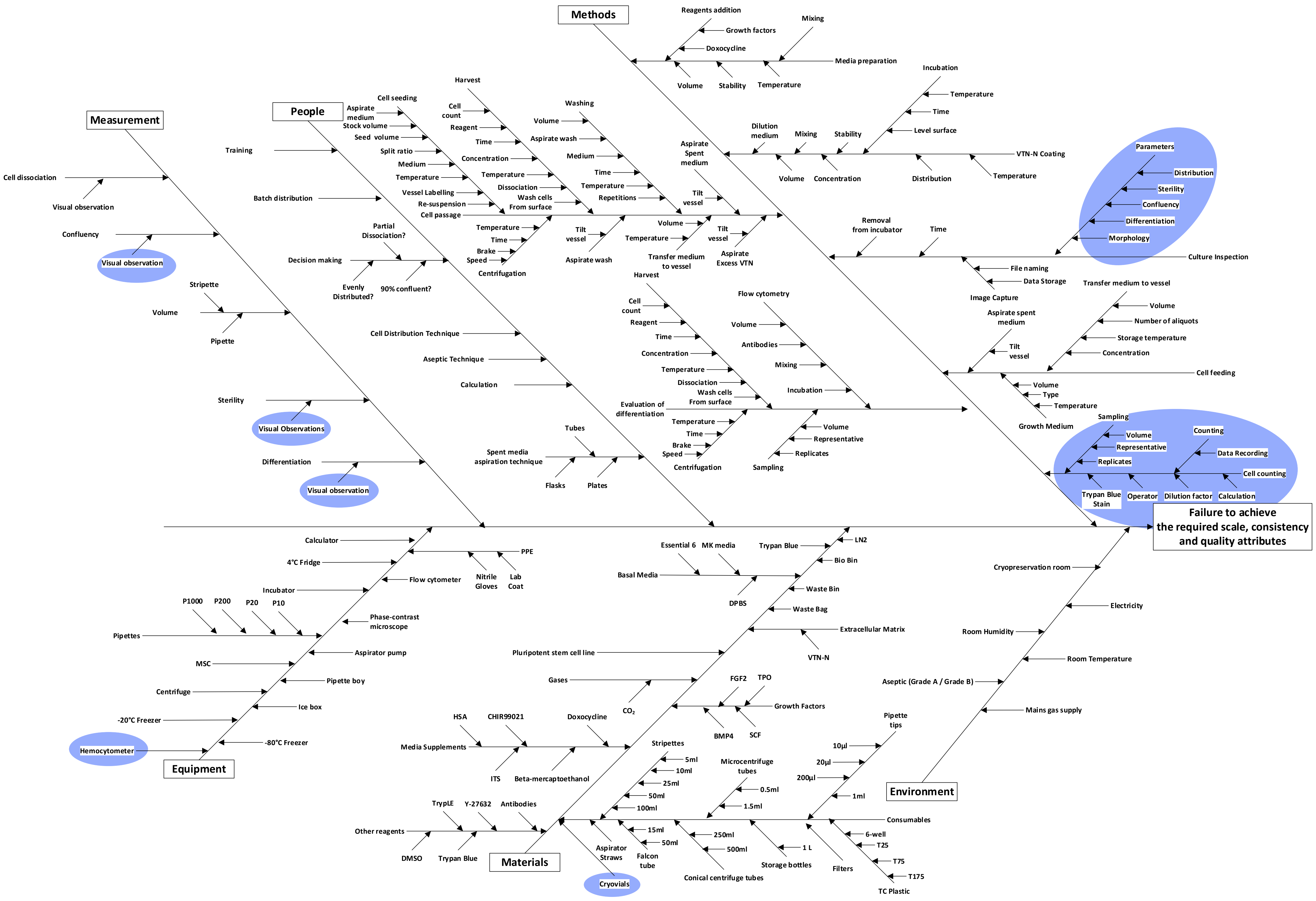

Ishikawa Diagram –Megakaryocyte Differentiation – Root causes of failure (highlighted) with respect to yield and quality.

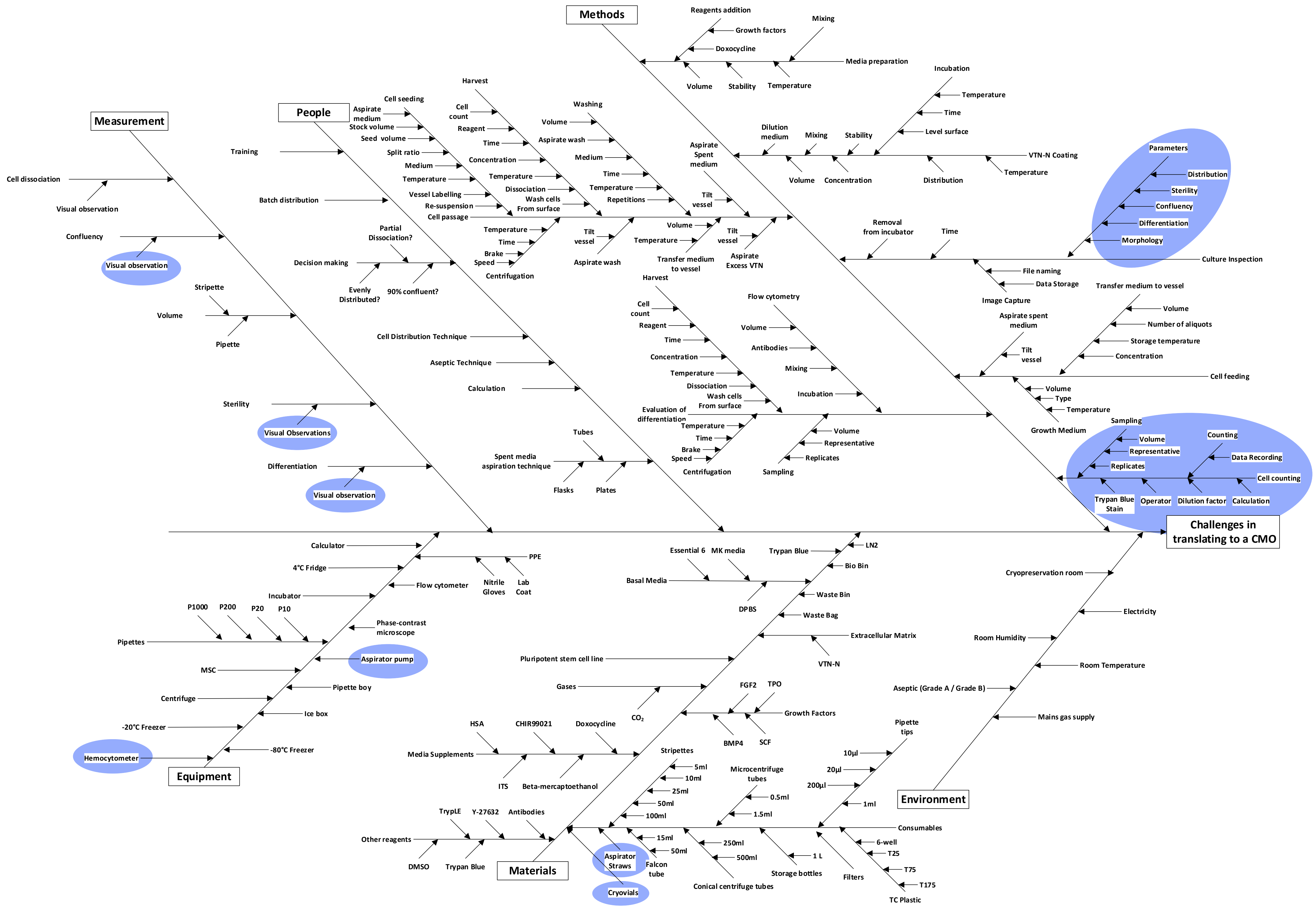

Ishikawa Diagram –Megakaryocyte Differentiation – Root causes of failure (highlighted) with respect to potential transfer to a Contract Manufacturing Organisation (CMO).

Supplementary Figure 3: Ishikawa (Root Cause and Effect) Analysis for reagent preparation, iPSC banking and expansion, iPSC editing and MK differentiation.
